# Community-based house improvement for malaria control in southern Malawi: Stakeholder perceptions, experiences, and acceptability

**DOI:** 10.1371/journal.pgph.0000627

**Published:** 2022-07-14

**Authors:** Tinashe A. Tizifa, Steven Gowelo, Alinune N. Kabaghe, Robert S. McCann, Tumaini Malenga, Richard M. Nkhata, Asante Kadama, Yankho Chapeta, Willem Takken, Kamija S. Phiri, Michele van Vugt, Henk van den Berg, Lucinda Manda-Taylor

**Affiliations:** 1 Division of Internal Medicine, Department of Infectious Diseases, Center for Tropical Medicine and Travel Medicine, University of Amsterdam, Amsterdam University Medical Center, Location Academic Medical Center, Amsterdam, The Netherlands; 2 School of Public Health and Family Medicine, College of Medicine, University of Malawi, Blantyre, Malawi; 3 Laboratory of Entomology, Wageningen University & Research, Wageningen, The Netherlands; 4 Centre for Vaccine Development and Global Health, University of Maryland School of Medicine, Baltimore, Maryland, United States of America; 5 African Institute for Development Policy, Lilongwe, Malawi; 6 Biological Sciences Department, Mzuzu University, Mzuzu, Malawi; PLOS: Public Library of Science, UNITED STATES

## Abstract

House improvement (HI) refers to the full screening or closing of openings such as windows, doors, and eaves, as well as the installation of ceilings, to reduce mosquito-human contact indoors. HI is a viable supplementary intervention that reduces malaria transmission further than the existing strategies alone. In Malawi, HI has not been widely implemented and evaluated for malaria control. Concerns about lack of local evidence, durability in different epidemiological and cultural settings, and the cost of large-scale implementation are among the reasons the strategy is not utilised in many low-income countries. This study assessed community perceptions, experiences, and acceptability of community-led HI in Chikwawa district, southern Malawi. This was a qualitative study where separate focus group discussions were conducted with members from the general community (n = 3); health animators (n = 3); and HI committee members (n = 3). In-depth interviews were conducted with community members (n = 20), and key-informant interviews were conducted with health surveillance assistants and chiefs (n = 23). All interviews were transcribed and coded before performing a thematic content analysis to identify the main themes. Coded data were analysed using Nvivo 12 Plus software. Study participants had a thorough understanding of HI. Participants expressed satisfaction with HI, and they reported enabling factors to HI acceptability, such as the reduction in malaria cases in their villages and the safety and effectiveness of HI use. Participants also reported barriers to effective HI implementation, such as the unavailability and inaccessibility of some HI materials, as well as excessive heat and darkness in HI houses compared to non-HI houses. Participants indicated that they were willing to sustain the intervention but expressed the need for strategies to address barriers to ensure the effectiveness of HI. Our results showed the high knowledge and acceptability of HI by participants in the study area. Intensive and continued health education and community engagement on the significance of HI could help overcome the barriers and improve the acceptability and sustainability of the intervention.

## Introduction

Over the last two decades, significant successes have been achieved in the global fight against malaria [1]. Long-lasting insecticidal nets (LLINs) and indoor residual spraying (IRS), combined with improved diagnosis and effective treatment are among the interventions that have averted about 663 million clinical cases by 2015 [1]. However, these interventions have failed to eliminate malaria in many endemic countries, partly due to residual malaria transmission, even in high-quality intervention coverage areas [2]. The World Health Organization (WHO) 2019 report has shown an increase in malaria cases of about 9 million cases from 2017, with an estimated 228 million malaria cases reported in 2018, and the number of deaths reaching 405 000 [3]. This shows a stagnation of progress in the fight against malaria. For this reason, additional vector control strategies are needed to strengthen the methods currently used in the fight against malaria.

The core malaria vector control strategies include LLINs and IRS. However, many other interventions can be implemented at the household level to significantly reduce mosquito bites in humans. These strategies include the integration and installation of screening on doors, windows, and eaves in addition to other structural modifications or improvements to prevent the entry of adult mosquitoes, which are collectively referred to as house improvement (HI) [4]. The goal of house improvement is to reduce malaria parasite transmission by decreasing mosquito-human contact indoors [4]. Evidence indicates that poor housing is associated with an increased risk of malaria incidence [5]. Small changes or improvements such as screening windows and doors and closing eaves can reduce vector density indoors, potentially reducing the incidence of malaria and other malaria-related complications [6–11]. For example, a trial in the Gambia demonstrated that fully screened houses or just the use of screened ceilings resulted in a 50% reduction in anaemia in children under ten years of age compared to children residing in unscreened housing [8]. Furthermore, studies in Ethiopia, Equatorial Guinea, Gambia, Kenya, and Tanzania have shown that house screening is sustainable and effectively prevents mosquito entry into houses [9–13]. A study in southern Malawi on HI involving partially and fully closed eaves, using locally available materials, revealed that closed eaves resulted in fewer malaria vectors in houses, with differences depending on the degree of eave closure [14].

The contribution of house improvement in reducing malaria in various parts of the world cannot be understated [15,16]. Historically, improved housing was a significant factor in eliminating malaria in the United States of America and its decline in Europe [17]. Today it is described as an important but under-promoted intervention. Preventing mosquitoes from entering homes has additional advantages, such as protecting all household members equally and at all times while indoors and offering protection against other vector-borne diseases through integrated vector control [13]. Therefore, supplemental interventions like HI are needed to reinforce the ongoing control efforts against malaria without causing or increasing insecticide resistance, and this should remain a top priority for sustainable vector control [5,18,19].

In Malawi, like in many other African countries, HI is yet to be introduced for malaria control [7,20,21]. This could be attributed to several factors, including a lack of local evidence for HI in malaria control, its sustainability in different epidemiological and cultural settings, and concerns about the acceptability and the cost of implementation on a large scale. Another possibility is that most national malaria control programmes are aware of the importance of HI but have no means to implement it where it is needed. Therefore, there is a need to consider social, cultural, and economic contexts to evaluate HI’s appropriateness, acceptability, and feasibility to successfully implement intervention programmes such as HI [22].

Possible methods of managing implementation costs and intervention coverage within the local setting could be through community engagement and participation [23]. These strategies involve proactive implementation of the intervention by the local communities [23]. Such approaches could allow adequate coverage of the target areas through community education and skills development on HI. It could also help lower implementation costs, advocate for the intervention as human capital is available locally, and increase community acceptance and responsibility [21,23]. Community engagement and participation are essential tools for controlling endemic disease in low and middle-income countries (LMIC), particularly for the prevention and surveillance strategies [24]. Community participation promotes self-awareness and confidence, prompting people to address their problems and think positively about solutions. It increases the sense of control over issues affecting the lives of community members. Community involvement, however, has been underemphasised in Malawi, thereby providing a shortfall in evidence concerning coverage, acceptability, and uptake of interventions.

The Majete Malaria Project (MMP), a community-led malaria control project, was implemented in southern Malawi to investigate the combined effect of community participation in malaria control through community workshops, HI, and larval source management (LSM) strategies [25,26]. The main study was conducted in villages along the Majete Wildlife Reserve’s perimeter (MWRP) in the Chikwawa district in southern Malawi. The trial interventions, HI and LSM, were implemented as complementary interventions in addition to the Malawi National Malaria Control Programme (NMCP) interventions, and the entire research setting was part of an intensive community education and engagement programme aimed at increasing community participation in malaria control [25,27]. This study aimed to determine the community’s knowledge, perceptions, and acceptability of the community-led implementation of HI. Several studies have been conducted about knowledge and perceptions relating to malaria in Africa, showing that misconceptions of malaria are still prevalent [28–32].

Understanding the evidence on malaria knowledge and perceptions, prevention, and treatment is critical because it can influence the community’s decision on whether or not to participate in malaria control activities [33]. In this study, we describe knowledge and perceptions toward the implementation of HI using qualitative methods, which is vital for programmes that need to assess and modify implementation plans and determine the acceptability and sustainability of an intervention programme. In this study, acceptability is defined as the extent to which people were delivering or receiving a health care intervention. In this case, HI, was thought to be palatable or appropriate based on their experiences with the intervention. Lack of acceptability has long been identified as a barrier to implementation [34]. Data was collected three years post commencement of community participation in HI implementation. The study was motivated by the continued lack of evidence on community involvement in malaria control initiatives within Malawi.

## Materials and methods

### Study design

The study used a cross-sectional study design employing a qualitative approach with focus group discussions (FGDs), in-depth interviews (IDIs), and key informant interviews (KIIs) to assess people’s knowledge, perceptions, and acceptability of the community-led implementation of HI.

### Study setting

As mentioned above, the study was carried out in the communities surrounding the MWRP, located in Chikwawa district (16° 1′ S; 34° 47′ E), 54 km from the commercial city of Blantyre in southern Malawi. The area is host to 90,000 people. The study area has been described in detail elsewhere [35]. The area’s main income activities include subsistence farming of maize, sorghum, millet and beans, livestock rearing, small retail businesses, and brick-making [36].

Agriculture is the key livelihood activity, employing over 80% of the total population [36]. Chikwawa is hot and dry from September to December, hot and rainy from January to April, and mild and dry from June to August. The district is generally dry with typical savannah vegetation. The main public health problems in the area are malaria, diarrhoea, acute respiratory infections (including pneumonia), skin infections, common injuries and wounds, and sexually transmitted diseases [36]. Malaria transmission in this area is predominantly by *Anopheles arabiensis* and *Anopheles funestus*, with a small proportion of *Anopheles gambiae* s.s [27,37,38].

House improvement was implemented in 22 villages as part of a cluster-randomised controlled trial within the MMP’s catchment area from May 2016 through May 2018 as a cluster-randomised trial [25,26] and then continued as a rolled-out intervention in the remaining villages within the MWRP until April 2019. In brief, all of the MMP trial interventions were carried out at the village level, with the trial involving four arms. Villages were randomly assigned to one of these four groups: (a) a control arm, (b) HI, (c) LSM, and (d) HI + LSM [25,27]. All arms used interventions recommended by the NMCP and community engagement [25,27]. This study included all the 22 villages involved with the HI intervention, i.e. the HI and HI+LSM arms. The study villages were divided into three sub-regions, called focal areas (namely A, B, and C), spaced evenly around the MWRP and covering approximately 25,000 people in 65 villages (S1 Fig).

### Study population

We identified five different groups of participants in the qualitative survey, namely: health animators (HAs), HI committee members, members from the broader community, health surveillance assistants (HSAs), and traditional leaders (Chiefs). Traditional leaders are the primary gatekeepers of the communities. They act as a point of contact between the local government and the community and are key players in facilitating development, including promoting health in their communities. In this research project, chiefs oversaw HI implementation in their respective villages.

The duty of HAs was to take a leading role in educating, informing, and promoting malaria control initiatives and implementation of HI in their respective villages. These individuals are volunteers and were selected by chiefs in consultation with their respective community constituents. These individuals received education and training from the MMP and The Hunger Project (THP). The training was in two parts; classroom experience, where knowledge was imparted on various topics concerning malaria prevention and control, and hands-on experience on how HI would be established at the household level using model houses. The roles of health animators are further described elsewhere [23,39,40].

The HI committees comprised 8 to 10 individuals from the respective villages selected by members of each village at community meetings. These HI committees were formed to carry out HI activities in each selected village. They were tasked with storing the materials used in improving households, such as gauze wire, hammers, and measuring tapes. They were responsible for distributing gauze wire in houses requiring it, lobbying for and coordinating community participation in HI implementation at household and village levels. Members of the broader community were responsible for implementing HI in their households, with full eave closure, closing open spaces, and installing gauze wire on the windows.

HSAs form the largest group of community health workers (CHWs) in Malawi. The government deploys them in peri-urban, rural, and hard-to-reach areas to provide and implement preventive, curative, and promotive health programmes [41,42]. In this project, their main task was to promote HI as an intervention for malaria control.

Other potential study participants included HI committee drop-outs (e.g., individuals who once were part of the HI committees but, due to other circumstances, relinquished their positions) and non-participants of the trial (individuals who refused to implement HI). However, these participants (HI committee drop-outs and non-participants) were not available for interviews as some had relocated to other areas and the two non-participants found refused consent.

### Sample size

This was a qualitative study where IDIs, KIIs, and FGDs were conducted. We opted for IDIs because of the depth they guarantee in understanding a social phenomenon [43]. For data triangulation, we used FGDs to stimulate varying responses from various participants [44]. The IDIs with community participants and KIIs were used to supplement the content of the FGDs. Twenty IDIs were conducted with members from the general community in the study villages. Twenty-three key informant interviews were conducted with traditional leaders and HSAs in their respective villages or workstations. Nine mixed-village FGDs (men and women) were undertaken with community members, HAs, and HI committee members drawn from different HI villages. These did not include participants of the IDIs.

### Sampling of study participants

Purposive sampling method was used to select participants in the study villages for the interviews. Purposive sampling was used to facilitate the identification and selection of participants who had adequate information (Information-rich cases) about the topic of interest. Studying these cases yields insights and in-depth understanding rather than empirical generalisations [45]. Community participants, HAs, and HI committee members were purposively selected for the FGDs. Three group discussions (one for each group) were organised in each of the three focal areas. Each group was made up of a minimum of 6 and a maximum of 10 members. The FGDs included group reflections and experiences, shedding more light on the knowledge and perception of HI as an intervention for malaria prevention. Views were also sought from the study participants on the acceptability of HI, which had been implemented in their respective villages.

### Recruitment and training of data collectors

The interviews were conducted by four postgraduates (first, second, sixth, and eighth authors), who were research associates with assistance from six research assistants (Diploma holders) from the MMP. Before the interviews, all data collectors received intense training for one week with guidance from the last author. The highly interactive training included an overview of the study, with an emphasis on the main objective of the study, the study design, qualitative interviewing techniques, and strategies, and participants were encouraged to ask pertinent questions throughout. The consent forms and interview guides were also given to data collectors in English and then translated into *Chichewa*, the local language. The data collectors were trained on the consenting process and the use of digital voice recorders. The data collection tools were piloted on individuals in non-intervention villages to ensure they were clear, relevant, and comprehensive. These individuals from non-intervention villages had similar characteristics to those in the intervention villages.

Questions identified as ambiguous were changed, and questions identified as irrelevant to answering the primary study objectives were omitted. Initial questions for the different interview participants focused on the participants’ experience, impression, and challenges in implementing house improvement. Questions related to health problems in the community, the community’s response towards malaria, and health promotion. However, questions on the role/influence of position were restricted to the chief and HSA KIIs because they were technical and pertained to community leadership, whether administratively for the chiefs or health-related for the HSAs. This section was followed by questions focusing on participants’ knowledge, attitudes, perceptions, and acceptability of house improvement as a malaria control intervention and the perceived benefit for screening eaves, windows, and doors during house improvement. (S1 Text) summarises the interview guides.

### Data collection

Before the interviews, all study participants were booked for face-to-face meetings on the specified date, time, and location. The interviews were conducted at various meeting points at the community level. All FGDs were conducted in private spaces and were held either in a classroom or a private room at the community epicentres. All participants were adults of 18 years and above, composed of both sex and different age groups. IDI and KII administration took around thirty minutes, while the FGD interview took 1.5 to 2 hours. All the interviews were conducted in *Chichewa*. All the potential study participants who were contacted agreed to participate in the study. In total, 47 females and 65 males participated in FGDs, IDIs, and KIIs in the area. We recorded field notes that were subsequently shared and discussed with the whole research team when we completed each day’s task. Data collection took place between 18^th^ March and 20^th^ April 2019.

### Data analysis

All data were audio-recorded, transcribed verbatim, and translated into English by the first author and research assistants. Field notes were continuously recorded, shared, and discussed with research team members as reflections to inform preliminary data analysis at the end-of-day meeting briefs. First, the first author listened to all the audio recordings and read the transcripts multiple times to check the accuracy and understand the issues raised. The first author familiarised himself with the whole dataset to ensure that the data was clean, had proper flow, and accurately conveyed the participants’ responses. Secondly, we used thematic analysis to analyse the data. The first author coded the transcripts sent to the last author for comments and agreement on a common coding framework. A codebook was developed using inductive and deductive coding methods (S2 Text) shows the codebook that was developed. The inductive approach is bottom-up with codes derived from the data, i.e. the participants’ words were used to code the data (in vivo coding) as shown in (S3 Text). At the same time, the deductive approach was based on a predefined set of codes, which guided the coding process [46,47]. These codes were defined from the question guide. The translated transcripts were entered and coded using Nvivo 12 Plus (QSL international, Victoria, Australia). (S4 Text) shows deductive coding results using Nvivo 12 plus. Key themes in the coding framework included the community’s knowledge, perceptions, barriers, facilitators with HI, acceptability, and sustainability of HI. All audios and transcripts were saved in a password-protected computer with access only granted to the researchers. The quotes selected were the most encompassing quotes from all the feedback on the topic. Feedback from different participants was incorporated on that theme to create a balanced representation of the quotes.

### Ethical considerations

Before study implementation, the University of Malawi’s College of Medicine Research and Ethics Committee granted ethical approval (COMREC protocol number P.07/18/2442). The Chikwawa District Health and Social Services (DHSS) office provided permission to collect data in the study villages. Before recruiting participants, we communicated the study to the community through local village heads in liaison with HAs. Written informed consent was obtained from all participants during data collection. All the participants were men and women aged 18 years and above. Literate participants provided a signature on the consent form. Participants that could not read nor write thumb-printed on the consent form after it was read to them in the presence of an impartial witness. Participants were assured that their personal details would be omitted from transcripts, and no personal information would be divulged to ensure confidentiality. Finally, participants were informed that their involvement in the research was voluntary and that withdrawal was permitted at any time and without personal consequence.

## Results

### Socio-demographic characteristic of participants

One hundred twelve (112) people participated in the 52 interview sessions: 43 IDIs and 9 FGDs (Table 1). Most of the participants were in the age group 25 to 44 (58.0%) and reported primary education as their highest level of formal education (51.8%). More males (58.0%) than females (42.0%) participated in the interviews.

**Table 1:** Demographics of study participants.

Characteristic	Focal Area (n)			Total Participants [n, (%)]
	Focal area	Focal area	Focal area	
	A (39)	B (32)	C (41)	112 (100.0%)
**Gender**				
Male	25	19	21	65 (58.0%)
Female	14	13	20	47 (42.0%)
**Age**				
18–24	8	8	9	25 (22.3%)
25–44	21	18	26	65 (58.0%)
≥45	10	6	6	22 (19.6%)
**Education**				
Informal	21	10	4	35 (31.3%)
Primary	13	14	31	58 (51.8%)
Secondary	5	8	6	19 (17.0%)
Tertiary	-	-	-	-
**Session**				
FGD	3	3	3	9 (17.3%)
IDI	6	3	11	20 (38.5%)
KII	10	5	8	23 (44.2%)

### Main themes of the study

There were four main themes drawn through the inductive and deductive methods that emerged from our data: Community knowledge and perceptions of HI as an intervention for preventing malaria, the community experienced barriers and facilitators with implementing HI, acceptability, and sustainability. A description of themes is presented in (Table 2) below.

**Table 2:** Main themes from the qualitative study.

Themes	Data supporting the themes	Researcher’s interpretive summary
Knowledge, perceptions, and experiences with HI	What HI involves Malaria prevention General perceptions, experiences, and concerns The complementary role HI plays in malaria prevention	Community members’ theoretical and practical understanding of HI as an intervention Community members’ interpretation of HI and the implementation experience, e.g., labour and cost on households seeking care.
Barriers and facilitators with HI	Community ownership Community leaders’ involvement and persuasion Capacity building from the project Lack of ventilation, heat, poor lighting, termites in HI houses Inconsistent supply of materials and lack of durability Tensions between the drivers and implementers of HI	Motivating and demotivating factors to community involvement
Acceptability of HI	The appropriateness of the HI intervention	Community members’ approval or disapproval of HI
Sustainability	Considerations and challenges	The willingness to continue to implement HI. Availability, accessibility of materials

### Community knowledge, perceptions, and experiences with HI for malaria prevention

There was widespread knowledge among all participants about HI as an intervention for malaria prevention. HAs and HI committee members received theoretical and practical training on HI and malaria in general. After the initial training in May 2016, they had refresher training two years later, in November 2019. As a result, these groups had a higher level of knowledge about HI than their community counterparts. HSAs, who are technical people in charge of public health, were highly knowledgeable about the intervention. Participants were able to describe how the intervention works and the materials required to build a standard HI house. Almost all participants reiterated that HI prevents malaria by minimising mosquito entry. Interestingly, some community members could even describe the mosquito behaviour on what attracts it to identify the human host inside a house.

*“HI includes closing eaves*, *sleeping under mosquito nets*, *and installing gauze wire at windows*. *Mosquitoes can enter the house to bite the human host by following the smell of the host inside the house*, *and they usually enter through the eaves*.*”* **(IDI, Community participant, FA-A)**

When asked what other procedures are involved in HI, participants gave varied responses. Sealing small holes or spaces on walls and doors was explicitly mentioned to prevent mosquito entry. Some of the participants were able to identify simple and readily available materials such as mud and small pieces of wood to use to close off or seal the open spaces in their homes.

*“HI involves the closure of eaves*. *When we have closed the open eaves*, *sometimes there are still small openings*, *which cannot be closed with bricks; we close them with mud*. *The other thing is that our doors have spaces that can let mosquitoes in*. *So we look for small pieces of timber and fit them in the spaces so that mosquitoes have no entry into the house*.*”* **(FGD, HA, FA-C)**

However, community members’ knowledge of the intervention differed from their experiences with implementing it. They had a good understanding of what HI entails. However, they were surprised by the level of attention to detail and time required for the implementation. People in the community cited reasons such as requiring a lot of manual labour, which would interfere with their day-to-day activities, resulting in less time spent on economic activities as unanticipated and unexpected experiences with the implementation of HI.

*“We discovered that closing eaves and installing gauze wire is time-consuming and exhausting because it necessitates the acquisition of additional materials such as bricks and other equipment*. *We complained to HAs that it would have an impact on our day-to-day work*.*”* **(IDI, Community participant, FA-B)**

However, a thorough engagement with HAs and HI committee members who taught them about the importance of the intervention resulted in a shift in perspective.

*“This work was difficult at first because we did not understand what it was like to close the eaves*. *Afterwards*, *we were taught that closing the eaves helps prevent malaria*. *Once we were taught*, *we began to close the eaves and cracks in the walls of the houses with gauze wire*.*”* **(FGD, Community participant, FA-A)**

Furthermore, people’s experiences with HI changed over time. Their initial experiences with implementing HI turned into delight when they experienced the benefits of the intervention. For example, people linked the closing of eaves and gaps and installing gauze wire in their houses to reduce malaria infection rates in the community and lower household costs for clinic visits.

*“People expressed happiness because firstly*, *malaria cases are minimal*, *people are less sick*. *If they were sick*, *they would go to the Kapichira clinic and pay for the health services*, *but this has reduced*. *Very few cases are going there at the clinic*. *So this money saved by not going to the clinic is now being used for other household developments*.*”* **(KII, HSA, FA-B)**

This experience of having reduced episodes of household illness helped shape their way of thinking because they could establish a link that prevention is less costly than seeking treatment.

We also solicited views from study participants about their knowledge and perceptions of HI as a supplementary malaria intervention and how it complements other interventions. A majority of the community participants knew that ITNs were the main intervention used for malaria prevention. They understood the difference and significance of the two interventions. When asked about the relationship between the two interventions, HAs, HI committee members, HSAs, community participants, and chiefs demonstrated knowledge on how these two interventions are related to malaria control.

*“Yes*, *there is a very strong relationship because when people are using mosquito nets only*, *they get mosquito bites when outside the house or while chatting in the living room before going to bed*. *However*, *when we close the windows and spaces*, *there are few or no mosquitoes inside the house*, *preventing mosquito bites while chatting before going to bed*. *Therefore*, *when they sleep under mosquito nets besides installing gauze wire in windows and spaces*, *there is an increased chance of preventing malaria than using mosquito nets only*.*”* **(KII, HSA, FA-C)**

However, some participants did not understand how the different malaria control intervention tools complement each other, as explained by the HSA below.

*“I can say that this can be a challenge*. *That is the reason I at first said it is surprising that the cases of malaria are increasing*. *I think when people implement HI*, *they think that using mosquito nets is not necessary*. *Many think that because they have improved their houses and do not hear any mosquitoes*, *there is no need to use mosquito nets*.*”* **(KII, HSA, FA-A)**

To understand how HI functions, there is a need to know why and how it acts as a barrier to mosquito entry. Participants were asked if their respective communities were aware of the risk of open eaves for malaria transmission. Several participants knew about this, but one community participant had this to say:

*“Previously*, *people were unaware that when we leave the eaves open*, *mosquitoes can easily enter the house*. *With the arrival of the Majete Malaria Project*, *they realised that by leaving the eaves open*, *they were allowing mosquitoes easy access into the house*.*”* **(FGD, Community participant, FA-B)**

### Factors enabling and impeding the community’s implementation of HI

Factors that motivated or demotivated the implementation of HI were varied. These variations were observed in different groups. However, the main factors influencing the community’s implementation of HI were the influential role community leaders played in promoting HI in the selected focal areas of the study, and community ownership in the implementation of HI was fundamental. Chiefs and other community leaders play a significant role in making critical decisions that affect the well-being of their people. In this study, chiefs mobilised volunteers (HAs and HI committee members) to lead HI implementation. They were also on hand to resolve any problems or disagreements that these volunteers encountered while discharging their duties in the community. They also assisted in the organisation of village meetings led by these volunteers. Community leadership and community ownership appear to have generated a sense of community buy-in for HI.

Most chiefs reported that they played a role in encouraging their people to participate in the intervention while also serving as the last resort for dealing with uncooperative people who were challenging to deal with by the HAs and HI committee members.

“*When the HI committee that we assembled is carrying out its duties within the village and comes across some people who are speaking negatively about the project and are unwilling to participate in any HI-related activity*, *they approach us as chiefs for assistance in dealing with this problem*, *and it is our duty as chiefs to call these people and help them understand so that they can participate*.” **(KII, Chiefs, FA-A)**

In addition, the sense of community ownership in the implementation of HI was also a factor for participating in HI. As one health animator reports:

*“People reacted very positively to this HI intervention*. *Why? Because they were in charge of improving their houses*. *Whenever we were out in the villages*, *people from nearby villages asked*, *“When are you going to start in our village?” This means that people have been positive about the project*.*”* **(FGD, HA, FA-C)**

However, not all community members embraced or took ownership in the implementation of HI. When asked about what they felt were the reasons affecting the community’s support for HI activities, the participants cited several issues. Though not common to all participants, some of the factors cited were that HI causes a lack of ventilation in HI houses resulting from poor air circulation, it impedes the natural light that filters in through the eaves making the houses too dark inside and causing the indoor heat to become extremely uncomfortable.

*“People were saying that there was not enough ventilation in their houses when they closed the eaves*. *So*, *they would not close the eaves and screen their windows with gauze wire*. *They also wanted the light to be getting into the house through the open spaces*.*”* **(FGD, Community participant, FA-C)**

Some HAs stated that the architectural design of most houses within the village, which includes not closing eaves and leaving a gap between the wall and the roof, also aids in termite prevention. Termites would destroy the wood/timber and grass used to build houses if no insecticide was applied. The existence of termites prevented some community members from implementing HI.

*“I experienced that challenge in my village where someone complained about closing open eaves leading to a problem of termites eating poles on the roof because the walls are now in contact with the roof*. *Moreover*, *when fixing the roof*, *they have to remove the bricks they had closed open eaves with*.*”* **(FGD, HA, FA-C)**

Another concern expressed by community members was in the supply and durability of the materials used to seal off their houses. They complained that the gauze wire that they received was rusting and sometimes experienced perforations. They also reported the unavailability of HI equipment at times when they needed it the most.

*“Yes*, *complaints indeed will always occur*, *for instance at first the gauze wire which we received for the windows rusted*, *so people complained that the wire was going bad and was rusting*. *Apart from that*, *people that had newly built houses and those in old houses who wanted to build new houses when they told the HI committee that they were looking for wire gauze*, *they received a response that wire gauze is no longer in stock*.*”* **(KII, HSA, FA-B)**

Lastly, as with many community-based projects, tensions between the drivers (community-based volunteers) and implementers (community members) of a programme bred negative sentiments. Some HAs and HI committee members encountered problems in the execution of their duties. As a result, some community members refused to implement HI in their respective households.

*“At the inception of the project*, *some people had a feeling that the project had a lot of money and accused us of being paid by the project for us to conduct our duties*: *“You are taking the money for yourself*, *you should not fix the gauze wire on the window of my house*.*” This caused friction between us and certain community members such that we had to involve chiefs in some cases to intervene*.*"* **(FGD, HA, FA-C)**

### Acceptability of HI as an approach to malaria control

We solicited the views of study participants regarding the level of acceptability of the HI intervention. The results showed that despite the unanticipated and unexpected experiences with implementing HI, the acceptability of the intervention was high. A majority of the community participants said they were happy that they implemented the intervention. They maintained that nobody forced them to implement this intervention and that they did not regret having it. They reported that they were happy about the intervention and that they were willing to sustain it.

In general, community engagement with HI committees and HAs and the village workshops improved community members’ knowledge, support, and acceptability of HI. They expressed that their experience in helping reduce malaria cases within their villages increased their acceptance of HI. The following excerpts show how stakeholders in the study area describe their happiness and willingness to accept the HI intervention:

*“HI was well received in our villages because*, *at first*, *we were people with no knowledge*, *but after the project arrived*, *they trained HAs and HI committees who educated us on HI*. *Everyone improved their house on their own*, *knowing that we were protecting ourselves from malaria*. *Everyone in the village agreed to do the work and have high-quality HI with properly closed eaves and gauze wire on windows*. *So there is no problem in the village*, *and everyone has accepted this development wholeheartedly and is pleased*.” **(FGD, Community Participants, FA-A)**

*“People were sceptical at the start of the project because they were unfamiliar with the benefits of HI*. *They gradually realised that the money they had previously spent on paying medical bills for family members suffering from malaria was excessive compared to their current situation*, *in which they can now save some money for household developments*. *As a result*, *they accepted the intervention that HI is indeed beneficial*.*”* **(FGD, HI Committee, FA-C)**

Equally important in determining acceptability were the views of the volunteer HAs. Their experience implementing this intervention with the community informed us that it did not interfere with their way of life or violate their social norms.

*“People accepted this intervention*. *The intervention did not cause any harm in our way of living at all to the extent that when we were carrying out our duties in the villages*, *people from neighbouring villages that were not implementing the intervention would ask*, *"When are we going to have this intervention in our village?"* **(FGD, HA, FA-C)**

In short, there was a general perception among some of the community participants that HI as a practice was agreeable within their communities.

### Sustainability and plans for community-led HI

Almost all community members, HAs, HI committee members, chiefs, and HSAs strongly supported the HI intervention and pledged to continue to do so because they believe it has helped reduce the malaria burden in their respective villages. All the participants in this study were confident that their colleagues would be willing to continue implementing the intervention even if the project ceased to exist. Participants believed that the training and skills received by HAs, HI committee members, and themselves would be shared over time and help sustain the programme.

*“I will continue doing this HI work*. *I will not stop even if the project ceases to exist*. *I will encourage people to do HI*, *sleep under a mosquito net*, *empty swamps*, *and drain stagnant water for safety from getting mosquito bites that could lead to malaria*. *I will continue doing this because I am one of the people in the village with the necessary skills*.*”* **(IDI, Community participants, FA-C)**

The availability and accessibility of materials can either help or hinder the implementation of HI. Some community members, HI committee members, and HAs expressed concern that delays in the project’s provision of wire gauze and the unavailability of some resources within their community setting would make it difficult to sustain the intervention.

*“The resources required for HI are not easily accessible or readily available*. *When we go from house to house in the villages*, *we frequently notice houses with open eaves*. *When we contact the house owners*, *we usually find out that many things are required*. *Closing the eaves necessitates the search for bricks*, *nails*, *and other such items*. *This would make it difficult to sustain the intervention*, *particularly in low-income families*.*”* **(FGD, HA, FA-B)**

Participants were asked about how to promote HI to ensure the sustainability of the introduced intervention for malaria control. Some suggested that there should be model houses built within the villages for people to benchmark.

*“To ensure uptake of HI*, *we improve houses that are at strategic points such as along the roads so that they have quality HI*. *We use these houses as demonstration houses so that everyone passing by can see and ask*: *“Whose house is this? How are they doing this? Where are they getting these things from?”* **(FGD, HA, FA-C)**

Others highlighted the significance of sensitisation and continuous education within the communities.

*“HI could be promoted through community sensitisations*, *and continuous meetings and education conducted by health animators where we can educate people on HI*, *so that they may be motivated*.*”* **(IDI, Community participant, FA-B)**

## Discussion

This is the first study in Malawi to examine community stakeholders’ and participants’ perceptions and experiences of participating in a community-based field trial that evaluated house improvement as a complementary approach to reducing mosquito entry, malaria transmission, and malaria cases in an endemic area. The community participants and stakeholders all associated improved housing with reduced malaria transmission. The current study’s findings revealed that knowledge of HI was universal among the study participants, which is attributed to the community involvement in HI-related activities. The relationship between community involvement and malaria awareness is consistent with a study in Rwanda [48] and another study in the same area on LSM that showed that community involvement in LSM as a complementary tool for malaria control increased local awareness of malaria and the control strategy being implemented [23].

While study participants supported the idea of an improved house, our data shows that affordability and accessibility are the two critical concerns to sustainability [49]. Our study findings echoed a study done in Nyabondo, western Kenya that evaluated community knowledge and perceptions on house screening and reported that the second reason for not screening windows, doors, and eaves in the study area is economic/affordability factors household level. The author’s report that “the cost of screening was estimated at approximately 40%, implying that cost is a significant factor for this technology and that community acceptance will be determined by the netting material and user-care dependent durability of the screens [50].”

In addition, the appropriateness of the intervention needs to be considered. Our findings showed that some people were resistant to improving their houses because of poor ventilation. Heat, humidity, poor lighting and ventilation in HI houses and the presence of termites were among the factors that appeared to affect the acceptability and adherence to HI. Similar observations were made in the Gambia in a different study, where there was some negative feedback about house screening regarding thermal comfort, ventilation, or air movement among some study participants [21]. In this rural community and other similar settings within SSA, house designs tend to include eaves to allow adequate ventilation and airflow, minimise heat, and avoid the problem of termites [51–53]. For increased community uptake of the intervention, options including eave tubes could be relevant [54]. Eave tubes are cost-effective [55] and maybe more applicable and relevant in the current setting. In essence, before considering the adoption of HI in these communities, we must consider the context and what cost-effective material and tools can be made readily available, the appropriateness, and the accessibility of housing materials to the rural poor. As Pinder et al. recommend, “housing interventions to keep mosquitoes away and keep the house cool must be customised to local climates and conditions and constructed using high-quality materials” [56]. These considerations could help improve community acceptability.

Community leaders’ influence in facilitating HI’s acceptance played a vital role in maximising community participation in implementation research. The main factors influencing HI acceptance in this study site were community ownership and solidarity, perceived reduction in malaria burden, and community leaders’ involvement and persuasion. The ability for community leaders to positively influence community members is critical for acceptability and sustainability. Our findings mirror other findings within Sub-Saharan Africa (SSA), which found that acceptance was driven by trust in local health authorities and the influence of community leaders [57,58]. Building trust is a crucial feature of deconstructing the demotivators of participation and increasing acceptance. Trust is established through meaningful community engagement, which is open, honest, and transparent and genuinely aims to bring the community together as equal partners in the research process to answer the relevant research questions for the community. In this study, we built our trust with the community by valuing our engagement activities with the community’s gatekeepers and the community as volunteers.

Furthermore, trust among stakeholders is critical to the success of implementation research. It is necessary for facilitating decision-making, acceptability, effectiveness, and cross-learning among actors involved in implementation research. It was demonstrated in this study because the MMP approach was able to integrate different community actors and stakeholders to implement community-led HI, with the project providing technical support and guidance.

The findings clearly show that some stakeholders initially had negative views about the intervention, but through community engagement and interaction, trust was built with their fellow community counterparts, resulting in their shift in perception. The effect of building community trust to achieve intervention effectiveness was demonstrated in Nicaragua following the government’s introduction of mass drug administration (MDA) for malaria control in 1981 [59,60]. The use of MDA resulted in a decrease in malaria incidence. The impact of MDA engendered unprecedented participation in malaria control by both the general population and public health authorities [59,60]. Beginning in 1982, community-based organisations in Nicaragua aided in promoting improved sanitation, including control of vector breeding sites. They delivered educational programmes to improve case finding and followed-up patients to increase treatment adherence [59,60]. These efforts were made to recognise that trust was required to ensure an effective intervention, address service gaps, and influence policymaking. Building trust among various stakeholders has been shown to affect acceptability in implementation research. In an implementation study examining the effect of the school-engaged social and behaviour change communication (SBCC) approach on malaria prevention in Ethiopia, it was discovered that the strategy was acceptable and feasible to the stakeholders [61]. However, the acceptability of the strategy was dependent on building trust among stakeholders and the strategy’s effectiveness in combating malaria in the area [61]. Working with the chiefs and community volunteers created advocates for the intervention while simultaneously promoting buy-in.

We enhanced trust through our community engagement activities. As we have shown in this study, community engagement is critical for the success of any intervention or the adoption of new strategies to improve health. Success in the uptake of HI was demonstrated in our findings. As such, community engagement should be considered in planning efforts of control strategies to increase community awareness and participation while also addressing existing gaps in implementation. Bottom-up community engagement has improved uptake and adoption of interventions and is vital for promoting behaviour change [62,63]. This study was carried out in a setting where community engagement was central to intervention implementation via trained local volunteers (HAs and HI/LSM committee members) [26]. The community engagement strategy was used to garner the support of officials and community leaders and gain access to the research site before gradually introducing the entire process to the broader community.

To scale up malaria control interventions, efforts must identify or pay attention to household and community-level obstacles and aim to navigate these barriers and turn them into opportunities to create change. Our study has revealed that barriers to use were there, but there were facilitators to use that present opportunity for future large-scale uptake, as discussed above. With this in mind, we urge that for malaria control programmes to maximise research findings, facilitate the translation of knowledge into policy adoption and ensure that there is a return on research funder investment, key messages about HI should be communicated verbally and visually through face-to-face interactions, such as community workshops and mass (sensitisation) campaigns and the development of posters. Educational messages must be culturally sensitive and capitalise on existing positive beliefs and behaviours in local communities. This statement is supported by the findings of a study conducted in the same area, which investigated the experiences of community health animators in malaria control and reported that the HA model could be used effectively as a form of information, education, and communication (IEC) to supplement malaria control interventions [64]. The support of the village’s leadership and the health system was also critical in legitimising the main messages [64]. Therefore, more focused messages and health education should be provided to improve understanding of the intervention and alleviate concerns of communities that use HI in the future. The educational methods can be developed when considering advocacy for house improvement as a complementary malaria control tool in Malawi.

### Recommendations

These findings have significant implications for malaria control in SSA. We developed the following policy recommendations based on a policy framework proposed by Walt and Gilson [65] that describes key elements (content, process, context, and actors) that influence policy development and implementation. The strategy involving the capacity building of community volunteers by MMP on HI and malaria, community mobilisation, and sensitisation by chiefs, substantially improved knowledge and understanding of malaria and HI. It also enhanced participation, increased acceptability of the intervention, and improved community trust among various community stakeholders. This strategy is recommended for widescale implementation in rural areas where malaria is endemic. Implementing community-led HI involved various actors within the community, with each actor playing a pivotal role in the implementation process. This is a recommendation to the programme planners and policymakers involved in malaria control to consider the involvement of various actors at the community level using the bottom-up approach of community engagement to enhance intervention buy-in. The scale-up of community-led HI would necessitate policymakers to address the logistical supply of materials such as gauze wire, price regulation, and quality monitoring of these supplies. Communities should be informed of the cost and availability of these materials. If adopted by the government, it should be recommended that a proper mechanism for ensuring access and equity of resources, particularly in rural areas, be implemented.

### Limitations

Participants such as HI committee drop-outs and trial non-participants either refused consent or were unavailable due to relocation. It would have been preferable to have the perspectives of these participants in this study for the significance of having alternative viewpoints. Additionally, the study was conducted by a team of investigators affiliated with MMP. It could be possible that the investigators’ background may have influenced participant responses. Furthermore, purposive selection of participants who had adequate information about the topic may have biased the results, which may not have been representative of the population.

## Conclusion

This study adds to the body of evidence regarding malaria prevention in rural community settings and promotes community buy-in to interventions. The study showed that the community-led HI resonated positively amongst the population in the rural area of Chikwawa in Malawi. Community-led HI implementation improved the community’s knowledge on HI and malaria. Participants perceived that HI had contributed to reducing the burden of malaria in the area. Acceptability of the intervention was reported to be high by participants. However, barriers towards implementation, such as heat and lack of ventilation in the HI house, inaccessibility, and affordability of materials, would make some community members uninterested in the intervention. For the intervention to be sustained, there is a need for intensive IEC on HI, focusing on the importance of HI, people’s roles, and responsibilities. Community engagement would also help to improve the implementation of a smooth intervention.

## Supporting information

S1 Fig Map of the Majete Wildlife Reserve and the perimeter. This map shows the study site and the villages where HI was implemented.
(TIFF)

S1 Text Interview guides. These qualitative interview guides show the questions that guided the interviews in this study.
(DOCX)

S2 Text Codebook. This guided the coding process.
(DOCX)

S3 Text Inductive coding. Codes that were derived from the data using participants’ words.
(DOCX)

S4 Text Deductive coding. Codes that were derived from the question (interview) guide.
(DOCX)

## References

[1] Bhatt S, Weiss DJ, Cameron E, Bisanzio D, Mappin B, Dalrymple U, et al. The effect of malaria control on Plasmodium falciparum in Africa between 2000 and 2015. Nature. 2015;526(7572):207–211. doi: 10.1038/nature15535 . 26375008 PMC4820050

[2] Killeen GF. Characterising, controlling, and eliminating residual malaria transmission. Malar J. 2014;13:330. doi: 10.1186/1475-2875-13-330 . 25149656 PMC4159526

[3] World Health Organization. World malaria report 2019. Geneva. World Health Organization; 2019. ISBN: 978-92-4-156572-1.

[4] Tizifa TA, Kabaghe AN, McCann RS, van den Berg H, van Vugt M, Phiri KS. Prevention Efforts for Malaria. Curr Trop Med Rep. 2018;5(1): 41–50. doi: 10.1007/s40475-018-0133-y . 29629252 PMC5879044

[5] Tusting LS, Ippolito MM, Willey BA, Kleinschmidt I, Dorsey G, Gosling RD, et al. The evidence for improving housing to reduce malaria: a systematic review and meta-analysis. Malar J. 2015;14:209. doi: 10.1186/s12936-015-0724-1 . 26055986 PMC4460721

[6] Lindsay SW, Jawara M, Paine K, Pinder M, Walraven GEL, Emerson PM. Changes in house design reduce exposure to malaria mosquitoes. Trop Med Int Health. 2003;8(6): 512–517. doi: 10.1046/j.1365-3156.2003.01059.x . 12791056

[7] Kirby MJ, Green C, Milligan PM, Sismanidis C, Jasseh M, Conway DJ, et al. Risk factors for house-entry by malaria vectors in a rural town and satellite villages in The Gambia. Malar J. 2008;7:2. doi: 10.1186/1475-2875-7-2 . 18179686 PMC2267476

[8] Kirby MJ, Ameh D, Bottomley C, Green C, Jawara M, Milligan PJ, et al. Effect of two different house screening interventions on exposure to malaria vectors and on anaemia in children in The Gambia: a randomised controlled trial. Lancet. 2009;374(9694):998–1009. doi: 10.1016/S0140-6736(09)60871-0 . 19732949 PMC3776946

[9] Atieli H, Menya D, Githeko A, Scott T. House design modifications reduce indoor resting malaria vector densities in rice irrigation scheme area in western Kenya. Malar J. 2009;8:108. doi: 10.1186/1475-2875-8-108 . 19454025 PMC2688520

[10] Massebo F, Lindtjørn B. The effect of screening doors and windows on indoor density of Anopheles arabiensis in South-West Ethiopia: a randomised trial. Malar J. 2013;12:319. doi: 10.1186/1475-2875-12-319 . 24028542 PMC3847214

[11] Ogoma SB, Lweitoijera DW, Ngonyani H, Furer B, Russell TL, Mukabana WR, et al. Screening mosquito house entry points as a potential method for integrated control of endophagic filariasis, arbovirus, and malaria vectors. PLoS Negl Trop Dis. 2010;4(8):e773. doi: 10.1371/journal.pntd.0000773 . 20689815 PMC2914752

[12] Ogoma SB, Kannady K, Sikulu M, Chaki PP, Govella NJ, Mukabana WR, et al. Window screening, ceilings and closed eaves as sustainable ways to control malaria in Dar es Salaam, Tanzania. Malar J. 2009;8:221. doi: 10.1186/1475-2875-8-221 . 19785779 PMC2760565

[13] Bradley J, Rehman AM, Schwabe C, Vargas D, Monti F, Ela C, et al. Reduced prevalence of malaria infection in children living in houses with window screening or closed eaves on Bioko Island, Equatorial Guinea. PLoS ONE. 2013;8(11):e80626. doi: 10.1371/journal.pone.0080626 . 24236191 PMC3827414

[14] Mburu MM, Juurlink M, Spitzen J, Moraga P, Hiscox A, Mzilahowa T, et al. Impact of partially and fully closed eaves on house entry rates by mosquitoes. Parasit Vectors. 2018;11(1):383. doi: 10.1186/s13071-018-2977-3 . 29970153 PMC6029021

[15] Lwetoijera DW, Kiware SS, Mageni ZD, Dongus S, Harris C, Devine GJ, et al. A need for better housing to further reduce indoor malaria transmission in areas with high bed net coverage. Parasit Vectors. 2013;6:57. doi: 10.1186/1756-3305-6-57 . 23497471 PMC3599311

[16] Carter AD. Are housing improvements an effective supplemental vector control strategy to reduce malaria transmission? A Systematic Review. MPH. Thesis, Georgia State University. 2014. Available from: https://scholarworks.gsu.edu/iph_theses/327/.

[17] Zhao X, Smith DL, Tatem AJ. Exploring the spatiotemporal drivers of malaria elimination in Europe. Malar J. 2016;15:122. doi: 10.1186/s12936-016-1175-z . 26944257 PMC4778289

[18] The malERA Consultative Group on Vector Control. A research agenda for malaria eradication: vector control. PLoS Med. 2011;8(1):e1000401. doi: 10.1371/journal.pmed.1000401 . 21311587 PMC3026704

[19] Hemingway J, Beaty BJ, Rowland M, Scott TW, Sharp BL. The Innovative Vector Control Consortium: improved control of mosquito-borne diseases. Trends Parasitol. 2006;22(7):308–312. doi: 10.1016/j.pt.2006.05.003 . 16713358

[20] Kirby MJ, West P, Green C, Jasseh M, Lindsay SW. Risk factors for house-entry by culicine mosquitoes in a rural town and satellite villages in The Gambia. Parasit Vectors. 2008;1(1):41. doi: 10.1186/1756-3305-1-41 . 18939969 PMC2584634

[21] Kirby MJ, Bah P, Jones COH, Kelly AH, Jasseh M, Lindsay SW. Social acceptability and durability of two different house screening interventions against exposure to malaria vectors, Plasmodium falciparum infection, and anemia in children in The Gambia, West Africa. Am J Trop Med Hyg. 2010;83(5):965–972. doi: 10.4269/ajtmh.2010.10-0311 . 21036822 PMC2963954

[22] Jones C, Williams HA. Social sciences in malaria control. Trends Parasitol. 2002;18(5):195–196. doi: 10.1016/s1471-4922(02)02272-9 . 11983590

[23] Gowelo S, McCann RS, Koenraadt CJM, Takken W, van den Berg H, Manda-Taylor L. Community factors affecting participation in larval source management for malaria control in Chikwawa District, southern Malawi. Malar J. 2020;19(1):195. doi: 10.1186/s12936-020-03268-8 . 32487233 PMC7265157

[24] Dias JC. Community participation and control of endemic diseases in Brazil: problems and possibilities. Cadernos de Saúde Pública. 1998;14:19–37. 10.1590/S0102-311X1998000600003 . 9700223

[25] McCann RS, van den Berg H, Diggle PJ, van Vugt M, Terlouw DJ, Phiri KS, et al. Assessment of the effect of larval source management and house improvement on malaria transmission when added to standard malaria control strategies in southern Malawi: study protocol for a cluster-randomised controlled trial. BMC Infect Dis. 2017;17(1):639. doi: 10.1186/s12879-017-2749-2 . 28938876 PMC5610449

[26] van den Berg H, van Vugt M, Kabaghe AN, Nkalapa M, Kaotcha R, Truwah Z, et al. Community-based malaria control in southern Malawi: a description of experimental interventions of community workshops, house improvement, and larval source management. Malar J. 2018;17:1–12. doi: 10.1186/s12936-018-2415-1 30012147 PMC6048888

[27] McCann RS, Kabaghe AN, Moraga P, Gowelo S, Mburu MM, Tizifa T, et al. The effect of community-driven larval source management and house improvement on malaria transmission when added to the standard malaria control strategies in Malawi: a cluster-randomised controlled trial. Malar J. 2021;20(1):232. doi: 10.1186/s12936-021-03769-0 . 34022912 PMC8140568

[28] Govere J, Durrheim D, La Grange K, Mabuza A, Booman M. Community knowledge and perceptions about malaria and practices influencing malaria control in Mpumalanga Province, South Africa. S Afr Med J. 2000;90(6):611–616. https://www.ajol.info/index.php/samj/article/view/157669 10918892

[29] Mboera LEG, Shayo EH, Senkoro KP, Rumisha SF, Mlozi MRS, Mayala BK. Knowledge, perceptions, and practices of farming communities on linkages between malaria and agriculture in Mvomero District, Tanzania. Acta Trop. 2010;113(2):139–144. doi: 10.1016/j.actatropica.2009.10.008 . 19854143

[30] Hlongwana KW, Mabaso MLH, Kunene S, Govender D, Maharaj R. Community knowledge, attitudes and practices (KAP) on malaria in Swaziland: A country earmarked for malaria elimination. Malar J. 2009;8:29. doi: 10.1186/1475-2875-8-29 . 19228387 PMC2649151

[31] Vundule C, Mharakurwa S. Knowledge, practices, and perceptions about malaria in rural communities of Zimbabwe: relevance to malaria control. Bull World Health Organ. 1996;74(1):55–60. . 8653816 PMC2486838

[32] Wanzala P, Hassanali J, Kibet P, Dossajee H. Perceptions of primary health care with regard to corresponding knowledge, attitude, and practices amongst the Kenyan Maasai. East Afr Med J. 2005;82(1):24–27. doi: 10.4314/eamj.v82i1.9290 . 16122108

[33] Atkinson JAM, Fitzgerald L, Toaliu H, Taleo G, Tynan A, Whittaker M, et al. Community participation for malaria elimination in Tafea Province, Vanuatu: Part I. Maintaining motivation for prevention practices in the context of disappearing disease. Malar J. 2010;9:93. doi: 10.1186/1475-2875-9-93 . 20380748 PMC2873527

[34] Davis FD. User acceptance of information technology: system characteristics, user perceptions, and behavioral impacts. Int J Man Mach Stud. 1993;38:475–487. 10.1006/imms.1993.1022.

[35] Kabaghe AN, Chipeta MG, McCann RS, Phiri KS, van Vugt M, Takken W, et al. Adaptive geostatistical sampling enables efficient identification of malaria hotspots in repeated cross-sectional surveys in rural Malawi. PLoS ONE. 2017;12(2):e0172266. doi: 10.1371/journal.pone.0172266 . 28196105 PMC5308819

[36] Chikwawa district council socioeconomic profile 2017–2022. 2017. Available from: https://www.integrationpoint.mw/content/chikwawa-district-council-socio-economic-profile-2017-2022.

[37] Mburu MM, Zembere K, Mzilahowa T, Terlouw AD, Malenga T, van den Berg H, et al. Impact of cattle on the abundance of indoor and outdoor resting malaria vectors in southern Malawi. Malar J. 2021;20(1):353. doi: 10.1186/s12936-021-03885-x . 34446033 PMC8390081

[38] Amoah B, McCann RS, Kabaghe AN, Mburu M, Chipeta MG, Moraga P, et al. Identifying Plasmodium falciparum transmission patterns through parasite prevalence entomological inoculation rate. Elife. 2021;10:e65682. doi: 10.7554/eLife.65682 . 34672946 PMC8530514

[39] Malenga T, Kabaghe AN, Manda-Taylor L, Kadama A, McCann RS, Phiri KS, et al. Malaria control in rural Malawi: implementing peer health education for behaviour change. Global Health. 2017;13(1):84. doi: 10.1186/s12992-017-0309-6 . 29157284 PMC5694909

[40] Kaunda-Khangamwa BN, van den Berg H, McCann RS, Kabaghe A, Takken W, Phiri K, et al. The role of health animators in malaria control: a qualitative study of the health animator (HA) approach within the Majete malaria project (MMP) in Chikwawa District, Malawi. BMC Health Serv Res. 2019;19(1):478. doi: 10.1186/s12913-019-4320-x . 31299974 PMC6624973

[41] Nsona H, Mtimuni A, Daelmans B, Callaghan-Koru JA, Gilroy K, Mgalula L, et al. Scaling up integrated community case management of childhood illness: update from Malawi. Am J Trop Med Hyg. 2012;87:54–60. doi: 10.4269/ajtmh.2012.11-0759 . 23136278 PMC3748522

[42] Kok MC, Muula AS. Motivation and job satisfaction of health surveillance assistants in Mwanza, Malawi: an explorative study. Malawi Med J. 2013;25(1):5–11. . 23717748 PMC3653191

[43] Rutakumwa R, Mugisha JO, Bernays S, Kabunga E, Tumwekwase G, Mbonye M, et al. Conducting in-depth interviews with and without voice recorders: a comparative analysis. Qual Res. 2020;20(5):565–581. doi: 10.1177/1468794119884806 . 32903872 PMC7444018

[44] Tracy SJ. Qualitative research methods. Handbook of research methods in tourism: quantitative and qualitative approaches. Wiley-Blackwell, West Sussex, UK. 2013;10:9781781001295.

[45] Patton MQ. Qualitative evaluation and research methods. 2nd ed. Thousand Oaks, CA: Sage Publications;1990.

[46] Bandara W, Furtmueller E, Gorbacheva E, Miskon S, Beekhuyzen J. Achieving rigor in literature reviews: insights from qualitative data analysis and tool-support. Commun Assoc Inf Syst. 2015;37:154–204. 10.17705/1CAIS.03708.

[47] Gale NK, Heath G, Cameron E, Rashid S, Redwood S. Using the framework method for the analysis of qualitative data in multi-disciplinary health research. BMC Med Res Methodol. 2013;13:117. doi: 10.1186/1471-2288-13-117 . 24047204 PMC3848812

[48] Ingabire CM, Hakizimana E, Rulisa A, Kateera F, Van Den Borne B, Muvunyi CM, et al. Community-based biological control of malaria mosquitoes using Bacillus thuringiensis var. israelensis (Bti) in Rwanda: Community awareness, acceptance, and participation. Malar J. 2017;16(1):399. doi: 10.1186/s12936-017-2046-y . 28974204 PMC5627396

[49] Finda MF, Christofides N, Lezaun J, Tarimo B, Chaki P, Kelly AH, et al. Opinions of key stakeholders on alternative interventions for malaria control and elimination in Tanzania. Malar J. 2020;19(1):164. doi: 10.1186/s12936-020-03239-z . 32321534 PMC7178586

[50] Ng’ang’a PN, Mutunga J, Oliech G, Mutero CM. Community knowledge and perceptions on malaria prevention and house screening in Nyabondo, Western Kenya. BMC Public Health. 2019;19(1):423. doi: 10.1186/s12889-019-6723-3 . 31014321 PMC6480882

[51] Jatta E, Jawara M, Bradley J, Jeffries D, Kandeh B, Knudsen JB, et al. How house design affects malaria mosquito density, temperature, and relative humidity: an experimental study in rural Gambia. Lancet Planet Health. 2018;2:e498–e508. doi: 10.1016/S2542-5196(18)30234-1 . 30396441

[52] von Seidlein L, Ikonomidis K, Mshamu S, Nkya TE, Mukaka M, Pell C, et al. Affordable house designs to improve health in rural Africa: a field study from northeastern Tanzania. Lancet Planet Health. 2017;1(5):e188–e199. doi: 10.1016/S2542-5196(17)30078-5 . 29851640

[53] Knudsen JB, Pinder M, Jatta E, Jawara M, Yousuf MA, Søndergaard AT, et al. Measuring ventilation in different typologies of rural Gambian houses: a pilot experimental study. Malar J. 2020;19(1):273. doi: 10.1186/s12936-020-03327-0 . 32736629 PMC7393878

[54] Snetselaar J, Njiru BN, Gachie B, Owigo P, Andriessen R, Glunt K, et al. Eave tubes for malaria control in Africa: prototyping and evaluation against *Anopheles gambiae* s.s. and *Anopheles arabiensis* under semi-field conditions in western Kenya. Malar J. 2017;16(1):276. doi: 10.1186/s12936-017-1926-5 . 28778169 PMC5545004

[55] Sternberg ED, Cook J, Alou LPA, Assi SB, Koffi AA, Doudou DT, et al. Impact and cost-effectiveness of a lethal house lure against malaria transmission in central Côte d’Ivoire: a two-armed cluster-randomised controlled trial. Lancet. 2021;397(10276):805–815. doi: 10.1016/S0140-6736(21)00250-6 . 33640067 PMC7910282

[56] Pinder M, Bradley J, Jawara M, Affara M, Conteh L, Correa S, et al. Improved housing versus usual practice for additional protection against clinical malaria in The Gambia (RooPfs): a household-randomised controlled trial. Lancet Planet Health. 2021;5(4):e220–e229. doi: 10.1016/S2542-5196(21)00002-4 . 33838737 PMC8051018

[57] Larsen DA, Borrill L, Patel R, Fregosi L. Reported community-level indoor residual spray coverage from two-stage cluster surveys in sub-Saharan Africa. Malar J. 2017;16(1):249. doi: 10.1186/s12936-017-1893-x . 28610579 PMC5470197

[58] Munguambe K, Pool R, Montgomery C, Bavo C, Nhacolo A, Fiosse L, et al. What drives community adherence to indoor residual spraying (IRS) against malaria in Manhiça district, rural Mozambique: a qualitative study. Malar J. 2011;10:344. doi: 10.1186/1475-2875-10-344 . 22111698 PMC3339361

[59] Garfield R. Malaria control in Nicaragua: social and political influences on disease transmission and control activities. Lancet. 1999;354(9176):414–418. doi: 10.1016/S0140-6736(99)02226-6 . 10437886

[60] Garfield RM, Vermund SH. Health education and community participation in mass drug administration for malaria in Nicaragua. Soc Sci Med. 1986;22(8):869–877. doi: 10.1016/0277-9536(86)90241-8 . 3529425

[61] Abamecha F, Midaksa G, Sudhakar M, Abebe L, Kebede Y, Mamo A. Acceptability and feasibility of the school- engaged social and behaviour change communication approach on malaria prevention in Ethiopia: implications for engagement, empowerment, and retention (EER) of education sectors in malaria elimination effort. BMC Public Health. 2021;21(1):1909. doi: 10.1186/s12889-021-11995-z . 34674682 PMC8529361

[62] Purdey AF, Adhikari GB, Robinson SA, Cox PW. Participatory health development in rural Nepal: clarifying the process of community empowerment. Health Educ Q. 1994;21(3):329–343. doi: 10.1177/109019819402100305 . 8002357

[63] Atkinson JA, Vallely A, Fitzgerald L, Whittaker M, Tanner M. The architecture and effect of participation: a systematic review of community participation for communicable disease control and elimination. Implications for malaria elimination. Malar J. 2011;10:225. doi: 10.1186/1475-2875-10-225 . 21816085 PMC3171376

[64] Malenga T, Griffiths FE, van den Berg M, van den Berg H, van Vugt M, Phiri KS, et al. A qualitative exploration of the experiences of community health animation on malaria control in rural Malawi. Global Health. 2020;16(1):25. doi: 10.1186/s12992-020-00558-3 . 32197660 PMC7085180

[65] Walt G, Gilson L. Review article Reforming the health sector in developing countries: the central role of policy analysis. Health Policy Plan. 1994;9(4):353–370. doi: 10.1093/heapol/9.4.353 . 10139469

