# Supplementary figures and images for: Community-based house improvement for malaria control in southern Malawi: Stakeholder perceptions, experiences, and acceptability
Source: PLOS Glob Public Health. 2022 Jul 14;2(7):e0000627. doi: 10.1371/journal.pgph.0000627 (PMC10021647; doi:10.1371/journal.pgph.0000627)

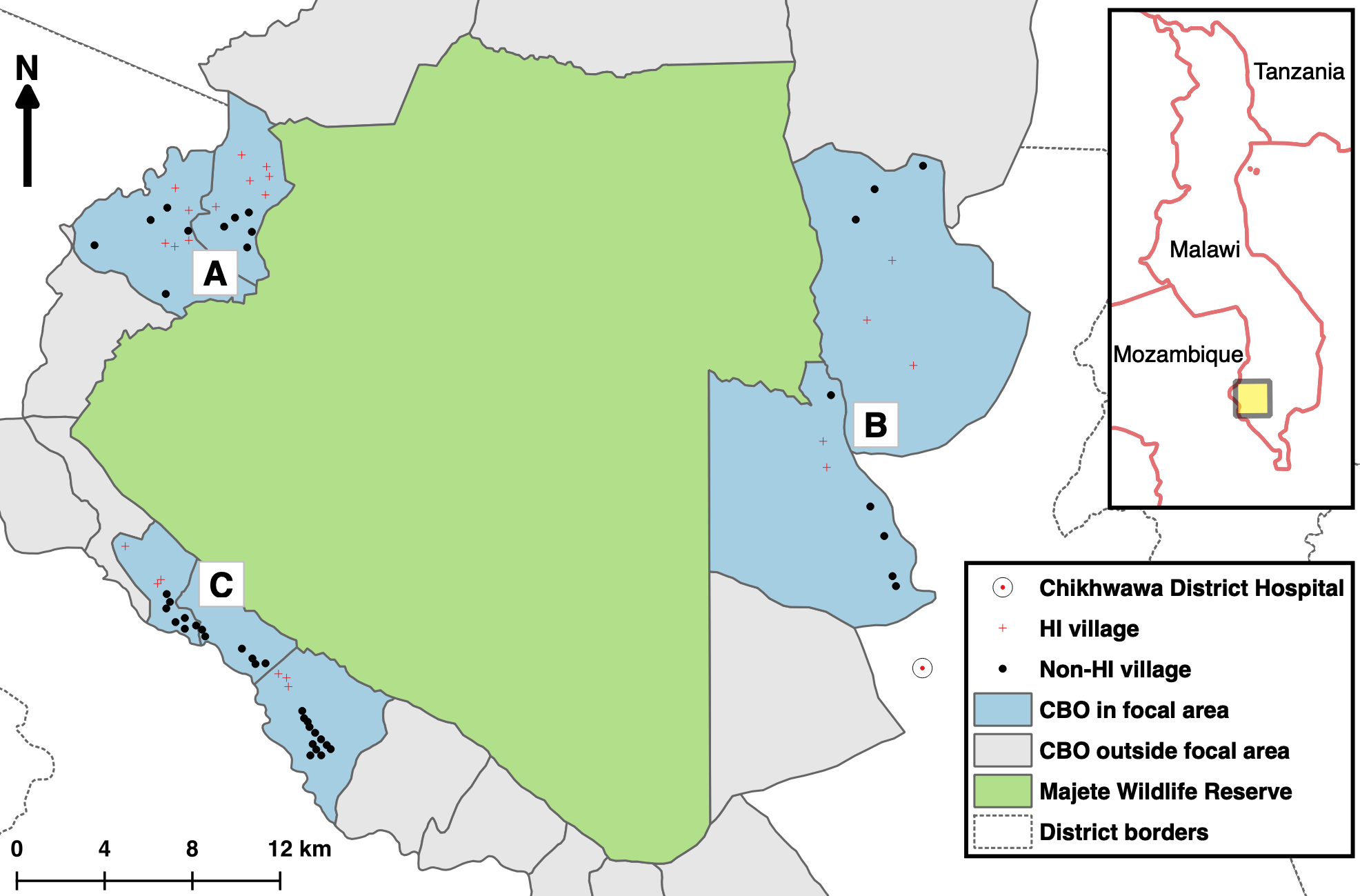

Supplement: S1 Fig — This map shows the study site and the villages where HI was implemented. (TIFF) [file pgph.0000627.s001.tiff]
