# Supplementary material for: Community-based house improvement for malaria control in southern Malawi: Stakeholder perceptions, experiences, and acceptability
Source: PLOS Glob Public Health. 2022 Jul 14;2(7):e0000627. doi: 10.1371/journal.pgph.0000627 (PMC10021647; doi:10.1371/journal.pgph.0000627)
Supplement: S1 Text — These qualitative interview guides show the questions that guided the interviews in this study. (DOCX) [file pgph.0000627.s002.docx]

**S1 Text. Interview guides**

**Appendix 1A: Focus Group Discussion with Health Animators**

**Topic Guide – Health Animators – English**

1. First, I would like everyone to introduce themselves.
2. I would like you to have a few minutes to think about your knowledge, experience and perceptions of House Improvement as a malaria control intervention, and finally your participation in the on-going HI activities in your village. Your participation is of paramount importance to the study. Is everyone ready to share their experience?

**Warm up: Roles of Health Animators**

1. How would you describe your experience working as a health animator in your village?
2. What is your impression of house improvement as a method for preventing malaria?
3. What do you understand your role to be?
4. Which rewards/benefits did you experience by being a Health animator?
5. What challenges did you encounter during your exercise as Health animators?

[**Probe**: May you please describe each challenge in detail]

1. How did you deal with each challenge?
2. How would you describe your moments of being an animator?

[**Probe**: What are the motivating factors of working as a health animator?]

[**Probe**: What are the demotivating factors of working as a health animator?]

**To determine Health animators’ knowledge, perceptions and acceptability of house improvement as a malaria prevention intervention**

1. Could you please describe what you know about House Improvement as a method of preventing malaria?

[**Probe**: What does house improvement involve? How does HI prevent malaria?]

1. How would you describe community perceptions of HI activity in your area?

[**Probe**: What are the comments and expectations generally made about the intervention?]

1. Do you think that house improvement has been accepted in your area as a malaria prevention intervention?

[**Probe**: If yes, please explain. If no, please explain]

1. Are there any negative concerns over House Improvement? (e.g. heat, light, ventilation, termites)

[**Probe**: If yes, what can be done in order to address such concerns?]

[**Probe**: Based on your experience, have the concerns influenced community motivation and participation in HI activities?] [**Probe**: How can HI be improved or made better?]

[**Probe**: Would other HI designs be more acceptable?]

1. How can the strategy of implementing HI be improved?
2. In your opinion, how does house improvement interact with indoor interventions like bed nets?

[**Probe**: Do people in HI houses feel that they no longer need to use bed nets?]

1. Why do you think people build houses with open eaves? Do they have any intended use?
2. Do you think that your community is now more aware about the risk of open eaves for malaria transmission?

[**Probe**: How do open eaves facilitate malaria transmission]

**Appendix 1B: Focus Group Discussion with HI Committee members**

**Topic Guide – HI Committee – English**

1. First, I would like everyone to introduce themselves.
2. I would like you to have a few minutes to think about your knowledge, experience and perceptions of House Improvement as a malaria control intervention, and finally your participation in the on-going HI activities in your village. Your participation is of paramount importance to the study. Is everyone ready to share their experience?

**Warm up: Roles of HI Committee Members**

1. How would you describe your experience working as a member of the HI committee?
2. What is your impression of house improvement as a method for preventing malaria?
3. What do you understand your role to be?
4. Which rewards/benefits did you experience by being a HI committee member?
5. What challenges did you encounter during your exercise as HI committee members? [**Probe**: May you please describe each challenge in detail]
6. How did you deal with each challenge?
7. How would you describe your moments of being in the HI committee?

[**Probe**: What are the motivating factors of working in the HI committee?]

[**Probe**: What are the demotivating factors of working in the HI committee?]

**To determine HI committee and knowledge, perceptions and acceptability of house improvement as a malaria prevention intervention**

1. Could you please describe what you know about House Improvement as a method of preventing malaria?

[**Probe**: What does house improvement involve? How does HI prevent malaria?]

1. How would you describe community perceptions of HI activity in your area?

[**Probe**: What are the comments and expectations generally made about the intervention?]

1. Do you think that house improvement has been accepted in your area as a malaria prevention intervention?

[**Probe**: If yes, please explain. If no, please explain]

1. Are there any negative concerns over House Improvement? (e.g. heat, light, ventilation, termites)

[**Probe**: If yes, what can be done in order to address such concerns?]

[**Probe**: Based on your experience, have the concerns influenced community motivation and participation in HI activities?] [**Probe**: How can HI be improved or made better?]

[**Probe**: Would other HI designs be more acceptable?]

1. How can the strategy of implementing HI be improved?
2. In your opinion, how does house improvement interact with indoor interventions like bed nets?

[**Probe**: Do people in HI houses feel that they no longer need to use bed nets?]

1. Why do you think people build houses with open eaves? Do they have any intended use?
2. Do you think that your community is now more aware about the risk of open eaves for malaria transmission?

[**Probe**: How do open eaves facilitate malaria transmission]

**Appendix 1C: Focus Group Discussion with community participants**

**Topic Guide – Community Participants – English**

1. First, I would like everyone to introduce him or herself.
2. I would like you to have a few minutes to think about your knowledge, experience and perceptions of House Improvement as a malaria control intervention, and finally your participation in the on-going HI activities in your village. Your participation is of paramount importance to the study. Is everyone ready to share their experience?

**Warm up: Community Experiences with implementing HI**

1. How would you describe your experience in implementing HI in your village?
2. What is your impression of house improvement as a method for preventing malaria?
3. What challenges do you encounter during your exercise of implementing HI?

[**Probe**: May you please describe each challenge in detail]

1. How do you deal with each challenge?
2. Do you have any reasons why people build houses with open eaves?

**To determine community’s knowledge, perceptions and acceptability of house improvement as a malaria prevention intervention**

1. Could you please describe what you know about House Improvement as a method of preventing malaria?

[**Probe**: What does house improvement involve? How does HI prevent malaria?]

1. How would you describe community perception of HI activity in your area?

[**Probe**: What are the comments and expectations generally made about the intervention?]

1. Do you think that house improvement has been accepted in your area as a malaria prevention intervention?

[**Probe**: If yes, please explain. If no, please explain]

1. Are there any negative concerns over House Improvement? (e.g. heat, light, ventilation, termites)

[**Probe**: If yes, what can be done in order to address such concerns?]

[**Probe**: Based on your experience, have the concerns influenced community motivation and participation in HI activities?] [**Probe**: How can HI be improved or made better?]

[**Probe**: Would other HI designs be more acceptable?]

1. How can the strategy of implementing HI be improved?
2. In your opinion, how does house improvement interact with indoor interventions like bed nets?

[**Probe**: Do people in HI houses feel that they no longer need to use bed nets?]

1. Do you think that your community is now more aware about the risk of open eaves for malaria transmission?

[**Probe**: How do open eaves facilitate malaria transmission]

**Appendix 1D: Topic Guide (IDI) Community Participants (Male and Female) – English**

**Topic Guide – IDI – English**

**Introductory Remarks**

1. First, I would like you to introduce yourself.
2. I would like you to have a few minutes to think about your knowledge, experience and perceptions of House Improvement as a malaria control intervention, and finally your participation in the on-going HI activities in your village. Your participation is of paramount importance to the study. Are you ready to share your experience?

**Warm up: Understanding your role and general principles of malaria**

1. Is malaria considered a serious health problem in this community?

[**Probe**: Why? Why not?]

1. What kind of things do people in this community usually do to protect themselves from malaria?
2. Do you know that your village is participating in malaria control through HI?
3. What is your impression of house improvement as a method for preventing malaria?
4. How would you describe your experience in implementing HI in your village?
5. In what ways have you engaged with the intervention?

[**Probe**: What are the motivating factors for participating in these activities?

What are the demotivating factors from participating in HI activities?]

1. What challenges do you encounter during your exercise of implementing HI?

[**Probe**: May you please describe each challenge in detail]

1. How do you deal with each challenge?
2. Do you have any reasons why people build houses with open eaves? Do eaves have any intended use?

**To determine community’s knowledge, perceptions and acceptability of house improvement as a malaria prevention intervention**

1. Could you please describe what you know about House Improvement as a method of preventing malaria?

[**Probe**: What does house improvement involve? How does HI prevent malaria?]

1. How would you describe community perceptions of HI activity in your area?

[**Probe**: What are the comments and expectations generally made about the intervention?]

1. Do you think that house improvement has been accepted in your area as a malaria prevention intervention?

[**Probe**: If yes, please explain. If no, please explain]

1. Are there any negative concerns over House Improvement?

[**Probe**: If yes, what can be done in order to address such concerns?]

[**Probe**: Based on your experience, have the concerns influenced community motivation and participation in HI activities?] [**Probe**: How can HI be improved or made better?]

[**Probe**: Would other HI designs be more acceptable?]

1. How does house improvement interact with indoor interventions like bed nets?
2. Do people in HI houses feel that they no longer need to use bed nets?
3. Do you think that your community is now more aware about the risk of open eaves for malaria transmission?

[**Probe**: How do open eaves facilitate malaria transmission]

**Appendix 1E: Topic Guide (KII) Key Informant Interviews (HSAs, Chiefs, from HI village)**

**Topic Guide – KII – English**

**Introductory Remarks**

1. First, I would like you to introduce yourself.
2. I would like you to have a few minutes to think about your knowledge, experience and perceptions of House Improvement as a malaria control intervention, and finally your participation in the on-going HI activities in your village. Your participation is of paramount importance to the study. Are you ready to share your experience?

**Warm up: Understanding your role and general principles of malaria**

1. Could you please describe what are considered serious health problems in this community?
2. Is malaria considered a serious health problem in this community?

[Probe: Why? Why not?]

1. What kind of things do people in this community usually do to protect themselves from malaria?
2. How do you understand your role of promoting health in this community to be?
3. Can you please elaborate how critical is your influence towards promoting health in this community?

**To determine the knowledge, perceptions and acceptability of house improvement as a malaria prevention intervention**

1. Could you please describe what you know about House Improvement as a method of preventing malaria?

[**Probe**: What does house improvement involve? How does HI prevent malaria?]

1. How would you describe community perception of HI activity in your area?

[**Probe**: What are the comments and expectations generally made about the intervention?]

1. Do you think that house improvement has been accepted in your area as a malaria prevention intervention?

[**Probe**: If yes, please explain. If no, please explain]

1. Are there any negative concerns over House Improvement? (e.g. heat, light, ventilation, termites)

[**Probe**: If yes, what can be done in order to address such concerns?]

[**Probe**: Based on your experience, have the concerns influenced community motivation and participation in HI activities?] [**Probe**: How can HI be improved or made better?]

[**Probe**: Would other HI designs be more acceptable?]

1. How can the strategy of implementing HI be improved?
2. In your opinion, how does house improvement interact with indoor interventions like bed nets?

[**Probe**: Do people in HI houses feel that they no longer need to use bed nets?]

1. Do you think that your community is now more aware about the risk of open eaves for malaria transmission?

[**Probe**: How do open eaves facilitate malaria transmission]

**Appendix 1F: In Depth Interview with HI Committee Dropout members**

**Topic Guide – IDI HI Committee Dropouts – English**

1. First, I would like everyone to introduce themselves.
2. I would like you to have a few minutes to think about your knowledge, experience and perceptions of House Improvement as a malaria control intervention, and finally your views in the on-going HI activities in your village. Your participation is of paramount importance to the study. Are you ready to share your experience?

**Warm up: Experience of your role as former HI Committee Members**

1. How would you describe your previous experience working as a member of the HI committee?
2. What did you understand your role to be? What challenges did you encounter when you participated in house improvement activities as HI committee member?

[**Probe**: May you please describe each challenge in detail]

1. How did you deal with each challenge?
2. Could you please explain why you decided to leave the HI committee?

[**Probe**: What were the demotivating factors of being in the committee?]

**To determine former HI committee dropout’s knowledge and perceptions of house improvement as a malaria prevention intervention**

1. Could you please describe what you know about House Improvement as a method of preventing malaria?

[**Probe**: What does house improvement involve?] How does HI prevent malaria?]

1. How would you describe the community’s perception of HI activity in your area?

[**Probe**: What are the comments and expectations generally made about the intervention?]

1. Are there any negative concerns over House Improvement? (e.g. heat, light, ventilation, termites)

[**Probe**: If yes, what can be done in order to address such concerns?]

[**Probe**: Based on your experience, have the concerns influenced community motivation and participation in HI activities?] [**Probe**: How can HI be improved or made better?]

[**Probe**: Would other HI designs be more acceptable?]

1. How can the strategy of implementing HI be improved?
2. In your opinion, how does house improvement interact with indoor interventions like bed nets?

[**Probe**: Do people in HI houses feel that they no longer need to use bed nets?]

1. Why do you think people build houses with open eaves? Do they have any intended use?
2. Do you think that your community is now more aware about the risk of open eaves for malaria transmission?

[**Probe**: How do open eaves facilitate malaria transmission]

1. Overall do you think that house improvement has been accepted in your area as a malaria prevention intervention?

[**Probe**: If yes, please explain. If no, please explain]

**Appendix 1G: Topic Guide (IDI) Community Non-Participants (Male and Female)**

**Topic Guide – IDI – English**

1. First, I would like everyone to introduce themselves.
2. I would like you to have a few minutes to think about your knowledge, experience and perceptions of House Improvement as a malaria control intervention. Your participation is of paramount importance to the study. Is everyone ready to share their experience?

**Warm up: Roles of the Community on HI**

1. Is malaria considered a serious health problem in this community?

[**Probe**: Why? Why not?]

1. What kind of things do people in this community usually do to protect themselves from malaria?
2. What is your impression of house improvement as a method for preventing malaria?

[**Probe**: What does house improvement involve?]

1. Do you have any reasons why people build houses with open eaves? Do eaves have any intended use?
2. Why did you decide not to participate in the on-going HI trial?

[**Probe**: What were the demotivating factors of participating in the trial?]

**To determine non-participants’ knowledge and perceptions of house improvement as a malaria prevention intervention**

1. How would you describe community perception of HI activity in your area?

[**Probe**: What are the comments and expectations generally made about the intervention?]

1. In your opinion, how does house improvement interact with indoor interventions like bed nets?

[**Probe**: Do people in HI houses feel that they no longer need to use bed nets?]
