# Supplementary material for: Community-based house improvement for malaria control in southern Malawi: Stakeholder perceptions, experiences, and acceptability
Source: PLOS Glob Public Health. 2022 Jul 14;2(7):e0000627. doi: 10.1371/journal.pgph.0000627 (PMC10021647; doi:10.1371/journal.pgph.0000627)
Supplement: S2 Text — This guided the coding process. (DOCX) [file pgph.0000627.s003.docx]

**S2 Text. Codebook**

NB: Multiple codes can be used to code the same statement from participants. Codebook organized to avoid forgetting the codes.

Key:

- Besides applying multiple codes, we should capture sufficient background information from the interviewer’s question.
  - If a participant’s response will be difficult to understand without the question, code the question with the response.
  - A quotation length should be long enough for readers to understand its meaning without context.
- Be careful that the right code is being applied to the right passage.
- Passages with participant response should not go uncoded.

| **NO** | **CODE** | **DEFINITION** | **INCLUSION** | **EXCLUSION** |
| --- | --- | --- | --- | --- |
|  | **HI Acceptability** | **Parent/ Main code:** Describes participant’s knowledge, perceptions and acceptability of House Improvement as one of the Malaria prevention intervention. This means that if a passage is coded with a child code, this parent code must also be applied. IF none of child code fits then ONLY the parent code applies | All participants views on how they have accepted HI as one of the malaria prevention intervention | All participants views related to other malaria prevention interventions other than HI |
|  | **HI acceptability** Knowledge and perception | Describes participant’s knowledge and perception of house improvement as one of the malaria prevention intervention in their area. |  |  |
|  | **HI acceptability:** Negative concerns | Describes participants views and narratives of people’s negative concerns about HI and how they can be addressed |  |  |
|  | **HI acceptability:** HI improvement | Describes participants views and opinions on how HI intervention can be improved |  |  |
|  | **HI acceptability:** HI interaction with Indoors intervention (ITNs) | Describes Participants views and opinions on how HI interact with other indoors malaria control strategies such as insecticide-treated bednets in their community |  |  |
|  | **HI acceptability:** Risk awareness | Describes participant’s views if people are aware of the risk of open eaves for malaria transmission? |  |  |
