# Supplementary material for: Community-based house improvement for malaria control in southern Malawi: Stakeholder perceptions, experiences, and acceptability
Source: PLOS Glob Public Health. 2022 Jul 14;2(7):e0000627. doi: 10.1371/journal.pgph.0000627 (PMC10021647; doi:10.1371/journal.pgph.0000627)
Supplement: S3 Text — Codes that were derived from the data using participants’ words. (DOCX) [file pgph.0000627.s004.docx]

**S3 Text. Inductive coding**

**Appendix 1: Inductive coding for Health Animator Focus Group Discussions (FGDs)**

**Abbreviations**

HA – Health Animator

HSA – Health Surveillance Assistant

FA-A – Focal Area A

FA-B – Focal Area B

FA-C – Focal Area C

FGD – Focus Group Discussion

IDI – In-depth Interview

KII – Key-informant Interview

Theme: Knowledge of HI

| Codes | Quotes |
| --- | --- |
| Closure of eaves | “…What I can explain on this, this, the mosquitos can no longer get inside the house because all open eaves are closed.” (P8 HA FA-A) |
| Closure of eaves | “…what I know is that, because we’ve got several lives: life of a child, life of an expectant mother, life of an elderly person, and life of a healthy person like me. We safeguard these lives by closing open eaves, so that these people are not severely affected.” (P2 HA FA-C) |
| Screening with wire gauze | “…house improvement is about closing open eaves and closing house windows with gauze wire.” (P5 HA FA-B) |
| Screening with wire gauze | “…the HI method is being a very useful and safety method. Why? We learnt that malaria transmitting mosquitoes climb the house walls to enter the house. So, if we properly close the house windows with gauze wire, then malaria transmitting mosquitoes won’t be able to enter the house.” (P3 HA FA-C) |
| Sealing Door spaces | “…It also involves checking whether the door is properly inserted for cutting down entry of mosquitoes into the house – so that only 10% of the mosquitoes enter the house should they at all manage to enter.” (P5 HA FA-B) |
| Sealing Door spaces | “…The other thing is that our doors have spaces that can let mosquitoes in. So we look for small pieces of timber and fit it in the spaces, so that mosquitoes have no entry into the house.” (P6 HA FA-B) |
| Closure of small openings | “…what I know [about house improvement] is that it’s about closing small openings, closing windows with gauze wire, and encouraging sleeping under a mosquito net.” (P3 HA FA-B) |
| Closure of small openings | “…sleeping under a mosquito net every night, closing our house windows, and sealing small openings that can let mosquitoes in.” (P2 HA FA-B) |
| Assembling bricks and mud | “…There is need to find bricks, water for mixing mud, then the work starts to close the house.” (P4 HA FA-B) |
| Assembling bricks and mud | “…We also encourage them, because many don’t close open eaves when building their houses because of running short of bricks. So, if there are say 5 people, we encourage them to mold and burn bricks together for closing open eaves, because sometimes they tell that “I don’t have bricks.” (P5 HA FA-B) |
| Malaria prevention | “...Closing open eaves is very useful. First, it reduces entry of mosquitoes into the house. That means those staying in the house are protected from malaria infection because there are no mosquitoes entering the house. Safety from malaria infection in turn means they can fulfil their development plans properly.” (P3 HA FA-C) |
| Malaria prevention | “…it helps in that if house windows are closed with gauze wire, small openings sealed, and people are encouraged to sleep under a mosquito net, that can reduce malaria. (P3 HA FA-B) |
| Malaria prevention | “…Now, in the villages where this project has been implemented, malaria seems to have reduced. That’s what gives us understanding that house improvement is one way of reducing malaria. (P5 HA FA-B) |
| Malaria prevention | “…I agree with what my friends have mentioned that malaria spreads through mosquitoes. By improving the houses, we are preventing mosquitoes from entering the houses. Entry of mosquitoes into the house happens at night, when we are asleep. So if open eaves are closed it means mosquitoes will have no chance of biting us. I therefore feel this method is useful for reducing malaria (P6 HA FA-C) |

Theme: Perceptions of the intervention

| Codes | Quotes |
| --- | --- |
| Happiness | “…This is very important program because people have learnt something. During the public meetings, they are able to express their views. Once they hear that there is a community meeting, they go in order to hear the problems that they are facing at the hospital like being shouted by the doctor, lack of medication and so they are happy because they are expressing their views freely.” (P2 HA FA-A) |
| Happiness | “…Talking of people’s comments, today I had 3 people who we’re asking me, “Did the project about closing open eaves finish?” I said, “No, we still have gauze wire and more is coming.” “My house window is broken.” I said, “I know. We are already in the process of counting houses with broken gauze wire.” And people are happy with the way we are inserting the gauze wire. (P2 HA FA-C) |
| Happiness | “…many people are happy with this project about closing open eaves because for example if someone’s house window has broken gauze wire, they persistently come to you, saying, “I will die sooner, mosquitoes are troubling me so much,” because when they insert gauze wire they are able to sleep. They feel sleeping under a mosquito net with open windows does not help because they are still exposed to mosquitoes when they get out of the net to visit the toilet.” (P5 HA FA-C) |
| Mindset change | “…People have welcomed this program very much because it has reduced malaria. People have changed their way of thinking because when they are building the house now, they are closing all open eaves to prevent mosquito from getting in the house. (P7 HA FA-A) |
| Applause | “…People have welcomed this program, when they are building the house, they close the open eaves that time. If they don’t get the gauze wire, they ask when we will receive the gauze wire. They have received well this program.” (P3 HA FA-A) |
| Positive | “…People are very positive about this issue. In the beginning, they felt it was not useful. But today they are appreciating it. Why? They are seeing a difference in the amount money they would spend previously and what they are spending now. Why? Because they are experiencing less cases of malaria infection in the house – because there are no mosquitoes entering their house. So today they are much interested in this method considering how much money they previously would spend on malaria. They are even saying that “If the project is delaying to give me the gauze wire I am going to purchase it on my own. Because the annual cost of taking a child to the hospital because of malaria is more than the cost of gauze wire.” I have been hearing these comments from people about gauze wire. (P3 HA FA-C) |
| Positive | “…people responded positively. When gauze wire was supplied, we started distributing it. But we would not just distribute the wire, we would also tell them what to do and how to do it to ensure intended results. (P7 HA FA-B) |
| Grateful | “…Adding on what my friends have been saying, people in the village, in their comments, are very grateful for what the Majete Malaria Project has been doing, providing gauze wire. They are very grateful. They are commenting that they are less mosquitoes entering their houses, and that they have been able to protect their lives. They are also saying that they have been able to reduce some of the problems that they face in the village.” (P1 HA FA-C) |
| Grateful | “…mmh, in our village, people had comments on the benefits of closing open eaves and sealing small openings, because there were now fewer mosquitoes in their houses than previously. So they would comment, saying, “Honestly, things are now better than before.” (P4 HA FA-B) |
| Informative/educative | “…This is a very important program because people have learnt something. During the public meetings, they are able to express their views. Once they hear that there is a community meeting, they attend in order to hear the problems that they are facing at the hospital like being shouted by the doctor, lack of medication and so they are happy because they are expressing their views freely.” (P2 HA FA-A) |
| Informative/educative | “…My village was one of the villages that was trailing behind. I find it unbelievable nowadays that people come and demand for wire gauze at my place, so I see this as something that is very pleasing. What people never expected to change in my village has now changed. So in my village people welcomed this development and are happy. (P1 HA FA-A) |
| Safety | “…In the past, there was too much mosquito breeding in our homes but after this program was introduced, it has helped to reduce the mosquito in the houses. In the past, when you get in the house, it was as if you have kept the pot of beer in that house but now, when you get in the house, you no longer have mosquito. If you get mosquito, it is their season but then you no longer have threats. People’s comments are that this program should continue, it should not just stop there.” (P8 HA FA-A) |
| Safety | “Because when the windows are closed with gauze wire even house flies, lizards, cockroaches, are prevented from entering the house. So, house improvement is one way of ensuring safety from different dangers.” (P1 HA FA-B) |
|  |  |
| Hesitant | “In the first year, people did not respond positively” (P6 HA FA-B) |
| Hesitant | “It’s just the same as others are alluding to, in the beginning, people were reluctant to accept house improvement” (P1 HA FA-B) |

Theme: Acceptability

| Codes | Quotes |
| --- | --- |
| Taking ownership | “…People welcomed this because malaria is now a history, they are able to save money after doing business. In the past, they were not able to keep money because they were going to the traditional healers when the child gets sick, but now when this program came, they welcomed it.” (P3 HA FA-A) |
| Taking ownership | “…In agreement with what P5 said, people reacted very positively to this project about house improvement. Why? Because they were in charge of improving their houses. Whenever we’re out in the villages, people from neighboring villages were asking, “When are you going to start in our village?” That means people have been positive about the project.” (P6 HA FA-C) |
| Community benefit | “…people are able to see the fruits of this program because in the past, they were often getting sick from malaria than now, people are now able to comply with the HI program.” (P5 HA FA-A) |
| Community benefit | “…Just commenting, people received this thing without problems. Why? Because people, seeing that gauze wire is delaying to come, have been closing their house windows using used mosquito nets. And when the gauze wire arrived they felt their houses would look the same as houses in town. That means malaria in our community will reduce. And people are grateful for the coming of the Majete Malaria Project; it’s helping them a lot. (P2 HA FA-C) |
| Community benefit | “…In our village, they received it because of the benefits of closing windows with gauze wire. Because house flies, cockroaches have no entry into their houses. Because of this many people have been very positive about closing windows with gauze wire and closing open eaves.” (P4 HA FA-B) |
| Demand of resources | “…People have been very positive about house improvement as one way of reducing malaria, because when we got reports [from households] about gauze wire wearing out and forwarded the reports to the office, if the office delayed to dispatch the gauze wire, people would come to us and complain, saying, “You are now putting us at the risk of malaria because you taught us about the danger of malaria, we have closed open eaves, but gauze wire is not being supplied.” (P5 HA FA-B) |
|  |  |

Theme: Challenges with House Improvement

| Codes | Quotes |
| --- | --- |
| Heat | “Yes people had complaints about house improvement because when constructing their houses they did not close open eaves. So, when they closed open eaves and windows with gauze wire after we had encouraged them, they started complaining, “The house is too hot, better when the windows had not been closed because there was ventilation.” (P7 HA FA-B) |
|  |  |
| Rusting | Also, a year after they had closed the windows, the gauze wire started rusting, and they were complaining that “Worn out gauze wire makes our house look dirty. (P7 HA FA-B) |
| Ventilation | That time people could not understand this, they were asking some questions like, ‘if you put gauze wire in the window, how are we going to breathe, you want us to die in the house? (P2 HA FA-A) |
| Termites | I experienced that challenge in my village where someone complained about closing open eaves leading to a problem of termites eating poles on the roof because the walls are now in contact with the roof. And when fixing the roof they have to remove the bricks they had closed open eaves with. As an animator, where there were lots of termites, I would provide them with chemicals that are used to spray cotton, so they can spray their house walls. (P1 HA FA-C) |
| Misconceptions | “People were talking a lot in the communities, they were referring us to Satanism. They were saying that the mosquitos that they are taking from our communities, they have their methods of sucking blood from us so that we should die. After we sensitized them, they started taking this method to be very important and not what they were thinking at the beginning.” (P8 HA FA-A) |
| Misconceptions | “Regarding challenges, because of lack of knowledge, some have been saying that inserting gauze wire on the window was locking the mosquitoes up in the house. So, through the education that we provide, we have been telling them that “No, if a mosquito ever enters the house, it doesn’t get out to transmit malaria somewhere else. You shouldn’t worry much about that. Also, mosquitoes have a short life span; if it’s locked up in the house, it will die shortly.” (P3 HA FA-C) |
| Community misconception on funding | “When the project was just starting, people felt the project had lots of money and were accusing us of being the ones pocketing the money: “You are taking the money for yourself – you should not put the gauze wire on my house window.” (P6 HA FA-C) |
| Community misconception on funding | “At the beginning, people were thinking that we want to get rich through them. They started receiving it through the community meetings that we were conducting, they are following it now” (P5 HA FA-A) |
| Traditional beliefs | “Complaints were there related to gauze wire. Some, because of religious beliefs, would refuse to close windows with gauze wire.” (P2 HA FA-B) |
| Lack of reporting | “Other challenges were that some would not report if the gauze wire came out of the window. This was challenge because after 6 months the problem of malaria would resurface.” (P6 HA FA-C) |
| Doubts | “When this program came in our community, people were doubting, they were asking, what we are going to eat once we go there or how much I am going to be paid when I go there. They started noticing that this program is benefiting them and it is not about getting money, they changed and they started using it properly” (P1 HA FA-A) |
| Availability of materials | “The other complaint people had about house improvement related to nails, which seemed to affect the progress of how many houses were closing windows with gauze wire. Because when we told them that they were to look for nails themselves, they would say, “Better if you were providing nails too, then we would close the windows right away.” (P5 HA FA-B) |
| Staying with Pets | “My chicken is having problems laying eggs because the gauze wire is stopping it from entering the house.” So we teach them other methods of letting the chicken in to lay eggs. These are the challenges that people have been reporting” (P3 HA FA-C) |
| Training problems | “The other thing is that when the project had expanded to all the villages some animators were not invited for training, and were complaining to say, “How come we have been left?” (P6 HA FA-C) |

Theme: Solutions to HI challenges

| Codes | Quotes |
| --- | --- |
| Sensitization | ”But through sensitization, with support from EPO, health surveillance assistants and animators from other villages, people understood what was the project was about.” (P6 HA FA-C) |
| Sensitization | “After we sensitized them, they started taking this method to be very important and not what they were thinking at the beginning.” (P8 FA FA-A) |
| Education | “They started receiving it through the community meetings that we were conducting, they are following it now.” (P5 HA FA-A) |
| Education | “We then started teaching them that this gauze wire has small holes but it prevents mosquito from getting inside the house. They started understanding it little by little and now, everything is okay.” (P2 HA FA-A) |
| Education | “What we have done is for reducing entry of mosquitoes into your house, because if we don’t give you gauze wire and leave the windows open, then we have not protected you. We are giving you gauze wire so you can close your windows, not to make your house look good, but to prevent mosquitoes from coming into your house, so malaria can be reduced or stopped completely.” (P7 HA FA-B) |
| Resource Provision | “If the office could also provide nails beside gauze wire, that would be good.” (P5 HA FA-B) |
| Resource Provision | “Worn out gauze wire makes our house look dirty.” When reported this to the office, the office supplied new gauze wire and people replaced the one that had worn out, and they were commending the new gauze wire because it was durable.” (P7 HA FA-B) |
| Motivation through trainings | “um, for the complaint from committee members, something like refresher courses would motivate them to work harder.” (P2 HA FA-C) |
| Motivation through trainings | “So, it’s just a request that whenever there’s an activity, there should be fairness in who attends that activity, not some going others staying behind. It’s painful because those that went end up mocking those that did not go as though those that did not go personally chose not to go. So, for the success of the project, there should be fairness. If there’s training, all should access it.” (P3 HA FA-C) |
| Community leaders intervention |  |

Theme: Demotivating ways of challenges to the community

| Codes | Quotes |
| --- | --- |
| Discouragement | “There are others who just keep the gauze wire after receiving it because of lack of nails, so this poses a discouragement to others who have fixed their gauze wire on the windows even though both of them received the gauze wire on the same day.” (P7 HA FA-B) |
| Discouragement | “It seems some people wanted to be discouraging their friends however when we trained the communities, they were able to discuss amongst themselves and say, brother or sister, what you are saying is not true, those people want to protect our lives from malaria because some developments are not successful because of issues of malaria.” (P3 HA FA-B) |
| Anger | “Some women at the water points were discussing that once the program reached their homes, they were going to chase people away.” (P8 HA FA-A) |

Possible accepted HI designs

| Codes | Quotes |
| --- | --- |
| Using nets instead of gauze | “If gauze wire is not available, we can close windows using used mosquito nets or sacks, and mosquitoes will be prevented from entering the house (P1 HA FA-B) |
| Using nets instead of gauze | “Depending upon the economic situation of an individual, if gauze wire is not available, a used mosquito net would be an alternative because gauze wire and a mosquito net have a similar netting.” (P3 HA FA-C) |
| Using nets instead of gauze | “This method is very helpful because if we put up the gauze wire there’s ventilation in the house, whereas other methods of closing the window might result in no air entering the house. If gauze wire is expensive, we can use a mosquito net.” (P6 HA FA-C) |
| Plastering with sand or cement on walls | “Regarding closure of open eaves or small openings, if you can’t close them with mud, and if you can look for sand, you can close them by plastering the walls.” (P1 HA FA-B) |
|  | “But another way of closing openings or closing windows that we could teach to people is mixing sand with mud, we women call it ‘kutsutsuta’. That could be an alternative method.” (P6 HA FA-B) |

Theme: Promoting HI implementing strategy

| Codes | Quotes |
| --- | --- |
| Conducting Animator village meetings | “House improvement could be promoted through us animators holding meetings, where we can teach people how they can improve their houses, so that they may be motivated for the work.” (P2 HA FA-B) |
| Conducting Animator village meetings | “For the house improvement procedures to be improved, um, as an animator, holding community meetings is enough.” (P6 HA FA-B) |
| Supervisory visits (Spot checks) by the project (MMP) | “Also if the MMP (Majete malaria project) team could have village visits once every 3 or 6 months and speak about the project so that people understand. That will also make people respect [animators] to say, “This person is sent by an office,” because there are some that despise you” (P6 HA FA-C) |
| Supervisory visits (Spot checks) by the project (MMP) | “When we went for the training, we’re promised that they would be coming to monitor how the work was being done. But there has been no other monitoring visit since the initial visit and people have been asking: “Why are they not coming to visit us anymore? Did the project finish? Or they are just dealing with you now, not interested in us anymore?” So, as a matter of request, committee members should be visited for the continuation of the work.” (PF HA FA-C) |
| Cooperation among service providers | “The other approach we have used previously is forming a team of say 4 animators and holding a village meeting together, especially if an animator had problems addressing people in a particular village.” (P6 HA FA-C) |
| Cooperation among service providers | “We should continue to work together with the committees, chiefs as already said by my friend here, we need to progress together because this is our development.” (P3 HA FA-A) |
| Cooperation among service providers | “Further, since a project works on timelines, and given that in a village there are some who can’t close open eaves on their own such as the physically disabled, or the elderly, or orphaned kids, we and the committee members together would volunteer to say, “Tomorrow let’s go and work on such and such houses.” (P6 HA FA-C) |
| Using model houses | “To ensure uptake of the project, we use houses that are along the roads as demonstration houses, so that everyone passing by can see and ask: “Whose house is this?! How are they doing this?! Where are they getting these things from?!” So, we tell them the organization that provides those things. We explain to them how it’s done, and that the project is currently implemented in one village as a trial and will later roll out to other villages.” (P2 HA FA-C) |
| Showing Commitment | “I am thinking that if we the villagers decide together to stop malaria, then this work can be promoted. Without relying on the office, if we the beneficiaries personally show much interest, and because of experiences of benefits of house improvement by households that already implemented it, then this work can be promoted.” (P4 HA FA-B) |
| Showing Commitment | “House improvement can be promoted if people do what we teach them.” (P7 HA FA-B) |
|  |  |

Theme: Relationship of HI and mosquito nets

| Codes | Quotes |
| --- | --- |
| Indoor Protection | “There is a relationship, if we do house improvement, and there is a hole somehow in the house wall and a mosquito has access to entry, the mosquito net forms a barrier for protection to this mosquito.” (P6 HA FA-A) |
| Indoor Protection | There’s a connection between house improvement and using a mosquito net because if someone closes their house windows, should mosquitoes find entry into the house through the door, the person won’t be bitten because they are covered by net.” (P6 HA FA-B) |
| Indoor Protection | “I feel the connection between the house improvement method and the method of using a mosquito is that you need to use a mosquito even when the house has been improved in case mosquitoes manage to come into the house.” (P1 HA FA-C) |
| Alternative malaria control method | “Conquering with what my friends have said, there’s a relationship. Because we can’t reduce malaria by house improvement alone. Why? Because out of 100 mosquitoes 10 might find entry into the house. Therefore, if you are sleeping under a mosquito, the 10 mosquitoes won’t bite you. Then we have prevented malaria.” (P6 HA FA-C) |
|  |  |

Theme: Use of Mosquito nets in presence of HI

| Codes | Quotes |
| --- | --- |
| Useful | “I feel people still find sleeping under a mosquito net useful despite their houses having been improved. How do I know this? I usually have meetings with the people, and during the meetings I teach them about the benefit of closing open eaves and the benefit of sleeping under a mosquito even when the open eaves have been closed. The other thing is that when we are making household visits we ask them to show us where they sleep – we find that most of them have a mosquito net set up for use. So yes people feel it’s useful to use a mosquito net.” (P6 HA FA-C) |
| Useful | “After implementing house improvement, people still find using a mosquito useful because of what we taught them. We taught them that house improvement and using a mosquito go together. If you implement house improvement but don’t use a mosquito net, should mosquitoes find entry into the house through the door, then you are going to be bitten by this malaria-transmitting mosquito.” (P2 HA FA-B) |
| Important | “Through the lessons that we give them, people feel it’s very important to sleep under a mosquito because we teach them that [closing open eaves] only keeps away 90% of the mosquitoes, but 10% of them may still find entry into the house and cause malaria. So, we advise them that they sleep under a mosquito net for a complete malaria prevention” (P3 HA FA-C) |
| Varying mosquito entry points | “If you implement house improvement, you still need to sleep under a mosquito net because mosquitoes have different entries into the house. The gauze wire might have a small hole and mosquitoes might come in through that. So, if you are not using a mosquito, the mosquitoes are going to bite you, and if the mosquitoes that bite are those responsible for malaria transmission, then you will have malaria. So we encourage them to sleep under a mosquito net all the time and all year round even when they have closed open eaves and windows with gauze wire.” (P7 HA FA-B) |
| Varying mosquito entry points | “It is necessary because the first method was to improve house by closing the open eaves and putting of gauze wire in the windows but we use the door when getting in and out of the house, the mosquito gets in the house and then it finds you sleeping under the net. I believe these methods are similar and we should use both.” (P5 HA FA-A) |
|  |  |

Theme: Animators’ knowledge and view on having open eaves

| Codes | Quotes |
| --- | --- |
| Easy entry to mosquitoes | “We could say that today most people understand that it’s a huge difference between a house with unclosed open eaves and a house with closed open eaves in terms of entry of mosquitoes into the house. A house with unclosed open eaves lets in more mosquitoes than does one with properly closed open eaves.” (P4 HA FA-B) |
| Easy entry to mosquitoes | “They have realized because once they don’t close the eaves, mosquito entry in the house is easy but if you close open eaves and sleep under the net, you don’t easily get mosquito bites |
| Regular malaria transmission | “People in my village have completely understood that. Because we would give them examples and they have personally seen differences – households with open eaves unclosed were experiencing regular malaria infection. (P4 HA FA-B) |
| Regular malaria transmission | “Yes people now understand that leaving eaves unclosed contributes to the spread of malaria because lots of mosquitoes enter the house through the open eaves. But if you close eaves, there are few mosquitoes entering the house, they enter through the door when you open it and move out. But if you sleep under a mosquito net, mosquitoes won’t bite you.” (P1 HA FA-B) |
| Regular malaria transmission | “House improvement is good because people that we encouraged to improve their houses are now commending. Before house improvement, sickness with malaria never freed their homes. But today they are testifying that malaria has reduced because of what taught them to do.” (P7 HA FA-B) |
|  |  |

**Appendix 2: Inductive coding for Health Surveillance Assistants (HSAs) IDIs**

Theme: Knowledge towards HI

| Codes | Quotes |
| --- | --- |
| Closure of eaves | “I know that when the Majete Malaria Project started their work, they were asking people in their impact area to be closing the eaves of their houses as well as spaces in the windows and the door through which mosquitoes can enter into the house. People are asked to either the do the work on their own or in conjunction with the village committee.” (HSA FA-A) |
| Closure of eaves | “There were volunteers that were selected to take the lead role and these are called health animators. These animators provide education to the community on how to do house improvement, in newly built houses and old houses on how they can close the eaves. They encourage people to close the eaves in their houses so that malaria can be prevented.” (HSA FA-B) |
| Closure of eaves | “I know couple of things about this method. In House Improvement, they close the windows through which mosquitoes can enter into the house when they are open during the day or at night. The closing of windows and all other spaces through which mosquitoes can enter the houses, it reduces the number of mosquitoes entering the house. This, together with use of mosquito nets, results in reduction in the percentage of people suffering from malaria because they do not get mosquito bites.” (HSA FA-C) |
| Fixing gauze wire | “As I have pointed out that many people want to have their houses improved with the gauze wire, they mainly want this to be done so that they prevent malaria.” |
|  |  |
| Malaria prevention | “In the same way, when mosquitoes get the smell of a person in the house, they enter into the house through the open eaves. Therefore, when the house is improved, mosquitoes fail to enter into the house and remain outside. This means only few mosquitoes, if any, will get into the house compared to an unimproved house” (HSA FA-A) |
|  | “On House Improvement, people say they can see the difference with the time they had not improved their houses that there used to be a lot of mosquitoes in the house. Many people say they get mosquito bites when they are outdoors more than when they are indoors which is a change from how it was previously.” (HSA FA-B) |

Theme: Perceptions of HI

| Codes | Quotes | Theme |
| --- | --- | --- |
| Hectic work | “The work seems to be tedious to some people in their own thinking.” (HSA- FA-A) |  |
| Lacking materials | “At first it was difficult to tell people to do house improvement because they would build a house without closing the eaves and a majority complained of lacking bricks which was difficult for them to build houses.” (HSA- FA-A) |  |

Theme: Acceptability of HI

| Codes | Quotes |
| --- | --- |
| Community benefit | “However, to those that know the dangers of malaria as well as the importance of House Improvement to protect themselves, they accept the work.” (HSA- FA-A)  “Yes. As I pointed out at first that others accepted it. We can say about 60% of the people accepted the work.” (HSA- FA-A) |
| Reduction in malaria | “People accepted this method very well. This is because there are so many methods that people were being taught how each one prevents malaria. Many of our houses have open windows or have spaces in the walls to promote ventilation. So, people think this work will be helpful because a lot of mosquitoes enter into the houses in the evening.” (HSA- FA-C) |
| Community benefit | “However when this issue of HI was introduced, and after the community was told about the advantages of having HI and closing eaves, little by little people began to accept it and they saw it as a good thing. So has being found out that apart from the heat we have here in Chikhwawa, people have accepted HI with the fact that they will prevent malaria.” (HSA- FA-C) |
| Reduction in malaria | “People are very happy because the way they used to stay back then without closing eaves with now when they started closing the eaves, people expressed happiness because firstly, malaria cases are minimal, people are less sick.” (HSA- FA-B) |
| Low mosquito presence | “On House Improvement, people say they can see the difference with the time they had not improved their houses that there used to be a lot of mosquitoes in the house. Many people say they get mosquito bites when they are outdoors more than when they are indoors which is a change from how it was previously.” (HSA- FA-B) |
| Economic/monetary savings | “If they were sick they would be going to Kapichira clinic and pay for the health services but right now this has actually reduced. Very few cases are going there at the clinic. So this money that is saved by not going to the clinic is now being used for other household developments. People are indeed very happy about the introduction of this intervention in the villages because it has helped their households to develop.” |
|  |  |

Theme: Challenges with House Improvement

| Codes | Quotes |
| --- | --- |
| Poorly designed houses | “The main issue is about the designs of their houses. Some houses were built in a way that they pose problems when tacking the gauze wire because they never thought in the first place that they would tack gauze wire in their houses.” (HSA-FA-C) |
| Rusting | “Yes, complaints indeed will always occur, for instance at first the gauze wire which we received for the windows underwent rusting so people complained that the wire was going bad and was rusting.” (HSA-FA-B) |
| Lacking of materials | “Apart from that people that had newly built houses and those in old houses who wanted to build new houses when they told the HI committee that they were looking for wire gauze, they received a response that wire gauze is no longer in stock.” (HSA-FA-B) |
| Lacking of materials | “It only sometimes a burden for them to fetch the required materials to improve the house in which they have been living for years.” (HSA-FA-A) |
| Heat | “Another thing was that here in Chikhwawa it is very hot so many people took it as a style of building a house with open eaves as a way of minimizing this heat.” (HSA-FA-B) |

Theme: Solutions to HI challenges

| Codes | Quotes |
| --- | --- |
| Training/Capacity building | “However, everyone wants the gauze wire. People would be very happy if they were given enough expertise so that they have their houses improved.” (HSA-FA-C) |
| Communication | “So like for us, we normally communicate with the health animators, because they are the ones who are looking after this job and are residing in the villages, of course there is also the HI committee. So we normally conduct a village meeting together with the animators and the HI committee so that they can hear the people’s grievances. Whatever is being discussed at those meetings are taken by the animators and we ask them to deliver to the people responsible from MMP in order to get a right response.” (HSA-FA-B) |

Theme: Demotivating ways of challenges to the community

| Codes | Quotes |
| --- | --- |
| Lack of materials | “It can be improved if the materials are available such as wire gauze, sometimes we have new houses that have been built but there is no wire gauze. This demotivates the people because they always hear that they should improve their houses and yet the materials are not available. But if wire gauze is readily available, people will be eager to build houses so that they receive the wire.” (HSA-FA-B) |

Theme: Promoting HI implementing strategy

| Codes | Quotes |
| --- | --- |
| Coordination | “These methods can be promoted by coordination especially between us as Health Surveillance Assistants working at the hospital and our friends doing the job. This is because there are sometimes communication problems such that the work might be done without us knowing.” (HSA-FA-C) |
| Taking responsibility | “However, I would love that the owner of the house should take full responsibility. When the measurements are taken, the owner of should also be taught the importance of House Improvement rather than being told that he or she should keep the gauze wire and that the committee members will come to help them with the work.” (HSA-FA-A) |
| Presence of materials | “It can be improved if the materials are available such as wire gauze, sometimes we have new houses that have been built but there is no wire gauze.”(HSA-FA-B) |

Theme: Relationship of HI and mosquito nets

| Codes | Quotes |
| --- | --- |
| Double protection | “Yes, it is there. Because the time people are doing house improvement we also encourage them to sleep under a mosquito net every time and throughout the year.” (HSA FA-B) |
| Double protection | “Yes, there is a very strong relationship. This is because when people are using mosquito nets only, they get mosquito bites when outside the house or while chatting at the living room before going to bed. However, when we close the windows and spaces, there is few or no mosquitoes inside the house thereby preventing mosquito bites while chatting before going to bed. Therefore, when they sleep under mosquito nets besides tacking gauze wire in windows and spaces, there is an increased chance of preventing malaria than using mosquito nets only.” (HSA-FA-C) |
| Double protection | “Yes, there is a relationship because if the mosquitoes have failed to enter into the house, they will not bite you. When you sleep under an insecticide treated net, you will not get bitten by mosquitoes that have entered the house either.” (HSA-FA-A) |

Theme: Use of Mosquito nets in presence of HI

| Codes | Quotes |
| --- | --- |
|  | “Many people did not realize this at first. However, with time of teaching, some people have understood that it is necessary that the houses should be improved so that their households are protected from malaria.” (HSA-FA-C) |
| Challenge | “I can say that this can be a challenge. That is the reason I at first said it is surprising that the cases of malaria are increasing. I think when people improve their houses, they think using mosquito net in not necessary. Many of them think that because they have improved their houses and they do not hear any mosquitoes boozing, there is no need to use mosquito nets.” (HSA-FA-A) |
|  | “Yes, because of the information we give them they sleep under the mosquito net. Apart from them having an improved house, we also receive nets and we distribute them to the community. This helps them to question the significance of having the mosquito net with them. They would have that encouragement that if I have improved my house I should also be sleeping in a mosquito net.” (HSA-FA-B) |

Theme: Alternatives to HI

| Codes | Quotes |
| --- | --- |
| Bed nets | “I did point out earlier on that the other method is the use of mosquito nets.” (HSA FA-C) |
| Clearing stagnant water | “Additionally, there is need to clear tall grass and stagnant water around homes to prevent mosquitoes.” (HSA FA-C) |
| Bed nets | “Apart from this what we tell them is that they should sleep in mosquito nets.” (HSA FA-B) |

Theme: Knowledge on significance of Eave closure

| Codes | Quotes |
| --- | --- |
| Realize | “Yes. Many people are realising that when you leave the spaces open, a lot of mosquitoes enter into the house.” (HSA FA-A) |
| Not all realize this | “In concluding on this issue, not all the people do know this. However, some of the people who are able to get close to the people who were doing the work do know. The main issue is that there is need to continue teaching them so that they realize that closing the eaves can help prevent mosquitoes from entering their houses.” (HSA FA-C) |
| Realize | “A lot. We have educated them that when they are leaving open eaves they are giving chance for malaria mosquitoes to enter the time they are sleeping at night but when they close the eaves, mosquitoes do not have the chance of house entry. We tell them this because as you may know Chikwawa is very hot so this issue of closing eaves always comes up by the community as it is a hot place and they would complain. We have managed to convince them and they no longer complain about feeling hot inside.” (HSA FA-C) |
