# Supplementary material for: Community-based house improvement for malaria control in southern Malawi: Stakeholder perceptions, experiences, and acceptability
Source: PLOS Glob Public Health. 2022 Jul 14;2(7):e0000627. doi: 10.1371/journal.pgph.0000627 (PMC10021647; doi:10.1371/journal.pgph.0000627)
Supplement: S4 Text — Codes that were derived from the question (interview) guide. (DOCX) [file pgph.0000627.s005.docx]

**S4 Text. Deductive Coding**

**Description:** This file contains quotes describing participants’ knowledge, perceptions, and acceptability of House Improvement as one of the strategies for malaria prevention. These were quotes obtained from participants in the Focus group discussion, in-depth interviews, and key-informant interviews

**Abbreviations**

FGD – Focus Group Discussion

IDI – In-depth Interview

KII – Key-informant Interview

<Files\\Chief Kuzambo - KII> - § 9 references coded [20.08% Coverage]

Reference 1 - 2.38% Coverage

Could you please describe what you know about House Improvement as a method of preventing malaria?

# Chief: If we can be lucky again to be given gauze wires and cover in our homes and closing the door, but also sleeping under a mosquito net, it can help us a lot

Reference 2 - 0.70% Coverage

# Chief: People welcomed it this year only that some are lazy to do the work

Reference 3 - 4.00% Coverage

Are there any negative concerns over House Improvement? (e.g. heat, light, ventilation, termites)

[**Probe**: How can HI be improved or made better?]

[**Probe**: Would other HI designs be more acceptable?]

# Chief: Committee which meets with the people and mostly they find some who are not taking part and talk bad about the project. But its the duty of us chiefs to call this people and help them understand so that they also should take part.

Reference 4 - 2.67% Coverage

: If yes, what can be done in order to address such concerns?]

# Chief: If the organisation can help us and start giving us wire gauze once again so that we should start again improving houses, us as chiefs are going to take part and encourage all those discouraged so that the way should go on

Reference 5 - 2.72% Coverage

# Chief: If the committe is complaining like that we feel like they start getting discouraged, and when they get discouraged and tell us, we go and strongly speak to the person, we know that they have failed and getting discouraged so we take part as parents and correct the source of the discouragement

Reference 6 - 1.57% Coverage

# Chief: Only using mosquito nets without improving the house cannot help that much. But if we add to house improvement sleeping under a mosquito net we can save our health

Reference 7 - 3.08% Coverage

# Chief: Yes they are supposed to, because when getting out to pass water, we don’t close the door, we only close when we go back to sleep. When we are outside mosquiotoes enter and we close the door with the mosquitoes inside. So sometimes the children can take out the nets while sleeping. So the mosquitoes can enter and transmit malaria

Reference 8 - 1.63% Coverage

# Chief: People have known because when we were enlightened we thought that improving the house by closing windows and eaves, we saw that is was important and a lot of us took part.

Reference 9 - 1.32% Coverage

# Chief: Don’t get tired with them. Visit them, teach them and enlighten them on the importance of house improvement using the materials provided.

<Files\\Chief Maganga 2 Chichewa KII> - § 8 references coded [12.08% Coverage]

Reference 1 - 1.80% Coverage

# When a house is built, it possesses some problems that makes mosquito entry to be easy. So the MM project brought ways of closing eaves, and windows with wire, which restricts the entry of mosquitoes.

Reference 2 - 0.74% Coverage

# Many people say the work is good and is helping them, they can see the goodness.

Reference 3 - 0.83% Coverage

# Yes they received it. Thats why many of them in the village accepted to close their houses.

Reference 4 - 1.60% Coverage

# At first they were complaining that the wire that was being give would easily rust. But they wire that is being received now seems to be good, because its serving for a long time.

Reference 5 - 2.36% Coverage

# In the villages there are committees. If one has a problem, for example, his/her house is not in good condition, he/she goes to those people, where theu give their report and get results. So if their wire is not in good condition, the committee brings new wire.

Reference 6 - 0.96% Coverage

# These complaints are encouraging the people to work so that they eliminate the malaria problem they have.

Reference 7 - 2.78% Coverage

# Respondent: At first they thought that after improving their house there was no need of sleeping under a net because no mosquito would enter again. But after teaching them that mosquitoes could still enter the house while they are chatting at night, they saw that it was important to still sleep under a net.

Reference 8 - 1.00% Coverage

# Respondent: People have known because it is one of the ways that allows entry and exit of mosquitoes in a house.

<Files\\Community participant chambo IDI> - § 7 references coded [25.05% Coverage]

Reference 1 - 1.75% Coverage

yes it is important

**INTERVIEWER**: explain

**PARTICIPANT:** because since we did house improvement there is no any single person suffering from malaria than before

Reference 2 - 1.80% Coverage

thank you. I want to know, how do people perceive house improvement intervention?

**PARTICIPANT:** it is productive to us since we are no longer suffering from malaria.

Reference 3 - 4.06% Coverage

thank you so much. What are comments and expectations people made about the project?

**PARTICIPANT:** they are saying these wires are productive to us since we are not frequently suffering from malaria, had it been there was no wire we would have been suffering malaria as before. Now we are able to work our personal things and our families are healthy in terms of malaria.

Reference 4 - 5.96% Coverage

thank you so much. Do you think that house improvement has been accepted in your community as one way of preventing malaria?

**PARTICIPANT:** yes

**INTERVIEWER:** could you explain

**PARTICIPANT:** Chambo village accepted this project, once we heard about putting gauze wire we said we will do it, and now people are continuing doing it without any sort of rudeness; because now we can compare our lives than before. Once they are been told to do some things people are very fast doing it to avoid suffering from malaria and giving pressure to others

Reference 5 - 5.33% Coverage

thank you let us proceed to other section. What are the negative concerns people say towards House improvement?

**PARTICIPANT:** no, there is no problem there

**INTERVIEWER:** what are the concerns that are motivating or demotivating house improvement?

**PARTICIPANT:** the concerns are like; during rainy season these wire develop rust hence mosquitoes sometimes enter, but still we are just keeping on using mosquito net because some house do not build good veranda hence wires develop rust

Reference 6 - 2.68% Coverage

thank you, how do you think house improvement standards can be improved?

**PARTICIPANT:** encouraging the ones who brought the idea of house improvement and tell them to keep on giving us the wire and other things if they have to prevent malaria

Reference 7 - 3.47% Coverage

do you think people are aware that open eave facilitate malaria transmission?

**PARTICIPANT:** yes, they are aware

**INTERVIEWER:** how do open eaves facilitate malaria transmission?

**PARTICIPANT**: leaving open eaves let the mosquitoes to enter inside and bite you if not slept in the mosquito nets, hence malaria starts

<Files\\Community participant Chaziya IDI> - § 4 references coded [14.85% Coverage]

Reference 1 - 6.86% Coverage

**INTERVIEWER**: let us go to another section. I want to learn from you, can you explain what you know about house improvement as one way of preventing malaria? (Repeating malaria)

**PARTICIPANT:** one way which I know is that once closing the eaves mosquitoes cannot enter in large numbers

**INTERVIEWER:** could you explain what it involves in house improvement activities? As they are improving the house what activities happen there?

**PARTICIPANT:** come again

**INTERVIEWER:** what does it involve in house improvement, what activities happen during course of action?

**PARTICIPANT:** hmm have failed

**INTERVIEWER**: okay. How does HI been perceived by your village?

**PARTICIPANT:** they accepted this calmly since the disease is been reduced, mosquitoes have reduced than years before this organisation

Reference 2 - 1.90% Coverage

**INTERVIEWER:** do you think this House improvement intervention is accepted as one way of preventing malaria?

**PARTICIPANT:** they accepted this, when you close the eaves mosquito enters is small number than being open.

Reference 3 - 1.96% Coverage

**INTERVIEWER:** I want to hear your views, is there any relationship between house improvement and using mosquito nets? (Repeating the question)

**PARTICIPANT**: there link which is there is that both are ways of preventing malaria

Reference 4 - 4.13% Coverage

**PARTICIPANT:** sometimes mosquitoes use the door as their entrance like here. You leave the door open and let mosquitoes enter if you have slept in the mosquito net and been in contact with the skin mosquito can transmit malaria if it is mosquito containing malaria.

**INTERVIEWER:** do you think people are aware that leaving open eaves facilitate malaria transmission?

**PARTICIPANT:** yes they are much aware, they are much aware that open eaves can facilitate malaria transmission

<Files\\Community participant chaziya-chapananga IDI> - § 5 references coded [10.39% Coverage]

Reference 1 - 0.91% Coverage

**PARTICIPANT:** what I know is that malaria is been prevented as we close house space and eaves

Reference 2 - 1.44% Coverage

**INTERVIEWER:** I want to know, how house improvement protects us from contracting malaria.

**PARTICIPANT:** house improvement protect us from malaria

Reference 3 - 1.11% Coverage

the problems are due to inability to understand but to those they easily understand there cannot be such problems

Reference 4 - 4.32% Coverage

**INTERVIEWER:** do you think there is relationship between house improvement and using the mosquito nets?

**PARTICIPANT:** things we do is to put gauze wire so that we can prevent malaria

**INTERVIEWER:** ok thank you. Do you think people feel to sleep in the mosquito nets after house improvement?

**PARTICIPANT:**  yes. People after house improvement they sleep in the mosquito nets because doctors keep on sharing so that they can reduce malaria burden

Reference 5 - 2.62% Coverage

**INTERVIEWER:** ok. Do you think people in your area are aware that open eaves increase the risk of malaria transmission?

**PARTICIPANT:** these people are true because leaving open eaves facilitate malaria transmission because there is entry of mosquitoes in large numbers.

<Files\\Community participant Garonga IDI> - § 7 references coded [11.58% Coverage]

Reference 1 - 1.00% Coverage

thank you. How do you think some people leave the open eaves at their houses?

**PARTICIPANT:** maybe the message has not yet understood by those people

Reference 2 - 3.20% Coverage

so as we are continuing our discussion, could you explain what you know about house improvement as one way of preventing malaria?

**PARTICIPANT:** yes I can explain. To prevent malaria first thing you close the eaves and finalizing you enter into the house checking where is the hole at the wall. When you see those small holes you paste the mud at it. After that you check at the window if the gauze wire has been put properly, this is when you see that you are done with the house.

Reference 3 - 0.55% Coverage

**PARTICIPANT:** it prevents malaria from having the chance of entering the house.

Reference 4 - 3.62% Coverage

**PARTICIPANT:** they welcomed it because when the gauze wire started coming the village headman called the community and address everyone on the reason of the gauze wire. So he said that we should first check for the holes at the wall and eaves if they are closed. So after checking for those we found bricks and start closing the eaves by then gauze wire were not yet been given to us. So we started maintaining the window frame if they were damaged and then we started receiving the gauze wire. And then they address us how it is supposed to put

Reference 5 - 0.89% Coverage

**PARTICIPANT:** most people complain about the darkness inside the house but they are not suffering from malaria but too much darkness

Reference 6 - 1.68% Coverage

**PARTICIPANT:** aaa maybe if they were wider windows and long gauze wire and put it at the window; so when the window is smaller and you decide to close this is when darkness comes in but if the window is wider enough there can be light inside the house.

Reference 7 - 0.64% Coverage

promoted/ improved?

**PARTICIPANT:** it is us to give the courage to those bringing the materials

<Files\\Community participant Goliati IDI> - § 7 references coded [10.99% Coverage]

Reference 1 - 0.82% Coverage

**PARTICIPANT:** I think those people really don’t know about severity of malaria

Reference 2 - 1.42% Coverage

how do you think the exercise of house improvement can be improved?

**PARTICIPANT:** it can be improved because we cannot be suffering malaria

Reference 3 - 1.44% Coverage

**:** do you think there is any relationship between house improvement exercise and using mosquito nets?

**PARTICIPANT:** there is no any other way

Reference 4 - 1.90% Coverage

**PARTICIPANT:** there is no any other way we can do without using mosquito net, because mosquito nets are found inside, so with the eaves cannot be linked, there are two different things.

Reference 5 - 2.63% Coverage

**PARTICIPANT:** yes it is possible they feel to sleep in the mosquito nets

**INTERVIEWER:** why is it important?

**PARTICIPANT:** because sometime there can be some openings like people are unable to seem the, since the mosquito is small can enter into such openings

Reference 6 - 1.99% Coverage

**INTERVIEWER:** okay. Do you think people in your village are aware that eaves contribute to transmission of malaria?

**PARTICIPANT:** yes, because when you leave eaves there is free entry of mosquito

Reference 7 - 0.79% Coverage

**PARTICIPANT:** the prevalence of malaria has been reduced other than in past

<Files\\Community participant Kabwatika IDI> - § 7 references coded [18.95% Coverage]

Reference 1 - 1.81% Coverage

could you explain what you know about house improvement which is happening in your village *(Repeating the question)*

**PARTICIPANT**: firstly closing the eaves and putting gauze wire at all the windows so that mosquitoes cannot have the place to bleed

Reference 2 - 1.33% Coverage

I understand properly this house improvement, but if you don’t take it positively malaria can increase rapidly, hence we should understand what committee is advising us what to do.

Reference 3 - 4.24% Coverage

**INTERVIEWER**: ooh okay. How do you think people leave open eaves? (Repeating the question)

**PARTICIPANT**: because they don’t understand what the committee is saying, if they understand they do accordingly

**INTERVIEWER**: ok we are continuing our discussion. Could you explain what you know about house improvement as one way of preventing malaria?

**PARTICIPANT**: yes

**INTERVIEWER**: explain

**PARTICIPANT**: closing the eaves putting gauze wire and if surrounding the house there is pit or well we fill in to avoid bleeding in of mosquitoes hence mosquitoes can’t find the way to bleed

Reference 4 - 4.77% Coverage

**PARTICIPANT**: it prevents malaria and also you should close eaves and put gauze wire and sleeping in the mosquito nets. It is not safe not to sleep in the mosquito nets even though you have closed the eaves and put gauze wire and if you go to the hospital you are likely to be found with malaria and make all possible to find mosquito net

**INTERVIEWER**: as the Village member of Kabwatika, how do you look at this house improvement project

**PARTICIPANT**: people are participating in house improvement activities so that the house looks smart and prevent malaria. And they are doing to prevent malaria and if malaria comes should be the other ways around

Reference 5 - 2.68% Coverage

what are the comments and expectations generally made by people in your village?

**PARTICIPANT**: the committee addresses us to build the house; close the eaves and we are given gauze wire to put at the window. And they make sure to give the wire unless the house has the closed eaves and they supervise house by house and they encouraged to report when the wire damages

Reference 6 - 2.10% Coverage

alright. How do you think house improvement standards can be promoted/ improved in your area?

**PARTICIPANT**: the committee should be calling for the community meetings during the meeting there are questions committee ask community and people answer. So those things motivates the committee

Reference 7 - 2.02% Coverage

: alright. Do you think people in your village are aware that open eaves facilitate the transmission of malaria?

**PARTICIPANT**: they are aware now because some people were deceiving this project and have been frequently suffering from malaria and now malaria is been reduced.

<Files\\Community participant Liwonde IDI> - § 4 references coded [10.03% Coverage]

Reference 1 - 2.02% Coverage

**INTERVIEWER:** how do you think people leave open eaves and other space in their houses?

**PARTICIPANT:** because of lack of knowledge, because they are hindering their own lives

Reference 2 - 4.10% Coverage

**INTERVIEWER:** explain what you know on house improvement intervention as one way of preventing malaria?

**PARTICIPANT:** we are supposed to close our windows with gauze wire and close the eaves and make sure when you are inside the house there should be no light coming outside, there should be darkness that is when we are assured that we are protected

Reference 3 - 2.26% Coverage

**INTERVIEWER:** how do you think people perceive these house improvement activities?

**PARTICIPANT:** they see that they cannot manage house improvement on their own; they only try to find bricks

Reference 4 - 1.65% Coverage

**PARTICIPANT:** this house improvement may be sometimes not easy; they don’t have materials to close eaves it is when mosquito enters the house

<Files\\Community participant Machokero IDI> - § 1 reference coded [3.74% Coverage]

Reference 1 - 3.74% Coverage

**PARTICIPANT:** these things go together. If you do HI but not sleeping in the mosquito nets then you didn’t protect yourself and if you do sleep in the mosquito nets but not doing house improvement activities you didn’t protect yourself to this because if you want to get out of the bed mosquito can bite you; this means that there is relationship

<Files\\Community participant MAGANGA IDI> - § 7 references coded [25.83% Coverage]

Reference 1 - 1.98% Coverage

alight. Could you explain what you know about house improvement which is happening in your area?

**PARTICIPANT:** gauze wire from Majete is been delivered to the chairman and we collect from them. And those houses don’t have wire or it is damaged collect from them. They advise us to put the wire at the window and close the eaves with bricks

Reference 2 - 1.40% Coverage

**PARTICIPANT:** it is like the owner of the house doesn’t have the standards but what is needed if it brick house should close the eaves so that air should not be entering. Suppose the air is flowing inside the house may fall down unexpectedly

Reference 3 - 2.60% Coverage

**PARTICIPANT:** frankly speaking this is durable method in our area because before we were building house anyhow but with the coming of this now our houses are smart and mosquitoes are coming in small numbers because our houses are smartly built because we use wire and now mosquitoes have nowhere to bleed. And there some people spraying chemicals in our rivers and wells and malaria have reduced, but with this high rainfall malaria has increased again

Reference 4 - 4.65% Coverage

**INTERVIEWER:** alright. What do you think are the perception of people towards house improvement?

**PARTICIPANT:** people are experiencing benefits

**INTERVIEWER:** could you explain

**PARTICIPANT:** as I have explained before that people were building houses anyhow as these houses with iron sheet you are seeing there we were leaving open eaves. So after coming of this intervention, now the houses are smart there is no need of improving the house because they are well improved. Once the wire is damaged; as some weeks ago we had to receive the wire which was easily getting rust but this phase they are giving us durable wire so once you put it no need of improving the house but if is damaged you just report to the chairman and get other wire. And the house needs to be improved close the eaves properly.

Reference 5 - 6.99% Coverage

**INTERVIEWER:** could you explain, what was the comments or expectations made by people

**PARTICIPANT:** people before were asking that why Majete people are giving us this wire and then Majete called the meeting addressing us that in our forest we have different kinds of animals and animals are been bitten by different insects. So thinking about the village because they are been surrounded by the forest they might be infected with the diseases frequently like been bitten by tsetsefly and other insects. so people might get different diseases and even mosquitoes bite some animals in the forest and later bite us so the disease burden might be high. So we are thinking of giving you gauze wire and be putting at your window. But before you should be closing eaves and plaster at your house; put your gauze wire at the window first so people welcomed the idea. But before, people were thinking that the Majete team has come to coerce the village later on to do something in their favour (majate) as it has been happening before. So then they advise to take care of our house and mosquito so that you should stay away with malaria. So with this idea community together with the village headman welcomed it up to date

Reference 6 - 1.60% Coverage

**:** we do but … especially to our houses

**INTERVIEWER:** what are the concerns? Let me hear those

**PARTICIPANT:** I explained already; when the wire at the window has been damaged we report, so they say it is out of stock wait, and we wait for months without gauze at the window

Reference 7 - 6.61% Coverage

**:** I want to know, is there relationship between using mosquito nets and house improvement intervention? [Repeating the question]

**PARTICIPANT:** no

**INTERVIEWER:** on the same question, do you think people feel important to sleep in the mosquito nets after house improvement?

**PARTICIPANT:** yes

**INTERVIEWER:** could you explain

**PARTICIPANT:** I mean that you can sleep in the mosquito net because we put the wire at the window only; because we sleep on the beds or sleeping mats it cannot be possible to have mosquito net. Suppose during rainy season the mosquitoes can hide and bleed inside the house and bite you if not sleeping in the mosquito nets, hence the mosquito nets prevent the bite of the mosquitoes and we should be able to use them

**INTERVIEWER:** alright. Do you think people in your village are aware that open eaves facilitate the transmission of malaria?

**PARTICIPANT:** no

**INTERVIEWER:** could you explain

**PARTICIPANT:** I can explain one side. We have people that have houses with open eaves in our area; we have plenty of such houses or even giving them advice they don’t do any action unless the chef has called for the meeting

<Files\\Community participant Mtemera IDI> - § 5 references coded [4.29% Coverage]

Reference 1 - 1.09% Coverage

they don’t know what they are doing? They don’t even know how malaria is spread. So when people advise them they feel that is not important that is why they remove.

Reference 2 - 0.51% Coverage

**PARTICIPANT:** they perceive it positively because we are protected from malaria

Reference 3 - 1.15% Coverage

**PARTICIPANT:** people say they are been helped since we are given gauze wire and giving us knowledge on what we should be doing in our area, hence been protected from malaria.

Reference 4 - 0.74% Coverage

**PARTICIPANT:** because in the community there are leaders so they can be doing that by making community meetings

Reference 5 - 0.79% Coverage

**PARTICIPANT:** because they are protecting their lives

**INTERVIEWER**: anything else?

**PARTICIPANT:** preventing malaria

<Files\\Community participant Weremu IDI> - § 2 references coded [1.85% Coverage]

Reference 1 - 0.91% Coverage

alright. How do you think people leave open eaves at their houses?

**PARTICIPANT:** misunderstanding of those people

Reference 2 - 0.93% Coverage

**INTERVIEWER:** how do you think people perceive house improvement intervention?

**PARTICIPANT:** they perceive it positively

<Files\\Community participant Zabuka IDI> - § 1 reference coded [2.85% Coverage]

Reference 1 - 2.85% Coverage

**INTERVIEWER:** could you explain what you know on house improvement activities which are happening in your area?

**PARTICIPANT:** there is closing eaves, sleeping in the mosquito nets and putting the wire at the window. Those people are doing what they have learnt hence there is decrease in malaria cases than before

<Files\\Community Participants-Yankho IDI> - § 1 reference coded [1.73% Coverage]

Reference 1 - 1.73% Coverage

# : I may not really know. But others leave gaps because they are feeling hot not knowing that in so doing they are allowing mosquito entry.

<Files\\FA-A Animators FGD> - § 10 references coded [27.34% Coverage]

Reference 1 - 2.86% Coverage

The house improving was good and people welcomed it. When the houses were collapsed, some took part to maintain them and now, they are alright

**P7:** In our village, people welcomed this and everything was done in collabolation, the problem however was that houses were collapsed due to rain and this was a set back to us, but this is going on well.

**P1:** House improvement in our community started well up to date. Everyone received this because from the time we started this project up to this time, the issue of malaria is no longer an issue, we hear from others

**P2:** We received this development quite well in our villages because at the start we were people that had no knowledge but when the project came and educated us on house improvement, everyone improved their houseon their own knowing the fact that we are protecting ourselves to have a longer life. All of the people in the village we agreed to do the work and have houses improvement of good quality with properly closed eaves and wire gauze placed on windows. So there is no problem at all in the village and everyone accepted this development whole heartedly and they are happy

Reference 2 - 3.82% Coverage

Thank you, let us proceed. Do you think house improvement is the necessary method of preventing malaria?

**P7:** yes, it is the necessary because when we close the house, mosquito cannot get in the house.

**P4:** This is necessary method because in the past, we were depending only on the net and yet with the nets, when getting out of the nets, mosquito get the chance to get in the net. When this method of closing the windows with the gauze wires, it has been a helpful method because when we close the open eaves, the mosquitos have nowhere to use when getting in the house

**P5:** This is good method because in the past, malaria was an issue but now, it’s been reduced through closing of house with gauze wires

**P3:** This is good method and there is no other better method than this because the gauze wires, closing of open eaves and sleeping under the nets, that’s one way that has brought the change here because previously, the malaria had been the issue here but now, malaria is reduced

**P1:** This is good thing although we are hearing that in the near feature, they will leave us. We are worrying because we cannot afford such development, this could have been continue, thank you very much.

**P6:** This method is good because it helps to reduce mosquito entry into the house

**P2:** This is good method because when we properly close the open eaves, the mosquito can get in the house if we open the door. There came a net distribution campaign that happened door by door, mosquito is not biting because we are sleeping under the net

Reference 3 - 1.82% Coverage

What are the activities involved when doing house improving task?

**P6:** We need bricks, water to be used when mixing with mud that is used when closing the windows

**P4:** There is need to find bricks, water for mixing mud, then the work starts to close the house.

**P5:** The materials that are needed are bricks, water and the builder if the owner cannot manage. The house should be inspected to see if there are gaps, so that everywhere should be closed

**P1:** The main issue is that the owner should first be willing to do the work, it is simple then. If the owner is not committed, he/she cannot get the bricks

**P3:**  We need bricks, water and the builders. You can do the work alone to prevent mosquito from getting into the house

Reference 4 - 4.44% Coverage

I want to hear from you, how do people think about this task? What is people’s expectation on this work?

**P7:** People have welcome this program very much because it has reduced malaria. People have changed their way of thinking because when they are building the house now, they are closing all open eaves to prevent mosquito from getting in the house.

**P3:** People have welcomed this program, when they are building the house, they close the open eaves that time. If they don’t get the gauze wire, they ask you when is the gauze wire coming, they have welcomed this program

**P8:** In the past, there was too much mosquito breeding in our homes but after this program was introduced, it has helped to reduce the mosquito in the houses. In the past, when you get in the house, it was as if you have kept the port of beer in that house but now, when you get in the house, you no longer have mosquito. If you get mosquito, it is their season but then you no longer have threats. People’s comments are that this program should continue, it should not just stop there

**P2:** This is very important program because people have learnt something. During the public meetings, they are able to express their views. Once they hear that there is a community meeting, they go in order to hear the problems that they are facing at the hospital like being shouted by the doctor, lack of medication and so they are happy because they are expressing their views freely

**P1:** My village was one of the villages that was trailing behind. I find it unbelievable nowadays that people come and demand for wire gauze at my place, so I see this as something that is very pleasing. What people never expected to change in my village has now changed. So in my village peope welcomed this development and are

Reference 5 - 1.55% Coverage

**P8:** People welcomed this method as one way of reducing malaria because when they are not suffering from malaria more often like before, so everyone received it

**P3:** People welcomed this because malaria is now a history, they are able to save money after doing business. In the past, they were not able to keep money because they were going to the traditional healers when the child gets sick, but now when this program came, they welcomed it

**P5:** people are able to see the fruits of this program because in the past, they were often getting sick from malaria than now, people are now able to comply with the HI program

Reference 6 - 4.05% Coverage

When this program came in our community, people were doubting, they were asking, what we are going to eat once we go there or how much I am going to be paid when I go there. They started noticing that this program is benefiting them and it is not about getting money, they changed and they started using it properly

**P5:** At the beginning, people were thinking that we want to get rich through them. They started receiving it through the community meetings that we were conducting, they are following it now

**P2:** That time people could not understand this, they were asking some questions like, ‘if you put gauze wire in the window, how are we going to breathe, you want us to die in the house?’ we then started teaching them that this gauze wire has small holes but it prevents mosquito from getting inside the house. They started understanding it little by little and now, everything is okay

**P8:** People were talking a lot in the communities, they were referring us to Satanism. They were saying that the mosquitos that they are taking from our communities, they have their methods of sucking blood from us so that we should die. After we sensitized them, they started taking this method to be very important and not what they were thinking at the beginning.

**P4:** people were ignorant because they were thinking that the blood sample that are taken from them, they were thinking that it is business.

**P2:** Just to add, when this method came, and we were given the nets, others refused to sleep under the nets saying it is itching and they are not able to do sex in the family because of the net. Everything now is over

Reference 7 - 3.11% Coverage

I think there is no any other method apart from this one in our community

**P8:** I think there is no any other method in our communities apart from this one. The only way we can add is that we should level all stagnant waters or making the water ways

**Facilitator:** How do you think the method of house improvement should be improved?

**P5:** For this to be improved, the skills that we have gained throughout and the unity between the health animators and the village headmen as well as the committee members and the community members, this work can be improved

**P3:** We should continue to work together with the committees, chiefs as already said by my friend here, we need to progress together because this is our development.

**P2:** Just to add, this is very important. When this project is phasing out, we shall continue because when you were coming here, you wanted to protect us. This is our right to start improving our houses. If we are not united, this can longer continue. We will continue with this because the money that we were using to buy medication, is the now spared

**P8:** I am also grateful to the non-governmental organizations that are taking part in the development of this area and this has helped us a lot in the community.

Reference 8 - 1.03% Coverage

There is a relationship, if we do house improvement, and there is a hole somehow in the house and a mosquito has access to entry, the mosquito net forms a barrier for protection to this mosquito.

**P7:** The relationship is that after we have done the house improvement, when we are getting in the house, the mosquito can get in as well. When we are sleeping under the net, the mosquito cannot bite us anymore

Reference 9 - 3.14% Coverage

On that same note, do you think when people improve their houses, they think it is necessary to sleep under the nets?

**P1:** it is necessary as my colleague has said that most of the houses have collapsed due to heavy rains and some people are sleeping in the bad condition and they sleep under the nets without problem. Everyone therefore should have a net at his/her household.

**P3:** It is necessary because closing of open eaves is not enough, we should also be sleeping under the nets because the mosquito can get in through the door and if you don’t sleep under the net, you can still catch malaria. If you sleep under the net, the mosquito will not achieve what it wanted to do

**P4:** I think this is possible because when you sleep under the net, you prevent a lot

**P5:** It is necessary because the first method was to improve house by closing the open eaves and putting of gauze wire in the windows but we use the door when getting in and out of the house, the mosquito gets in the house and then it finds you sleeping under the net. I believe these methods are similar and we should use both

**P2:** just to add, sleeping under the net is the necessary method because you prevent many insects from getting onto the bed and w cannot suffer from any illness

Reference 10 - 1.53% Coverage

Do you think people in your community have realized that leaving the open eaves unclosed can facilitate the spreading of malaria?

**P7:** they have realized because once they don’t close the eaves, mosquito get inside the house but if you close open eaves and sleep under the net, some people get out of the net because of smell, they are beaten by the mosquito

**P6:** Just to add, if we don’t close open eaves, we breathe a lighter air and it gets out through these open eaves, the mosquito smell that gets in through the wall and gets the person

**P3:** Just to add, both methods are good because you protect your life

<Files\\FA-A Community participants FGD 2> - § 16 references coded [23.38% Coverage]

Reference 1 - 1.05% Coverage

This work was difficult at first because we really did not understand what closing the eaves meant. Thereafter, we were taught that closing of eaves helps in prevention of malaria. Once we were taught, we started closing the eaves and cracks in the walls of the houses with gauze wire. We then saw that we were having fewer people getting malaria than it had been before. Thank you.

Reference 2 - 2.30% Coverage

This work had been difficult from the time it was stating because we did not know what it meant. Besides, it was difficult to be told to close the eaves with gauze wire because we thought we wouldn’t have enough ventilation our houses especially in house season. With time of being of taught, we got to understand that we can prevent malaria through this work. Thank you.

**Facilitator** P5?

**P5:** At first when this project started, the people in the committee visited us. It was difficult to understand what they were explaining to us. Following subsequent visits and encouragement by them, we understood that it was good to close the eaves and cracks in the wall so that they would come to put gauze wire to prevent malaria. We understood and now we have closed the eaves, and we are not frequently getting malaria as before. Thank you.

Reference 3 - 1.89% Coverage

House Improvement is appropriate in prevention of malaria because malaria mostly comes due to mosquitoes. So because of this, it is appropriate for us to prevent malaria.

**Facilitator:** Thank you. That’s the idea of P4. P6?

**P6:** In addition to that, I think it is appropriate because it is the only way that us helping us prevent malaria. Closing of eaves with gauze wire I think is one thing that is helping us reduce malaria.

**P2:** In addition to that, it is one way that is helping us. When we close eaves it means mosquitoes will not be finding way into the house. So in summary, if mosquitoes do not enter the houses, it means mosquito will not bite us and we will suffer from malaria.

Reference 4 - 1.56% Coverage

Alight. Are there any other ideas? Alright, let us now proceed to another issue. I would like to hear from you as villagers. Would you explain to me what you know about House Improvement as one way of preventing malaria?

**P5:** Closing the eaves with gauze wire is one way of preventing malaria because mosquitoes will not be able to get into the house. So we can prevent malaria.

**P6:** In addition to that, closing the eaves prevent the mosquitoes from getting into the house and helping us not to get bitten by mosquitoes. So I think it is one way I think is helpful.

Reference 5 - 1.14% Coverage

P1.

**P1:** When we do House Improvement and sleep under mosquito nets in our houses.

**Facilitator:** Alright. Is there anyone a different idea?

**P6:** It prevents mosquitoes from entering the house.

**Facilitator:** Okay. That is P6’s idea. Any other ideas? Let us open up. Let us hear other people’s ideas. How does House Improvement help us prevent malaria?

**P3:** It helps to prevent mosquitoes from getting into the house

Reference 6 - 3.82% Coverage

Alright. Proceeding, I would like to hear from you as the villagers and perhaps you can also talk about your friends in the village how they felt. In your own understanding, what is your and other people’s attitude towards House Improvement work?

**P2:** We have positive attitude towards this work because it is making houses look good. Thank you.

**Facilitator:** That is P2’s idea.

**P4:** People did not think it was good at first. When they realized they were being protected from malaria, they started doing house improvement work.

**Facilitator:** Alright. On the same issue, would you explain to me how people look at this work of House of Improvement. What were people’s expectation or ideas about the House Improvement work?

**P2** People were saying that before the House Improvement work came in people were more frequently getting malaria than they are getting doing now. They are appreciating this work. Thank you.

**Facilitator** Alright.

**P3** People are appreciating this work. They want it to continue so that they should prevent malaria. Thank you.

**Facilitator** Alright. P7?

**P7** Since the House Improvement program started in 2015 up to now, there is been a change in frequency of episodes of malaria. People can have symptoms of malaria but they are not being found with malaria at the hospital. We are differentiating the current situation and the way it was before this work. Thank you.

Reference 7 - 1.55% Coverage

People welcomed this work and they are happy that there is reduction in malaria prevalence in the area.

**Facilitator** Alright, we have heard from… (*interrupted by P6)*

**P6** People are appreciating the work and they are being helped in that they are not frequently suffering from malaria any longer.

**P3** People are appreciating the work. They are saying it is a good work as it is protecting them from malaria.

**P5** People are appreciating the work because there is reduction in prevalence of malaria

**P1** They are appreciating the work… (*????)* now there is been a change.

Reference 8 - 1.99% Coverage

People were talking about heat in their houses after closing the eaves.

**Facilitator** Alright. That’s P5’s problem.

**P2** There is no ventilation. The air from outside cannot enter the house.

**Facilitator** Alright. That’s from P2’s village. Any other problems or disadvantages that were being said? P7?

**P7** People were saying that it was dark in the houses.

**Facilitator** Alright.

**P6** People were saying that the gauze wire was getting damaged during windy rains.

**Facilitator** Aright. Are there any other problems? Aright, we can continue. Different problems or grievances have been raised. What do you think happen to sort out these problems as raised by P6 concerning the gauze wire?

**P1** Tack the gauze wire inside the house.

Reference 9 - 0.44% Coverage

I think the reliable way is to close windows and eaves as well sleeping under mosquito net. This is the most reliable way than maybe other ways we can think of.

Reference 10 - 0.18% Coverage

I think there is no other way that can be more effective than this

Reference 11 - 0.64% Coverage

If someone is constructing a house, they should tell the builder not to leave spaces. Additionally, they should make sure that they have completed constructing the house without leaving spaces as we were doing previously. Thank you.

Reference 12 - 0.32% Coverage

The village committee members should continue teaching us on House Improvement. Then this project will be sustained.

Reference 13 - 0.84% Coverage

There is a big relationship because if you do house improvement but you do not sleep under mosquito net, there is no protection against malaria. You will still get malaria. On the other hand, if you close the eaves with gauze wire and sleep under mosquito net, you will be fully protected against malaria.

Reference 14 - 1.29% Coverage

It means you are completely protected from mosquito bites because you are sleeping under insecticide treated nets.

**Facilitator** Alright. Let us hear other opinions.

**P2** There is a relationship. The mosquitoes enter the house in the evening through the open doors before you even go to bed. So if you are sleeping in an improved house as well as under the mosquito net, you cannot get mosquito bites from the mosquitoes that enter through the open door in the evening.

Reference 15 - 2.28% Coverage

It is important because if we open the door some mosquitoes will find their way into the house. So if we do not sleep under mosquito nets, we are not fully protected. However, if we sleep under mosquito nets, we are protected. It is very important.

**Facilitator** Alright. That is P3’s idea.

**P6** I think it is important because there could still be some spaces in the closed eaves through which the mosquitoes could manage to enter the house. So the mosquitoes could still be biting us if we are not sleeping under mosquito nets.

**Facilitator** Alright. That’s P6’s opinion. P7?

**P7** It is important to sleep under mosquito nets because when we are opening the door in the evening, mosquitoes can enter the house. So if we do not sleep under mosquito nets, we will be bitten by mosquitoes. However, if we do, we will not be bitten.

Reference 16 - 2.08% Coverage

Yes, people in the village have realized. We really have realized because many mosquitoes find space in the open eaves.

**Facilitator** What space?

**P5** Space to enter the house.

**Facilitator** Alright. That is P5’s idea. P1, do you have any ideas?

**P1** It is what they are saying because if you do not close the eaves, many mosquitoes enter the house. But if you close the eaves and all spaces, and sleep under mosquito nets, mosquitoes do not enter the house.

**Facilitator** Alright. Perhaps someone wants to explain more on that.

**P3** It is true. Most of us have realized that many mosquitoes enter through the open eaves. These mosquitoes enter and bite us. But now we have realized that many mosquitoes enter through these open eaves and there is need to close.

<Files\\FA-A HI Committee FGD> - § 10 references coded [22.90% Coverage]

Reference 1 - 1.71% Coverage

HI committee, would you tell me what you know about closing of open eaves as one way of preventing malaria?

**P8:** one method that we know for malaria prevention is that if our houses are properly closed, then we have prevented malaria and if we sleep under the nets, our window have gauze wires, these are some of the things I know

**P7:** the thing is that after we have done everything, like inserting gauze wires and closing all open eaves, that’s the way of preventing malaria

Reference 2 - 1.19% Coverage

how the house improving work protects us from malaria?

**P4:** it prevent mosquito from getting into the house

**P6:** it prevent the mosquito from getting into the house

**P8:** it helps us so that only few mosquito should get in to the house because if we have done everything including sleeping in the nets, then we have prevented malaria

Reference 3 - 3.90% Coverage

Thank you, let’s proceed. How do people in your communities view this work of house improving and also what are the comments that people have on the house improving work?

**P5:** This work is going on well because people have welcomed it. There is reduction of disease in the communities.

**P2:** just to comment on that, I have heard some people commending this work saying that we think this work is preventing us from getting malaria. ‘’It’s good method to follow, if the donors continue with this, malaria will be reduced’;, that’s what people were saying

**P3:** most of people in Mtemera are thankful and when everyone builds the house, the first one to approach before start using the house is the committee member so that they can go to do the work first, thank you very much

**P7:** people welcomed this because when this project had begun, people did not know the danger of malaria. After this project, when people started being sensitized on malaria, they stop hating each other as they bewitch each other not knowing that it’s malaria. People welcomed this from that time up to now.

Reference 4 - 1.41% Coverage

Just to proceed from there, do you think people welcomed this as one way of reducing malaria in your communities?

**P1:** yes, people welcomed this because we taught them this

**P4:** In our community, people were happy when we told them on how to prevent malaria and how malaria is spread

**P8:** people fully welcomed this project and they are thankful that this project should continue, thank you

Reference 5 - 5.47% Coverage

okay, let us continue. I would like to know, what are the challenges that people were talking to be facing when following this HI procedure?

**P2:** In the communities, people were saying that if we close the open eaves and the gauze wires, there shall be little air in the house to breath and that could lead them to suffocate. We were telling them that the gauze wires cannot be compared with the window grass because air can easily circulate through the windows. So they welcomed it

**P6:** the problem that people were talking is not to insert the gauze wires because cats cann not get into the house using that window

**P3:** As Mtemera community, people who keep cats at their household were saying that if we close the windows, cats will be in difficulties to get in the house but after we teach them that cats could use the same entry that they are using, they understood it and up to know, they are saying that cats are now using the same entry

**P7:** in our community, some people have chicken and they don’t have kholas to keep them and because of that, there is a room in the house that chicken use and therefore, these chicken use the same windows when going to that room and if you close there, it won’t work. We were asking them if they to go to Mwanza hospital and being admitted, would the chicken survive, they will sell all chicken to pay for medical bills, it’s good then to close these open eaves so that chicken may use the same entry as long as they prevent malaria, they changed and they welcomed the idea

Reference 6 - 0.77% Coverage

For someone who had these challenges and did not consult the committee member, it was indeed a challenge. If we knew someone who had such challenges, we were going to encourage them and he/she could understand it.

Reference 7 - 1.26% Coverage

let us proceed. How do you think this work can be improved?

**P7:** This can be improved because community members welcomed this and it cannot be difficult even in the absence of committee members

**P8:** We as committee members should not just sit back and watch. We should have time once in a while to call for the meeting to remind people, thank you.

Reference 8 - 1.74% Coverage

would like to know, is there any relationship between house improving and the use of mosquito nets?

**P2:** there is relationship because if we close the open eaves, to some extent, mosquito can easily get into the house. The relationship is that we should not be discouraged to sleep under the mosquito nets every night.

**P1:** we should sleep under the nets, there is such relationship.

**P8:** I would like to add on that because if we don’t sleep under the nets, we can catch malaria.

Reference 9 - 1.93% Coverage

Do you think when people have done house improving, do they see it necessary to sleep under mosquito nets?

**P4:** They think it’s necessary because the moment we open the door, mosquito gets into the house

**P6:** Yes, when we have improved the house, we should as be sleeping under the nets because we prevent malaria. At around 6 to 7 o’clock, mosquito start flying and it uses the door entry.

**P5:** We chat up to around 7 and the door is still open, mosquito easily get into the house so if we sleep under the mosquito nets, we are safe.

Reference 10 - 3.53% Coverage

Do you think people in your communities think that leaving the open eaves unclosed can facilitate the spread of malaria?

**P8:** In Kabwatika, people know that if they leave these open eaves, malaria can spread because the time they were not trained, they don’t think leaving the open eaves could help spreading malaria but after we trained them, they know that they can catch malaria, thank you.

**P1:** After people got trained, they knew that this can help to spread the malaria because mosquito can easily get in the house

**Facilitator:** I would like to know from you the committee members, how do you see the activities that are there when improving the house?

**P7:** The house improving job is not difficult because if someone starts building the house now, he already close the open eaves, so the work is now simple

**P8:** This is not a tiresome work because it’s a group work between the owner and us the committee members

**P1:** We just facilitate but it is the owner who does the work

<Files\\FA-B Animators FGD> - § 15 references coded [31.37% Coverage]

Reference 1 - 0.79% Coverage

it’s a very useful method because prior to the project coming here, there used to be a lot of people getting sick with malaria. But today only a small number of people get sick with malaria, showing that closing open eaves and putting gauze wire on house windows is reducing malaria.

Reference 2 - 0.89% Coverage

okay, whose responsibility do you think house improvement is?

P3: it’s the responsibility of the house owner to improve their house – if they are supplied with gauze wire, they have to close open eaves and seal any holes, so that they may have safety when they put gauze wire on the windows and sleep under a mosquito net.

Reference 3 - 2.57% Coverage

okay, could you describe what you know about house improvement as one way of reducing malaria?

P5: house improvement is about closing open eaves and closing house windows with gauze wire. It also involves checking whether the door is properly inserted for cutting down entry of mosquitoes into the house – so that only 10% of the mosquitoes enter the house should they at all manage to enter. This method is very useful because it’s what’s causing a reduction in malaria in this area – closing open eaves, closing windows, proper insertion of the door, plus sleeping under a mosquito net.

P3: what I know [about house improvement] is that it’s about closing small openings, closing windows with gauze wire, and encouraging sleeping under a mosquito net. That’s the only way we can reduce malaria.

P2: sleeping under a mosquito net every night, closing our house windows, and sealing small openings that can let mosquitoes in.

Reference 4 - 2.71% Coverage

in your understanding, how does house improvement help to reduce malaria?

P3: it helps in that if house windows are closed with gauze wire, small openings sealed, and people are encouraged to sleep under a mosquito net, that can reduce malaria.

P5: when we were undergoing training on house improvement to reduce malaria in the village, we were taught that the government has been distributing mosquito nets with the idea of reducing malaria, but malaria seems to increase. That’s when there was this idea of house improvement to see if malaria can be reduced. Now, in the villages where this project has been implemented, malaria seems to have reduced. That’s what gives us understanding that house improvement is one way of reducing malaria.

P2: house improvement helps to reduce malaria because if your house is not properly improved, sealing all the openings that are there, then sickness with malaria will never free your household. So house improvement is very important.

Reference 5 - 3.53% Coverage

in our village, they received it because of the benefits of closing windows with gauze wire. Because house flies, cockroaches have no entry into their houses. Because of this many people have been very positive about closing windows with gauze wire and closing open eaves.

P3: yes they received it because before distribution of gauze wire, people used to have malaria a lot. But after realizing the benefit of closing windows with gauze wire, sealing small openings and sleeping under a mosquito net, they are experiencing a reduction in malaria in their villages.

P5: people have been very positive about house improvement as one way of reducing malaria, because when we got reports [from households] about gauze wire wearing out and forwarded the reports to the office, if the office delayed to dispatch the gauze wire, people would come to us and complain, saying, “You are now putting us at the risk of malaria because you taught us about the danger of malaria, we have closed open eaves, but gauze wire is not being supplied. This will cause us to go back to our previous life.” This was because those that were doing malaria tests were finding that most people did not have malaria. This was evidence to people that house improvement was one way of reducing malaria.

Reference 6 - 1.88% Coverage

yes people had complaints about house improvement because when constructing their houses they did not close open eaves. So, when they closed open eaves and windows with gauze wire after we had encouraged them, they started complaining, “The house is too hot, better when the windows had not been closed because there was ventilation.” Also, a year after they had closed the windows, the gauze wire started rusting, and they were complaining that “Worn out gauze wire makes our house look dirty.” When reported this to the office, the office supplied new gauze wire and people replaced the one that had worn out, and they were commending the new gauze wire because it was durable.

Reference 7 - 1.48% Coverage

regarding complaints about the house being too hot, we would explain to them that “What we have done is for reducing entry of mosquitoes into your house, because if we don’t give you gauze wire and leave the windows open, then we have not protected you. We are giving you gauze wire so you can close your windows, not to make your house look good, but to prevent mosquitoes from coming into your house, so malaria can be reduced or stopped completely. If the house feels too hot, you just have to bear it out.” And they would accept it.

Reference 8 - 3.19% Coverage

complaints were there related to gauze wire. Some, because of religious beliefs, would refuse to close windows with gauze wire. But when they understood they started closing windows with gauze wire without forcing them, after seeing benefits that other households experienced regarding sickness with malaria – they would see differences where some households never had sickness with malaria and others always had sickness with malaria. That made them accept that closing windows with gauze wire was useful.

P5: the other complaint people had about house improvement related to nails, which seemed to affect the progress of how many houses were closing windows with gauze wire. Because when we told them that they were to look for nails themselves, they would say, “Better if you were providing nails too, then we would close the windows right away.” But we would tell them that “You could cut spokes and use them for nails.” Those that had spokes could use them, but those that completely did not have nails or spokes would take time to close their windows with gauze wire. Committee members would at times help such people with nails if they had any.

Reference 9 - 0.48% Coverage

if the office could also provide nails beside gauze wire, that would be good. If the office can’t provide, then we are going to be advising people to use wires for nails.

Reference 10 - 1.84% Coverage

to respond to your question, they discourage people in that there are some who use gauze wire when they receive it, and there are others who just keep it because of lack of nails, but both of them received the gauze wire on the same day. So, for those that receive the wire after others already received it, they feel discouraged, seeing that others received but are not using it. The problem is shortage of nails. Some women are not married and maybe have no money for nails, so they receive the wire and just keep it. So, sometimes committee members cut spokes for nails and help people with those, because there are elderly people that have problems raising money.

Reference 11 - 1.61% Coverage

house improvement could be promoted through us animators holding meetings, where we can teach people how they can improve their houses, so that they may be motivated for the work.

P4: I am thinking that if we the villagers decide together to stop malaria, then this work can be promoted. Without relying on the office, if we the beneficiaries personally show much interest, and because of experiences of benefits of house improvement by households that already implemented it, then this work can be promoted.

P7: house improvement can be promoted if people do what we teach them.

Reference 12 - 1.51% Coverage

okay, let us move on. In your view, is there any connection between house improvement and using a mosquito net?

P3: yes there is a connection because if someone has a mosquito net, they have to close their house windows [with gauze wire] so that malaria-transmitting mosquitoes do not enter the house.

P6: there’s a connection between house improvement and using a mosquito net because if someone closes their house windows, should mosquitoes find entry into the house through the door, the person won’t be bitten because they are covered by net.

Reference 13 - 2.97% Coverage

okay, in your view, do you think people still find sleeping under a mosquito net useful after implementing house improvement?

P2: after implementing house improvement, people still find using a mosquito useful because of what we taught them. We taught them that house improvement and using a mosquito go together. If you implement house improvement but don’t use a mosquito net, should mosquitoes find entry into the house through the door, then you are going to be bitten by this malaria-transmitting mosquito.

P7: if you implement house improvement, you still need to sleep under a mosquito net because mosquitoes have different entries into the house. The gauze wire might have a small hole and mosquitoes might come in through that. So, if you are not using a mosquito, the mosquitoes are going to bite you, and if the mosquitoes that bite are those responsible for malaria transmission, then you will have malaria. So we encourage them to sleep under a mosquito net all the time and all year round even when they have closed open eaves and windows with gauze wire.

Reference 14 - 3.85% Coverage

let us move on. In your view, do people in your villages now understand that not closing open eaves contributes to malaria spread?

P4: we could say that today most people understand that it’s a huge difference between a house with unclosed open eaves and a house with closed open eaves in terms of entry of mosquitoes into the house. A house with unclosed open eaves lets in more mosquitoes than does one with properly closed open eaves.

P1: yes people now understand that leaving open eaves unclosed contributes to the spread of malaria because lots of mosquitoes enter the house through the open eaves. But if you close open eaves, there are less mosquitoes entering the house, they enter through the door when you open it and move out. But if you sleep under a mosquito net, mosquitoes won’t bite you.

I: okay, how do open eaves contribute to malaria spread?

P5: open eaves contribute to malaria spread because malaria-transmitting mosquitoes, anopheles mosquitoes, claws into the house through the wall and detects your body smell through the open eaves. That’s how not closing open eaves contributes to malaria spread.

P3: open eaves contribute to malaria spread because malaria-transmitting mosquitoes may come into the house through that, and if you are not using a mosquito net on that night, the mosquitoes might descend from the walls and bite you, making you sick with malaria.

Reference 15 - 2.06% Coverage

okay, what do you think about house improvement as animators?

P7: house improvement is good because people that we encouraged to improve their houses are now commending. Before house improvement, sickness with malaria never freed their homes. But today they are testifying that malaria has reduced because of what taught them to do.

P4: um, this method is very good because houses also look good when open eaves are properly closed. Also if windows are closed with gauze wire, because people in the villages are poor and can’t find money for buying glasses for their house windows. So, if you close them with gauze wire, the houses look very good.

P2: house improvement is very good, it’s the only way that has reduced malaria in our area.

<Files\\FA-B community participants FGD> - § 16 references coded [50.91% Coverage]

Reference 1 - 3.33% Coverage

Firstly, we gladly received this project. We did not know how important House Improvement is. However, when this Majete Malaria project came, teaching us to improve houses and tacking gauze wire, it helped in the reduction of malaria. So, I feel this has helped us a lot, especially in our village.

**Facilitator** Alright. Thank you. Is there anyone with a different opinion?

**C2** In line with what my friends have said, I think it is one of ways that has made many families to be healthy and protected. So, if this work can continue, there will be reduced malaria prevalence.

**Facilitator** Alright. Is there anyone else with other opinions?

**K3** I am glad for this new method of capturing mosquitoes both outside and inside the house. Previously, they were only capturing outside the house yet some mosquitoes were already in the house. Now that they are capturing both outside and inside the house, all the mosquitoes are being trapped. I think this is a very good method.

**Facilitator** Thank you very much. Is there anyone else with other additions?

**B1** I will just emphasize on this kind of project. For instance, us as B1, we did not know how we could defeat mosquitoes. Then, this project of tacking gauze wire in the windows and closing all spaces to prevent entry of mosquitoes into the house came. It has helped us a lot because we have known how we can defeat mosquitoes as well as malaria. We have known that we can defeat malaria by improving our houses by tacking gauze wire and closing all the spaces to prevent mosquitoes from entering the house. This method is protecting us and malaria prevalence is reducing. This method is helping us a lot. It is supposed to continue so that we prevent transmission of malaria in future.

Reference 2 - 0.68% Coverage

This is a very good and very important method because putting the gauze wire in the window only without closing the eaves cannot work. This is why we start with closing the eaves. This is because we know that the gauze wire only helps in the windows. I think it is a good method and we need to encourage people for their houses not to have any spaces.

Reference 3 - 0.83% Coverage

My opinion is that this is a very good method. It is helping us because previously we used to have a lot of mosquitoes in the house. However, we are not having a lot of mosquitoes in the house nowadays with the closing of eaves and tacking of gauze wire in the windows. Mosquitoes can only enter the houses when we are careless by leaving the doors open in the evening. Otherwise, this method is very helpful. Thank you very much.

Reference 4 - 0.48% Coverage

This project has helped a lot because it has reduced spread of malaria.

**Facilitator** Are there any additional opinions?

**K1** We are also thankful for teaching people to be applying chemicals in stagnant water. We think that is also helping us a lot.

Reference 5 - 1.88% Coverage

Alright. Thank you very much. The other thing that I would like you to explain to is what you know about House improvement as one method of reducing malaria.

**C1** House Improvement helps to reduce number of mosquitoes. This is because mosquitoes lay eggs in the unclosed eaves. This makes the population of mosquitoes in the house to increase. However, when you close the eaves, less mosquitoes enter the house. Thank you.

**Facilitator** Alright. Anyone with other views? K1, do you have any additions?

**K1** Okay, only on what C1 has said. When you close the eaves, you prevent mosquitoes from entering the house. Additionally, when we burry stagnant water like in rainy season like this or apply chemicals in them helps prevent mosquitoes from getting to the houses. This is because mosquitoes are also found in the stagnant water. Together with closing of eaves, they do help us.

**Facilitator** C2?

**C2** When we tack the gauze wire, it protects us from snakes. It also reduces malaria.

Reference 6 - 4.66% Coverage

Thank you. Finalising on this question, I would like to know your opinions. In your own thinking, how does House Improvement help reduce malaria? Let us start with B1.

**B1**  Would you repeat the question?

**Facilitator** I was saying, this House Improvement method, how does it help reduce malaria?

**B1** It is true how you have asked your question. It helps reduce malaria because if you have followed what you have been taught that you are supposed to close the eves and secondly, tack gauze wire to prevent mosquitoes from entering into the house. Thirdly, make sure that the door does not have spaces. This is one of the ways that can prevent spread of malaria in the household if you have done all the requirements. This will protect you from mosquitoes which can bite you and infect you with malaria.

**Facilitator** Thank you very much. K3?

**K3** When we tack the gauze wire, we should make sure the house does not have spaces. This is to make sure we see the important of tacking gauze wire. When the house has got so many spaces, we could be saying the gauze wire is not helping yet we have not followed the procedure.

**Facilitator** Would you briefly explain its exact work?

**K3** Okay. When infected mosquitoes enter the house, people suffer from malaria yet the gauze wire has been tacked. Then, in the villages we will be saying “Even if they have given us the gauze wire, it is not working because we are still getting malaria. Why then are they saying this helps prevent malaria yet we are still getting malaria”. It is all because we do not really realise that we are getting malaria because of the small spaces through which malaria infected mosquitoes enter into the house.

**Facilitator** Alright. Thank you. Z1, you have additions on this?

**Z1** No, I don’t have.

**Facilitator** Yes, C1.

**C1** Adding on to that as well, we should be doing our best to sleep under mosquito nets every day. This is because we may be have tacked the gauze wire but mosquitoes can be entering the house through the door. So, we really to be sleeping under mosquito nets. Thank you.

**Facilitator** Thank you. K4?

**K4** This method is helping a lot because few mosquitoes enter the houses. Additionally, as C1 has said, we need to be sleeping under mosquito nets. This is because the time we are opening the door, mosquitoes do enter the house and can then bite people. However, if we sleep under mosquito nets, we will reduce malaria prevalence. Thank you.

Reference 7 - 3.38% Coverage

Thank you. Let us proceed. In your own thinking, how do the people in the village perceive this House Improvement work? ***(Question repeated)***.

**K2** This was difficult for people to understand this work before we realised its importance. Now that there is reduction in malaria prevalence and we are not being troubled by mosquitoes, people have gladly taken it up and it has become easy. In addition to that, we have been safe these past years, many families have been healthy, children are able to attend school because there is reduction in number of mosquitoes which infect people with malaria.

**Facilitator** Thank you.

**C2** This method is good because since the coming of Majete Malaria Project there is been reduction of malaria prevalence here in Chikwawa. Thank you.

**B1** We should congratulate this Majete Malaria Project for teaching us this House Improvement work to prevent mosquitoes from entering the houses. We, as B1, are very thankful for this. This is because there was no one who knew exactly this method of improving houses by closing spaces and tacking gauze wire and how it protects. When this project came, we learnt its advantage that when we tack the gauze wire and close the eaves, we prevent mosquitoes from getting into the house and prevent malaria. So we are asking that this project should continue. You should help us reach to those who did not manage to get the gauze wire so that everyone should be protected from malaria. Thank you very much.

**Facilitator** K1, do you have any additions on this?

**K1** We are just glad for coming and giving us the gauze wire. There was a lot of mosquitoes previously before the coming of this work. There seems to be a reduction in number of mosquitoes currently because of this House Improvement project.

Reference 8 - 2.75% Coverage

Okay. Thank you very much. In your own opinion, how did the people in your village accept this House Improvement work? ***(Question repeated)***.

**C2** In our village, people accepted this project because there is been reduction in the prevalence of malaria compared to previous times in the village since the coming of this project. Additionally, sleeping under mosquito nets is a good way of reducing malaria.

**Facilitator** Alright. On the same issue, would you explain what people in the village were saying about House Improvement work or what were their expectations of the House Improvement work? Firstly, you explain to us how people accepted this work and then you can add the people’s expectations or perceptions.

**B1** People in our village accepted this project very well, and everyone is appreciating the expertise of tacking gauze wire and closing eaves to prevent mosquitoes from getting into the house. So, people in our village accepted this project very well and everyone is happy because of it. As B1 village, we very much want to have additional gauze wire to give to those who did not receive at first. This is because only few people received the gauze wire. So, as B1, we would like everyone to receive gauze wire so that we reduce the number of mosquitoes as well as the prevalence of malaria. Thank you very much.

**Facilitator** Z1, perhaps you would explain how people in your village accepted this House Improvement work?

Reference 9 - 3.41% Coverage

Thank you. In your own thinking, did people in your village accept this House Improvement work as one of the ways of reducing malaria?

**K4** People accepted this method because of the research that showed that it is the only way which is supposed to be followed. People gladly accepted this project till now when people want to replace the damaged gauze wire. Thank you very much.

**Facilitator** C1, do you have any additions on this?

**C1** It is true, as explained, that many houses have been damaged by rains in this 2019 rainy season. So, I am hope that many houses will be built after the rainy season. Therefore, it is my request to the Malaria Project that if there still donors they should try their best so that we have the gauze wire available to continue reducing malaria prevalence. Otherwise, there will continue be high prevalence of malaria because currently some people are staying in camps where the mosquitoes are biting both infected and non-infected individuals. That way malaria prevalence will continue increasing. So, I am requesting Majete Malaria Project that when people are done constructing houses, you should give us the gauze wire to reduce malaria prevalence. Thank you very much.

**Facilitator** Thank you. K3, would you explain to us if people in your area accepted the House Improvement work as one way of reducing malaria?

**K3** People accepted this method in our village because nowadays fewer people are suffering from malaria than previously. The mosquitoes are mainly biting us outdoors when we are chatting. We go up to nine o’clock when we are chatting in the evening. This the time that malaria infected mosquitoes start biting. So, people in our village accepted this House Improvement work because there is been a reduction in malaria prevalence.

Reference 10 - 7.40% Coverage

Than you. Let us proceed. Are there any other concerns that people in your village raise pertaining to this House Improvement work? Challenge are always there, right? So, I would like to know any concerns that people in your village raise pertaining to the House Improvement work? Let us start with K2.

**K2** The concern is that the first gauze wire that we received was not lasting longer. It was getting damaged by rust so easily. The one that just came is different. It seems to be nylon type. When send report that we have run out of gauze wire, it takes longer. People complain because during this time they are not safe. So, our request is that when you receive a report that gauze wire is needed, try your best to consider our requests as soon as possible to address people’s concerns in the villages.

**Facilitator** K1, are there any other concerns that people in the village raise pertaining to House Improvement work?

**K1** The thing is that when the gauze wire is damaged, we are not at peace in our households because the windows are not closed with the gauze wire. So, when we tell the committee members that my house does not have gauze wire, they say the gauze wire is out of stock. It takes long time for have the gauze wire available. This is when we start suffering from malaria frequently because there is safety.

**Facilitator** Alright. B1?

**B1** I will only emphasize on what K2 has said. It is true that the main problem is the delay to have the gauze wire available. People start questioning as to why it is taking long to have the gauze wire in stock. When we tell them that we have sent a report and we will have the gauze wire in stock soon, people do not believe. Therefore, we are asking your office that you should be trying to deliver the gauze wire when it is needed so that when we speak, people should believe us. That is the we meet frequently; the delay to have the gauze wire available. Many people do need the gauze wire because they have realised that it is protecting them. So, we are asking your office to make it quick so that everyone should be safe. Thank you very much.

**Facilitator** Is there any one with additions on this issue of concerns which we have been receiving in the villages we are coming from? Alright, let us perhaps continue on the same issue. What should happen to address these concerns? Let us start with K2.

**K2** It is not difficult to address these concerns. There should be a quick organization between the animator and the office. This will make it easy on the people. This is because… This is because when we have the gauze wire tacked, we become safe and get used. So when the gauze wire is damaged, we easily get malaria from the mosquito bites when get while waiting for the gauze wire. Therefore, the office has to be quick in making the gauze wire available in the villages to address people’s concerns.

**Facilitator** Alright, thank you. K1, do you have any additions on what should happen to address these problems? B1?

**B1** I will only emphasize on what K2 has said. It is true that there is need for proper organisation between the office and the animators chose in the villages so that the reports are sent in time. If the gauze is out of stock today, for example, they should write a report on the same day and send to the office. They should not be far away from the office because if they are away from each other, there will be a delay to get the materials to the villages. So, my point there is that there should be a proper organisation between your office and the animators in the village so that you should be close them. In that way, you can quickly give them the materials or update them. Otherwise, we will be complaining and troubling the animators. We are, therefore, asking your office to be close to the animator so that the gauze wire should be available to us. Thank you very much.

Reference 11 - 1.80% Coverage

Thank you. Alright. In your own opinion, how are these concerns encouraging or discouraging people in your village in as far as House Improvement work is concerned? ***(Question repeated).***

**K2** If it has been reported and there is delay to have the gauze wire available, someone will be discouraged to even close the eaves when they still do not have gauze wire. This is because they will still need gauze wire when they close the eaves or spaces. This means when you are telling someone to close the eaves or spaces, you need to have gauze wire available…

**Facilitator** Alright, thank you. C2?

**C2** Someone will be discouraged. For example, you start foundation when you have the materials and you start preparing the field for planting when you have the materials. If we have the gauze wire available, people in the village will not be discouraged because they will be seeing that the gauze wire is available and then they can start the work.

Reference 12 - 3.04% Coverage

Alright. Is there anyone with additions of this? Alright, I would also like to know your opinions. In your own thinking, how can the way improving houses, that has been followed, be promoted?

**C1** This method can be promoted if animators can be encouraging the people by, for example, conducting meetings to tell them the advantage of improving houses and the disadvantages of not improving houses.

**Facilitator** Thank you. K3? ***(Question repeated)***.

**K3** This method of improving houses can be promoted by telling the surrounding villages, which have not improved their houses, the importance of House Improvement in prevention and reduction of malaria in the village. When you explain to these villages about house Improvement, they admire it and develop interest to have the project in their villages. In that way, it gets promoted because more villages are taking it up.

**B1** The most important thing is for the animators who chosen in the villages should call for meetings to tell the people the importance of the project and how to promote it. If animator cannot be in the forefront, the other villagers cannot do what is supposed to be done. The most important thing is the need for the leader to call for a meeting to tell people the importance of the project. That way, it will be done easily. Thank you very much.

**K4** On the issue of animators, they have to be properly taught about the method, and be encouraged that they can also encourage other people. If they are not being properly taught, they cannot be able to teach other people the proper methods. Thank you very much.

Reference 13 - 5.75% Coverage

Thank you. In your own thinking, is there any connection between House Improvement and using mosquito nets?

**K2** Yes. There is a connection between use of mosquito nets and House Improvement. We do not tack gauze wire on the door. Mosquitoes can get into the house when we are outside chatting in the evening. If there are uncovered buckets of water in the house, the mosquitoes find a place to stay till next morning. So we need to be very careful. It can happen that we have tacked the gauze wire or we are sleeping under mosquito net yet …is in the house as well. Then we have done nothing. We need to take care of the pots because when the mosquitoes find some water, it will have found a place to stay. We have to be careful on that.

**Facilitator** Thank you.

**K4** House Improvement and use of mosquito nets are helping a lot. We have to be sleeping under mosquito nets because the mosquitoes that enter into the house through the door, will be able to bite the people inside. So if we are also sleeping under the mosquito nets, we will be helped a lot. Thank you.

**Facilitator** Are there any additions on this issue about any relationship between House Improvement and use of mosquito nets? Alright, let us continue. I also would like to know on the same issue. In your own thinking, do the people see the need to sleep under mosquito nets when they improve their houses?

**K2** We need to keep on encouraging each other on this issue. We are saying that even if we improve our houses, we do not tack gauze wire on the door. This is the reason everyone has to be sleeping under the mosquito net every time. In addition to that, it is very hot here in Chikwawa and the time comes when we cannot sleep in the house due of extreme temperatures. These mosquito nets can be used when sleeping outside as well. So, even when the house has been improved, everyone is supposed to sleep under mosquito nets every time.

**B2** Use of mosquito nets and House Improvement good. This is because mosquito nets protect every individual. On the other hand, gauze wire, though it is good, children still get malaria when they are not sleeping under the mosquito nets. However, if we sleep under mosquito nets every year, our children will be protected from malaria.

**B1** I only want to add. It true that when we improve our houses we should also be sleeping under the mosquito nets because mosquito nets are very important. As K2 has explained that we should not depend on one thing only because we will not be helped on the other part. This is because when we are outside chatting in the evenings we leave the door open. The way K2 explained, we do not put gauze wire on the door. Therefore, we should not be saying that we will not use a mosquito net because we have closed the eaves and tacked the gauze wire, no. we are supposed to have a mosquito net because when we open the door, mosquitoes enter the house. So, mosquito net is very important to everyone to prevent malaria to prevent malaria. Thank you so much.

Reference 14 - 2.30% Coverage

Thank you. Are there any final opinions on this issue? Alright, let us continue. I would also like to like to know. In your own thinking, have the people in your village realised that leaving open eaves promotes the spread of malaria?

**C1** People have realised this because of the coming of Majete Malaria Project. Previously people did not know that when we leave eaves unclosed, mosquitoes will get into the house. People did not know that. The coming of Majete Malaria Project has made them realise that by leaving the eaves open, they were making the mosquitoes get into the house. I hope the coming of this project has made us, the people in the village, realise this. Thank you very much.

**B1** I only want to add. It is true that there no one who knew what happens when we leave the eaves open. However, the coming of Majete Malaria Project and choosing of animator in the villages who called for the people and told them about the project, then everyone knew that leaving the eaves open is one way that can spread malaria due to mosquito bites. Everyone realised that mosquitoes are the ones that spread malaria and everyone now is closing their eaves to prevent malaria. Thank you very much.

Reference 15 - 4.01% Coverage

Alright. Are there any additions before we proceed? Okay. I would also like to know another thing that, what is your perception about the House Improvement work? ***(Question repeated).***

**K4** This method of House Improvement is very good because it is one way that is helping us to prevent mosquitoes from getting into the house. Thank you very much.

**Facilitator** Perhaps in addition to that, I also would like to know how the House Improvement work done.

**K4** We are taking this work to be easy because people realised its importance. When someone has been given the gauze wire, they will not take for granted. Everyone is taking part because they are seeing its importance.

**Facilitator** Alright. Are there any other opinions on how we perceive the House Improvement work? C2?

**C2** No, I don’t have.

**Facilitator** B1? ***(Silence).*** K2, do you have additions on this issue? ***(Question repeated).***

**K2** House Improvement is a very important thing. As K4 has already said, it is now part of us. When a house is not improved, we say it hasn’t been finished and we cannot sleep in it until we close the eaves because we are not used. So, everyone realised that when we say a house is done it means the eaves have been closed. We can only sleep in it when the gauze wire has been tacked as well. If someone has an un improved house… It means he or she has not managed already. Honestly, sometimes we sleep with children and you cannot always check that the child is inside the net. So, tacking of gauze wire has to go together with making sure the children are properly sleeping under the mosquito nets. Additionally, we also have children sleeping in other rooms. We should try as parents to make sure the children are safely sleeping under the mosquito net. Either a father or a mother can tack the mosquito net for the children. This is because when the child suffers from malaria, it will be the parent who will struggle to get him or her to the hospital. At the time, the child will miss in his or her education because they will not attend school due malaria. So, parents let us be guards of our children

Reference 16 - 5.21% Coverage

Thank you. Alright, let us continue to another section. In your own thinking, are all the houses in your village properly improved? The villages where we are coming people are doing House Improvement, right? ***(Question repeated).***

**B1** I will explain like this; most of the houses are properly improved. However, as we have explained at first, the main problem is that many houses have fell down due to this season’s heavy rains while others have been damaged in areas we closed. There is need to close in all such areas again. So, these are the problems, as I have explained. Thank you.

**Facilitator** K1, what can you add on this issue? Are all the houses in your village properly improved?

**K1** Some people do not properly improve their houses. When the animators come to inspect and when they find that your house is not properly improved, they tell where to close. So, when you follow the instructions, you do improve the house properly.

**Z1** There are some people in our village who cannot properly do the work. So some people help them with the work until its done.

**Facilitator** Thank you. Is there anyone who would want to add on this issue?

**K4** It is not all the houses which are properly improved for other reason. The reason is that sometimes some houses are improved by children because they do not have an older person who can do the work for them. So, a child usually does not do the proper work. Most importantly, I would like to encourage our friends the animators and their committee that they should be helping such people so that proper work is done. Thank you very much.

**Facilitator** Thank you. So I would like to know on what makes us realise that a house has been properly improved? When we are in our villages, what makes us say that this house has been properly improved? C1?

**C**1 When you are outside, it shows that the wall is in contact with the roof all around. It’s what shows that the house has been properly improved. You have properly tacked the gauze wire in the windows, you have plastered and painting. The people say that he properly improved the house.

**Facilitator** Thank you.

**B1** I just want to add a little bit as C1 has said. It is true that …the house. For example, when you get inside the house and close the door, and you up the wall, you are able to see where there are spaces. So where there are spaces, you get the required materials to close. Additionally, the other thing that makes us realise is that when you do not sleep under the mosquito nets at night, mosquitoes bite you. The mosquitoes enter the house through the open spaces. It is then that you realise that you have not properly improved your house and I need to look for the open spaces. That is how you realise it.

<Files\\FA-B HI Commitee members FGD> - § 14 references coded [31.54% Coverage]

Reference 1 - 4.88% Coverage

Thank you, let’s proceed. Do you think the house improving is a good method to reduce malaria?

**P1:** it has helped because when we close the open eaves, it was difficult at the beginning, but we were trained on closing the open eaves as well as gauze wire. We noticed that it’s good thing because we could sleep in the house without mosquito. We therefore that there is more advantage to the closing open eaves.

**P5:** just add on the closing of open eaves, it is helping and when we close with gauze wire, the mosquito gets in the house. There is another method which Majete project helped us, they gave us mosquito so that we should sleep under mosquito. This has helped us in the way that if 100 mosquito were getting in the house, now only a few will be getting in the house because we closed open eaves and we put gauze wire and we sleep under the net, I should stop, thank you very much.

**P4:** I should add on that one. The method is good because it has shown us the difference from the time we did not have such method, we were suffering from malaria because it was infecting more people, there was no protection. These methods of gauze wire and closing open eaves have contributed to the increase of immunity and malaria has been weaken. The method is good and if this continues, malaria will be very reduced in future comparing with this time

**P8:** this method of gauze wire is good one and people are commending the method because children were getting sick from malaria more often. They were fainting and they were diagnosed with malaria. With the nets that were distributed, the closing of open eaves, children are not suffering from malaria often, I thank the malaria project for this programme. If this project is phasing out, they should do such project in another area because we were luck to receive the gauze wire but in other communities, there is no such work. They are admiring us when we close the open eaves and gauze wire, anything that want to get in the house, it’s trapped there because of the gauze wire, thank you very much

Reference 2 - 1.61% Coverage

okay, let us proceed. Can you explain anything you know on the closing of open eaves as a one way of malaria prevention?

**P3:** closing of open eaves is the way of preventing the malaria. We were also trained that if we have closed the open eaves, we should get in the house to see where light is coming from. If found, you should take the soil to close it and after that, you can be assured that there is security. That’s what we were trained

**P2:** I learnt that after closing the open eaves, you should get in the house to see if there is a hole so that you can cross it. You should sleep under the net. When getting in the house, you should always shut the doors, thank you

Reference 3 - 1.83% Coverage

okay, let us proceed. How closing of open eaves protects from getting malaria?

**P8:** closing of open eaves protects in the way that when closing the open eaves, there is little mosquito getting in the house. When we do not close the open eaves, mosquito get in the house anyhow. We also make sure we close any gap

**P5:** the same issue especially at the door. If the door has got gaps, we should get the sawdust to close so that mosquito should not get in the house through it. If there is a gap between the doorframe and the wall, we should close the gap, thank you very much

**P4:** this method reduced the number of mosquito that were getting in the house. If there were 2 mosquitos that was getting in the house, only one or none gets in the house, it’s the good method

Reference 4 - 1.34% Coverage

what do you think people in your respective communities people think of this house improving work?

**P8:** this work people think it’s a good because everyone put the gauze wire and closed the open eaves. People are asking for this wire.

**P7:** people now welcome this gauze wire because the time there was no this wire, malaria was the issue but now, the chief and his subject are feeling okay because of this gauze wire that has come. When everyone build the house, he/she is coming to take the gauze wire, they are therefore feeling okay because malaria is reduced

Reference 5 - 3.29% Coverage

okay, let us proceed. Do you think people in your community received the house improving method as one of the malaria prevention measures?

**P1:** people accepted this method although it was difficult at the begging as we have already said but now, we can confirm that they received it

**P3:** the way question has come, people received this as one way reducing the malaria

**P8:** yes, people received this because they are saying now malaria has been reduced

**P4:** people in our community received this but at the beginning, we could not recognize this as one way of preventing malaria. We therefore received it just to try because others had fears that the gauze wire is blocking oxygen from getting in the house, such things others were saying that the gauze wire had rust, that first one, it was therefore a trial at the begging but now, people have discovered that this is one method of preventing malaria

**P5:** people receive the method but as others have said, the first gauze wire could not last for a month before rusting, but when we complained to Majete project that the gauze wire is not lasting longer, they sent us another gauze wire of aluminum, everyone appreciated the gauze wire and we are asking Majete malaria project that if they want to bring gauze wire, they should bring the aluminum gauze wire than the first grade gauze wire they brought at the beginning, thank you

Reference 6 - 5.00% Coverage

what are the challenges people were speaking concerning house improving method?

**P3:** at the beginning, people were not closing the open eaves, as long as they are sleeping in the house, it was okay and when you tell them to close the open eaves, he was worrying because it was an addition work on the house that they are sleeping in it. When we told them, they understood it.

**P4:** the challenges that people were talking were the oxygen circulation in the houses before the closing of open eaves, there was enough oxygen but after the closing of open eaves, there is not and they were saying that we should not bring them such gauze wire. Another thing is that the first gauze wire could catch the rust easily, people were saying that your wire is making my house dirty, you should not bring again your gauze wire, others were insulting us, others saying that we are doing a job which we are not getting anything and when we complained, they brought us this aluminum gauze wire which has changed everything

**P2:** people were asking what were they supposed to with the gauze wire , I w as telling them that the gauze wire is for window and you should close the open eaves so that no mosquito can get in the house. Others were saying that they did not have bricks for closing of open eaves especially those that do not have husbands. I was telling them that they can go where they were molding the bricks to have pieces of bricks and use them for closing of the open eaves, mosquito will not get in the house

**P6:** at the begging you gave us the councilors who were conducting the sensitization. When the village chief had called for the meeting, people were coming for and most of the days, there had been the training about closing of open eaves and the gauze wire. People are now appreciating and they are asking for another gauze wire. They are saving money because in the past, they were spending money for transport to go to the hospital where if they did not get malaria treatment, they had to spend lot of money to go to another facility but now, things are okay with the coming of this project.

Reference 7 - 2.29% Coverage

okay, you have talked of challenges, how do these challenges are encouraging or discouraging people in your communities on the house improving work?

**P2:** it is encouraging people, they say when I put gauze wire, I am not getting sick often. I am also sleeping under mosquito net, thank you

**P4:** just to add on the challenges that discouraging people, if people were continuing talking of these, this development could have stopped, we could suffer from malaria as before. We were meeting some people who were saying that some animal survive when eating another animals, if you put gauze wire in the windows and close open eaves, how will mosquito survive if it will not suck blood from you, are caring the wild animal? We could find the way so that such discouragements should not continue. We were encouraging them that yes that’s true but there are some animal that spread disease like. Animals like mosquito and housefly, we should stop that from happening

Reference 8 - 2.34% Coverage

this program I should say is improving because people when people have built the house, instantly they are closing the open eaves, they do not wait to be visited, so the programe is improving

**Facilitator:** okay I will read the question again. The procedure that has been followed, how can this be improved?

**P9:** this work can be improved by having the meetings and also encouraging the community members that they should not stop doing this work, they should continue closing of open eaves and gauze wire because it is helping us. Majete Malaria should not stop helping us

**P4:** just to add. We the committee members, people have been talking about us. To improve this, we should have a motivation so that when we are going to the communities, we should be motivated despite that the work if voluntary. When people shout at us, we should be able to say though people are shouting, this our benefits. In short, our training as a committee members should be motivate not only Fanta

Reference 9 - 0.74% Coverage

okay, you have talked of motivation, how can this be like?

**P4:** we the volunteers, when we meet with people from the office, we take them as our bosses. The motivation is that the trainings should be conducted often times but there should be money so that we can receive at the time we have gone to the training

Reference 10 - 1.53% Coverage

do think there is a relationship between house improving and the use of mosquito net?

**P6:** there is relationship because mosquito nets help when we are sleeping but mosquito can get in the house because children can leave the door open, so mosquito nets help to prevent mosquito from biting us while the closing of open eaves and the gauze wire help to prevent mosquito from biting us

**P1:** mosquito nets help us as my friend has said that may be children have gone for play, when they come back, they leave the door open and when you sleep under mosquito net, you are safe. When we close the open eaves and the gauze wire, they all helps us

Reference 11 - 1.16% Coverage

Do you think when people improve house, do they see the importance of sleeping under mosquito nets?

**P5:** we think people take it very important when they close open eaves and sleep under mosquito net because they are protected from mosquito.

**P4:** all methods are necessary because they are protecting malaria. If someone has closed the open eaves but he/she does not sleep under net, mosquito can get you. If you are sleeping under the net, you are protected more than the other methods.

Reference 12 - 0.55% Coverage

Do you think people in your communities know that the closing of open eaves help in preventing malaria spread?

**P7:** at the beginning, they were not, but afterwards, they started knowing that leaving the open eaves can cause malaria

Reference 13 - 0.99% Coverage

how can open eaves facilitates the spread of malaria?

**P7:** mosquito gets in the house if there is open eaves

**P6:** mosquito gets in the house because through open eaves. We were trained how mosquito gets in the house

**P3:** mosquito gets in the house if the open eaves are not closed. We were trained that if do not close the open eaves, the mosquito smells us and it forced itself to penetrate through the open eaves

Reference 14 - 3.99% Coverage

okay, how do you see the house improving work?

**P8:** others who could not close the open eaves, we the committee members could go and help them to close the open eaves so that they can benefit as well

**P4:** it was a very big task. When we bring the gauze wire at the household, we were forced to bring the nails as well. The elderly could spend 3 to 4 days without putting the gauze wire, we could go and help them

**Facilitator:** as a committee members, was the job simple or tiresome?

**P5:** the job was simple though tiresome but for now, we get used to it up to now

**Facilitator:** what made the job to be simple?

**P5:** there was unity as committee members. If someone is failing to close the open eaves, we the committee members could agree to go and do the job

**P2:** the work was tiresome because we could measure the wire and cut, we could close the open eaves. If we give them the wire, others could say where am I going to take the wire, I will not afford

**P4:** the work was tiresome because others could leave the wire carelessly and the wire could have damaged, so we could not allow that wire to be used. We were taking the wire and give them another wire. Sometimes some committee members could not come and the work that was supposed to be done by 4 people, it was done by 2 people and hence, the work was tiresome

**P9:** just to add, the work was tiresome because in the area where people keep animals like cat, this new gauze wire that we are saying it does not rust, the cats destroy it, then the owner would tell us to bring another gauze wire. So it was tiresome to think that yesterday you gave them the gauze wire, should I give them another, it was so tiresome

<Files\\FA-C Animators FGD> - § 11 references coded [24.92% Coverage]

Reference 1 - 2.19% Coverage

closing open eaves and fixing windows is one method that is helping people here in our community to reduce malaria incidence such that the number of people attending the hospital because of malaria has considerably reduced. Also, the money that’s normally spent on managing sickness has been saved.

F: anything else to add on this?

P3: just commenting on what P1 stated, it’s a very useful method because people were being educated on how the malaria transmitting mosquito works, so closing open eaves and putting gauze wire on the windows was very helpful because the number of mosquitoes entering the house reduced. And because we’re encouraging them to sleep under a mosquito net they were still being protected from the few mosquitoes that were still able to enter the house. So, it’s a very useful approach because it reduces the number of malaria transmitting mosquitoes entering the houses.

F: anything else to add on this?

P6: this project about closing open eaves is very helpful because people are personally testifying that it’s reducing the number of mosquitoes entering the house compared to previous experiences of being in a house with open eaves and no gauze wire – there used to be lots of mosquitoes in the house. Entry of the few mosquitoes that still manage to enter the house happens when people move out to relieve themselves at night. But if they sleep under a mosquito net, the few mosquitoes don’t bite them. So, it’s helping to reduce malaria.

Reference 2 - 0.56% Coverage

just to add on how useful the project has been, people are also testifying that closing open eaves has further protected them from winds because previously the roof would easily be blown off because of winds entering through the open eaves. But today they are killing two birds with a stone; they are ensuring their safety from infection as well as of their houses from winds.

Reference 3 - 3.18% Coverage

could you tell me what you know about closing open eaves or HI as one way of preventing malaria?

P2: what I know is that, because we’ve got several lives: life of a child, life of an expectant mother, life of an elderly person, and life of a healthy person like me. We safeguard these lives by closing open eaves, so that these people are not severely affected. The elderly and a child, for instance, are in a vulnerable state. The expectant mother is equally very vulnerable. That’s why we very much emphasize that they should sleep under a mosquito net from evening till morning, and that they should not leave open eaves unclosed.

P3: the HI method is being a very useful safety method. Why? We learnt that malaria transmitting mosquitoes climb the house walls to enter the house. So, if we properly close the house windows with gauze wire, then malaria transmitting mosquitoes won’t be able to enter the house. If they’ll manage to enter the house, then it will only be a few of them.

F: the other thing I would like to hear from you about the same issue of closing open eaves as one way of preventing malaria is that what are the other activities that people do when closing open eaves?

P6: what I know concerning the project about closing open eaves is that once we’ve given information to the community, then we decide on how to close open eaves. We can close open eaves with bricks. If we are going to close open eaves with bricks, then we decide how we are going find the bricks, and people may decide to mold them separately or together as a group. Once we have closed the open eaves, we close the windows with gauze wire. We nail the wire on the window using nails or small wires cut into nails. If the house is made of mud, we nail the wire using small sharpened sticks and then stick it using mud. When we have closed the open eaves, sometimes there are still small openings, which can’t be closed with bricks; we close them with mud. The other thing is that our doors have spaces that can let mosquitoes in. So we look for small pieces of timber and fit it in the spaces, so that mosquitoes have no entry into the house.

Reference 4 - 2.58% Coverage

anything else to add? [Silence] Alright. So how does closing open eaves stop us from catching malaria?

P1: closing open eaves stop us from catching malaria because malaria is what causes us to lose household resources because our money is spent on managing malaria, buying drugs. But if we close open eaves and sleep under a mosquito net, it means we have protected the life of a child, or my life if I am pregnant.

P3: closing open eaves is very useful. First, it reduces entry of mosquitoes into the house. That means those staying in the house are protected from malaria infection because there are no mosquitoes entering the house. Safety from malaria infection in turn means they can fulfil their development plans properly.

P2: I just wanted to comment on that, if someone is sick with malaria, they can’t function properly. Why am I saying this? If your house has open eaves, you will never cease having [malaria] infection, and frequent illness with malaria also affects other families who are not sick. In addition to that, there’s higher incidence of malaria within the village – because when neighbors visit you they may get bitten by the same mosquitoes that bit someone with malaria. Eventually, malaria spreads to the entire village. So we encourage people to close open eaves so that there’s complete reduction of malaria to 0%.

P6: I agree with what my friends have mentioned that malaria spreads through mosquitoes. By improving the houses, we are preventing mosquitoes from entering the houses. Entry of mosquitoes into the house happens at night, when we are asleep. So if open eaves are closed it means mosquitoes will have no chance of biting us. I therefore feel this method is useful for reducing malaria.

Reference 5 - 1.67% Coverage

as animators, how do you think people in the village feel about the house improvement project? And if you could describe the comments and expectations that people in the village have concerning this.

P2: talking of people’s comments, today I had 3 people who we’re asking me, “Did the project about closing open eaves finish?” I said, “No, we still have gauze wire and more is coming.” “My house window is broken.” I said, “I know. We are already in the process of counting houses with broken gauze wire.” And people are happy with the way we are inserting the gauze wire. Everyone - chiefs and households – are grateful and requesting that this project should continue.

P5: many people are happy with this project about closing open eaves because for example if someone’s house window has broken gauze wire, they persistently come to you, saying, “I will die sooner, mosquitoes are troubling me so much,” because when they insert gauze wire they are able to sleep. They feel sleeping under a mosquito net with open windows does not help because they are still exposed to mosquitoes when they get out of the net to wee.

Reference 6 - 2.24% Coverage

anyone else with anything else to add on this topic about comments and expectations that people have?

P3: people are very positive about this issue. In the beginning, they felt it was not useful. But today they are appreciating it. Why? They are seeing a difference in the amount money they would spend previously and what they are spending now. Why? Because they are experiencing less cases of malaria infection in the house – because there are no mosquitoes entering their house. So today they are much interested in this methods considering how much money they previously would spend on malaria. They are even saying that “If the project is delaying to give me the gauze wire I am going to purchase it on my own. Because the annual cost of taking a child to the hospital because of malaria is more than the cost of gauze wire.” I have been hearing these comments from people about gauze wire.

P1: adding on what my friends have been saying, people in the village, in their comments, are very grateful for what the Majete Malaria Project has been doing, providing gauze wire. They are very grateful. They are commenting that they are less mosquitoes entering their houses, and that they have been able to protect their lives. They are also saying that they have been able to reduce some of the problems that they face in the village.

P4: when we distribute the gauze wire, there are some from other villages who also want it – we tell them that “It’s only for this area.” But they also need it so much.

Reference 7 - 2.26% Coverage

alright. You have told me about how people feel about this method. But I also want to hear that in your opinion do you think people received this method as one way of preventing malaria?

P5: people received this method without problems. Of course, there were some who had problems accepting it. But most of them improved their houses without problems, closing open eaves, inserting gauze wire on the windows and closing small openings.

P6: in agreement with what P5 said, people reacted very positively to this project about house improvement. Why? Because they were in charge of improving their houses. Whenever we’re out in the villages, people from neighboring villages were asking, “When are you going to start in our village?” That means people have been positive about the project.

P3: people have been very positive about the project. They have been closing open eaves without problems. They have been buying nails without problems. Meaning they welcomed it with both hands. That’s why they have been participating without problems – because they welcomed it very positively.

P2: just commenting, people received this thing without problems. Why? Because people, seeing that gauze wire is delaying to come, have been closing their house windows using used mosquito nets. And when the gauze wire arrived they felt their houses would look the same as houses in town. That means malaria in our community will reduce. And people are grateful for the coming of the Majete Malaria Project; it’s helping them a lot.

Reference 8 - 4.46% Coverage

alright. In your opinion, how could the procedures for house improvement be improved?

P6: for the house improvement procedures to be improved, um, as an animator, holding community meetings is enough. Also if the MMP (Majete malaria project) team could have village visits once every 3 or 6 months and speak about the project so that people understand. That will also make people respect [animators] to say, “This person is sent by an office,” because there are some that despise you. The other approach we have used previously is forming a team of say 4 animators and holding a village meeting together, especially if an animator had problems addressing people in a particular village. Further, since a project works on timelines, and given that in a village there are some who can’t close open eaves on their own such as the physically disabled, or the elderly, or orphaned kids, we and the committee members together would volunteer to say, “Tomorrow let’s go and work on such and such houses.” We would also help them with nails because these groups of people were really troubling us, which would make the committee members and us go and look for spokes. Then we would cut the spokes into nails and nail the gauze wire for them. So, to ensure progress of the work, we would volunteer to support because in the villages there were others that wouldn’t buy these materials on their own.

P5: When we went for the training, we’re promised that they would be coming to monitor how the work was being done. But there has been no other monitoring visit since the initial visit and people have been asking: “Why are they not coming to visit us anymore? Did the project finish? Or they are just dealing with you now, not interested in us anymore?” So, as a matter of request, committee members should be visited for the continuation of the work.

P2: to ensure uptake of the project, we use houses that are along the roads as demonstration houses, so that everyone passing by can see and ask: “Whose house is this?! How are they doing this?! Where are they getting these things from?!” So, we tell them the organization that provides those things. We explain to them how it’s done, and that the project is currently implemented in one village as a trial and will later roll out to other villages. People from other villages are also interested and asking, “Are you selling the gauze wire?” “No, it’s not for sale.” So when the project rolls out to other villages you will hear, because in the villages where the project is being conducted people are no longer asking us questions. So the project will be accepted widely because we have houses along the roads serving as demonstration houses.

P3: regarding the challenges we’re experiencing such as people not being able to find nails, especially the elderly or child headed households that have no means of support, supporting them with nails would make things easier. Also, continued training of us the animators would ensure smoothing running of the work

Reference 9 - 1.72% Coverage

Thank you. In your opinion, is there any connection between the house improvement method and using a mosquito net?

P4: this method of using a mosquito net is not very reliable. Why? Because after using it for a few days it tears. But the gauze wire was of two types: one that rusts and another that does not rust. Up to this day, it’s [the one that does not rust] still fine. So, that [the mosquito net] does not seem reliable because can tear any time.

P1: I feel the connection between the house improvement method and the method of using a mosquito is that you need to use a mosquito even when the house has been improved in case mosquitoes manage to come into the house.

P3: I feel the two methods are closely related. Why? Because mosquitoes that still manage to come into the house won’t be able bite you because you are covered by a net.

P6: conquering with what my friends, there’s a relationship. Because we can’t reduce malaria by house improvement alone. Why? Because out of 100 mosquitoes 10 might find entry into the house. Therefore, if you are sleeping under a mosquito, the 10 mosquitoes won’t bite you. Then we have prevented malaria.

Reference 10 - 2.26% Coverage

on the same issue I would like to know – do people still find sleeping under a mosquito useful when they have improved their house?

P5: yes, it’s useful to sleep under a mosquito net [even after house improvement] because if you don’t then you are preventing malaria, you will regularly be suffering from malaria.

P1: you need to sleep under a mosquito whether a pregnant woman or any other person. But a pregnant woman has a very higher risk if she doesn’t sleep under a mosquito net. She needs to sleep under a mosquito net to protect the unborn child from getting born with abnormalities or malaria infection.

P6: I feel people still find sleeping under a mosquito net useful despite their houses having been improved. How do I know this? I usually have meetings with the people, and during the meetings I teach them about the benefit of closing open eaves and the benefit of sleeping under a mosquito even when the open eaves have been closed. The other thing is that when we are making household visits we ask them to show us where they sleep – we find that most of them have a mosquito net set up for use. So yes people feel it’s useful to use a mosquito net.

P3: through the lessons that we give them, people feel it’s very important to sleep under a mosquito because we teach them that [closing open eaves] only keeps away 90% of the mosquitoes, but 10% of them may still find entry into the house and cause malaria. So, we advise them that they sleep under a mosquito net for a complete malaria prevention.

Reference 11 - 1.80% Coverage

alright. To wind up this section, I wanted to know – do people now realize that leaving open eaves unclosed facilitates malaria transmission?

P2: people in my village have completely understood that. Because we would give them examples and they have personally seen differences – households with open eaves unclosed were experiencing regular malaria infection. Then they would ask, “Why is it like this?” “You are leaving in an improved house and their house is not improved, and the mosquitoes that we’re teaching you about...” We have pictures of mosquitoes that we collected from the Majete Malaria Project. We show the pictures to the people: “Do you see this house? Could this house be the same as that house?” “No.” “The mosquitoes you are seeing enter such kinds of houses.” So people have learnt and lots of houses have had open eaves shut, and people are appreciating what this is doing them for them.

F: anyone with additional comments?

P5: just to add on what P2 said, it appeared most people understood the value of closing open eaves. But those that did not close open eaves had experiences of regular illnesses – when they realised that closing open eaves was beneficial they closed theirs too.

<Files\\FA-C community participants FGD 2> - § 9 references coded [26.64% Coverage]

Reference 1 - 2.78% Coverage

Alright. Let us continue. In your own thinking, why do some people leave eaves unclosed in their houses? **(*Question repeated for clarity)***

**P5** This gives problems. The problem is malaria. Mosquitoes enter the house through the open eaves. That is why you need to quickly tell them to close the eaves because we are having a lot of diseases due to mosquitoes. Malaria is very dangerous the whole world.

**Facilitator** Alright. P5, what I wanted to understand is the reason why people leave those spaces open. Alright, perhaps let us start with P7 then P6.

**P7** Those spaces are difficult to close perhaps because it needs a way to climb up there to close. They would, for example need a ladder to close the eaves. That is why others just leave them open. On the other hand, this makes mosquitoes enter the house.

**Facilitator** Alright.

**P6** The problem here in our village is that previously people… big windows. We then encouraged each other the windows are good but they need to be closed. They were leaving open eaves for ventilation in their houses. But all this ended.

**Facilitator** Alright.

**P4** Some refuse to close the eaves because of termites. They have the idea that when they close the eaves and the wall gets in contact with the roof, termites will easily get to the roof.

Reference 2 - 2.81% Coverage

Alright, thank you. Let us continue our discussion. Would you explain what you know about House Improvement as one way of preventing malaria? ***(Question repeated for clarity)***. Let us start with P1.

**P1** It is a way of preventing malaria. At the house, the windows should be closed with gauze wire, there should be a pit for disposing wastes, the stagnant water should be buried as well sleeping under mosquito nets.

**Facilitator** Alright. That is P1’s opinions. Let us hear other opinions. Can you explain what you know about House Improvement as one way of preventing malaria. P4?

**P4** If you are frequently improving your house, there will be no mosquitoes in the house and will be sleeping peacefully. However, if you are not frequently checking the house, there might be a spaces through which mosquitoes can enter the house. That is why we need to be checking the house frequently.

**Facilitator** Alright. Other opinions?

**P5** I would like to add on to what P4 has said. That’s a good idea. It is important to frequently check the house, close the windows, eaves as well as any spaces to prevent mosquitoes from entering the house. Mosquitoes bring malaria frequently. That is why we need to close all the spaces in the eaves. Mosquitoes should not give us diseases. The disease is malaria.

Reference 3 - 1.33% Coverage

House Improvement helps us prevent malaria. When you close all the spaces properly, mosquitoes from the bushes do not find a way into the house. They instead land somewhere outside. That way you prevent malaria.

**Facilitator** Alright. That’s P1’s opinion. Is there any addition? P6?

**P6** House Improvement protects us against malaria. Mosquitoes are very dangerous. When we are doing House Improvement we should also be sleeping mosquito nets. This is because when you are entering the house, the mosquitoes also enter through the open door. So when you sleep under mosquito net you protect yourself from malaria.

Reference 4 - 7.52% Coverage

Alright. Let us continue with our discussion. I would like to know from you as people coming from different villages. I would like every one of you to explain to me the ideas from their village. In your own thinking, what is the people’s attitude towards the House Improvement work in your villages? ***(Question repeated for clarity).*** Let us start with P7.

**P7** People in our village are differentiating current situation with the previous days. We are seeing the advantages of House Improvement. We are preventing a lot of things in the house. Everyone is saying things are currently better it was previously. Snakes cannot enter the house.

**Facilitator** Alright. That is P7’s opinion. Let us hear from P6.

**P6** The thing is, people in the village were not trusting this work when it was just coming in. When the committee members told them what to do, they would not take it. Later on, when they gathered the people and taught them the importance of House Improvement, people understood and started doing the work. So, many have now done this work and you cannot find a house which has not been improved.

**Facilitator** Alright. On the same issue, would you explain what people in the village were saying about House Improvement work or what were their expectations of the House Improvement work?

**P4** People were saying that when the committee members are giving them the gauze wire, they would also give them the nails for tacking the gauze wire. If they have only been given the gauze wire, they would argue. However, as P6 has said, when the committee members came to explain to the people that they only give gauze wire and not the nails. The owner of the house should be responsible to get the nails. They were telling them that they can get bicycle spokes, cut them into small pieces and use to tack the gauze wire. If they cannot manage, the committee members would help them to tack the gauze wire.

**Facilitator** Let us hear from other villages. What were people saying about the House Improvement work? Let us hear from P8.

**P8** When the House Improvement work was starting, people saying a lot of things. People were saying that these people are giving us a lot of work. Getting the soil from outside into the house and closing the spaces is much work. They were saying they could not do that. Later on, when we had exemplary houses in the village, people started appreciating its importance that mosquitoes were not getting into the house.

**Facilitator** Thank you very much. Let us continue our discussion. Do you think people in your village accepted House Improvement as one way of reducing malaria?

**P5** Thanks very much. Some people have learnt a lesson because mosquitoes enter the house quickly when you leave spaces in the windows. So, many people are glad that they have closed the spaces in their houses and mosquitoes cannot enter the houses quickly… they sleep peacefully without any diseases. They are thankful and this will continue.

**Facilitator** Alright. Let us hear from P6.

**P6** People received this project. They are appreciating that they have learnt through this project. They are thankful to those who brought this project.

**Facilitator** Alright. Did people in your village accept this work as one way of preventing malaria?

**P2** Yes, many people accepted this method of reducing malaria . Sleeping under mosquito nets. Many people who have improved their houses are appreciating the importance of this work that they are not suffering

Reference 5 - 2.51% Coverage

Alright, let us hear from other villages. What were the grievances or problems about the House Improvement work that people were saying? Yes, you raised your hand.

**P5** Women in our village were saying they did not want to close the eaves because they needed light to be given into the light at dusk. The committee members were telling them that mosquitoes enter through the open spaces. They were telling them to close the spaces to prevent entry of diseases. The disease is the mosquitoes that enter the house…

**Facilitator** Alright. P4, do you have additions?

**P4** No.

**Facilitator** Alright. We have heard grievances from two villages. What were the grievances in other villages? P1?

**P1** People were saying that there was not enough ventilation in their houses when they closed the eaves. So, they would not close the eaves with the gauze wire. They also wanted light to be getting into the house through the open spaces.

**Facilitator** Alright. So, what do you think can be done to sort out these grievances?

**P1** What can happen is that the committee members should teach these people the importance of House Improvement and sleeping in improved houses.

Reference 6 - 0.65% Coverage

Alright. I would like to hear for the last time; how can the methods of House Improvement that have been in use be promoted? What is supposed to happen?

**P4** The owners of the house should be checking their houses to make sure there are no spaces and the gauze wire is intact besides the committee.

Reference 7 - 2.66% Coverage

Alright. I would also like to learn from you on the same issue. In your own thinking, is there any relationship between the House Improvement and sleeping under mosquito nets? ***(Question repeated for clarity)***. P6?

**P6** I think there is no relationship.

**Facilitator** Alright. Would you explain?

**P6** We do not use mosquito net when improving houses. We do see some people tack mosquito nets in the windows when they do not have the gauze wire. However, they do not need to use mosquito nets in House Improvement.

**Facilitator** Alright. That’s P6’s opinion. What do the other say? ***(Question repeated for clarity).***

**P2** The advantage is sleeping under mosquito net. When you sleep under mosquito net, you sleep without mosquitoes. That is the advantage of mosquito nets; preventing malaria. Mosquitoes give us a lot of malaria.

**P1** The relationship is that when mosquitoes enter the unimproved house and you sleep under mosquito net, the mosquito net protects you from the mosquitoes.

**Facilitator** Alright. Any other opinions?

**???** There is a relationship. Closing the spaces, tacking the gauze wire and sleeping under mosquitoes all prevent malaria. So, there is a relationship because they are all helping to prevent malaria.

Reference 8 - 2.65% Coverage

Alright. Let us continue on the same issue. I would like to hear from you. In your own thinking, do you think after House Improvement people think sleeping under mosquito net is still important? ***(Question repeated for clarity).*** Let us start with P5 then we go to P7.

**P5** It is important to sleep under mosquito net because the doors are sometimes left open during the day and mosquitoes can enter the house. The you close the door at night, the mosquitoes are inside the house. The mosquito net prevents the mosquitoes from giving us malaria.

**Facilitator** P7?

**P7** Repeat the question.

**Facilitator** I was asking that in your own thinking, do people think sleeping under mosquito net is still important after they have improved their houses?

**P7** Yes, it is important. If the house in improved, we should also be protected by sleeping under mosquito net. This prevents the mosquitoes from biting us and giving us malaria.

**Facilitator** Alright. That’s P7’s opinion. Let us hear from P1.

**P1** When you improve you, you still go out at night to urinate and mosquitoes get into the house. So, if you do not sleep under mosquito net, even if the house is improved, the mosquitoes that have entered the house can give you malaria.

Reference 9 - 3.73% Coverage

Alright. Is there any addition on this issue? Alright, let us finish this section by asking this question; in your own thinking, have people in your villages realized that leaving eaves open contribute to spread of malaria? ***(Question repeated for clarity).*** P3, do you have anything to say?

**P3** No.

**Facilitator** Alright. Let us start with P6.

**P6** People have realised that House Improvement is important. Previously, people were saying this is not true. They understood after they had been taught its importance. This project has reduced malaria prevalence so they are agreeing that improving houses is important. Many people in our village are improving their houses.

**Facilitator** Okay. I would like to hear from you. How do the open eaves help in the spread of malaria?

**P6** Mosquitoes enter through this eaves and bite people inside making them suffer from malaria.

**Facilitator** Alright. That is P6’s opinion. Let us hear opinions from elsewhere. Have people in your village realised that leaving open eaves contribute to spread of malaria?

**P4** I this it contributes to spread of malaria because they were differentiating those with improved houses and those without improved houses. They were frequently suffering from malaria. This is because the mosquitoes give a lot of malaria. If it bites someone with malaria and then someone without malaria, you get malaria.

**Facilitator** Alright. P8?

**P8** The disadvantage of not improving houses is that you suffer from malaria frequently. Additionally, when one child falls sick, the other one follows. Since we closed the eaves, there has been reduction of malaria in our household. People started admiring how the work was done and how we are getting malaria nowadays.

<Files\\FA-C HI Committee members FGD> - § 9 references coded [18.15% Coverage]

Reference 1 - 0.82% Coverage

the work has been going on well because we were trying to reduce the number of mosquito that get in the house. When the project came, with our chiefs, we welcome the project and everyone accepted it knowing that the time they could spend is less than the money they could spend to treat malaria

Reference 2 - 1.02% Coverage

when this project came, we called for the village meeting to explain to them the advantage of closing of open eaves, people received the project. Sometime we could give the gauze wires to someone, he could tell us that he/she does not have the nails to use, that’s the problem we were facing. What we were doing was to take the wires and cut them to use as the nails.

Reference 3 - 2.91% Coverage

okay, do you thing this method of house improving is good for malaria prevention?

**P3:** this is right method because the time this method was not used, malaria was spreading everywhere. When this project started, malaria is reduced

**P8:** this method is more effective but the thing is that chiefs and the community members should work hand in hand for this project to be successful

**P7:** to on what my colleagues have said. It’s our plea to the organization that brought this project, you should reach to others where this project is not yet there and in so doing, here at Chapananga, issues of malaria will be the history. For now, only a few villages are secure but when the project is extended to the other villages, malaria will be the history here at Chapanga

**P6:** I would like to what others have said on malaria prevention through gauze wires. This gauze wire has helped us and most of community members are thankful. Before gauze wires, people were catching malaria easily but after this project, this is now reduced and everyone is thankful

Reference 4 - 1.13% Coverage

thank you. Let’s go to the other section. As committee members, can you tell me what you know about HI as one way of preventing malaria?

**P8:** what happens with HI is to close open eaves so that there should not be the gap for the mosquito to access the house. When there is no gap, the few mosquito which is inside, cannot bite people if there is mosquito net and hence, a person is protected from malaria

Reference 5 - 0.85% Coverage

house improving helps us to prevent malaria. If the house has got holes, mosquito that causes malaria get in the house. We produce smell and the mosquito follow that smell. When we close the house, the mosquito does not get in the house, our smell does not get outside and in so doing, we prevent malaria

Reference 6 - 1.33% Coverage

okay. What do you think people think about this house improving work? What are people’s expectations on this work?

**P7:** Before people knew the importance of it, they think that they could not afford it. Onn weakly basis, 2 children could suffer from malaria but after this house improving work, when we started inserting the gauze wires, the issue of malaria is no longer the issue. There is less mosquito getting in the house through the door because it cannot use the window

Reference 7 - 4.46% Coverage

people said that they did not trust this before but now they trust it because malaria is no longer an issue. Everyone is expecting that malaria here at Chapanga will no longer be an issue

**P3:** as my colleagues said that people had doubts before, simply because they did not know the benefits. When the close all open eaves, the money that could be used for medical bills is used for other task at home and the development is easy because malaria was taking a lot of money, when three of four people get sick of malaria, it was a lot of money. People are now able to come and report if the gauze wire is damaged

**P5:** Those people who have got houses with unburned bricks could build their houses anyhow. When this project came, we improved 2 sample houses in our community so that everyone should do the same

**Facilitator:** Do people in your communities receive the HI as one way of preventing malaria?

**P4:** people in our community received this and they see change, they now practice hygiene

**P2:** I think people have welcomed this and they are happy with this because there is no problem and it’s our plea that these organizations should continue helping us so that malaria should be reduced

**P7:** people in our community welcomed it and it was not expected. We were thinking that only people with iron sheets houses close open eaves, for those with glass thatched houses do not do. When everyone saw that even the glass house that can happen, it was something special here at Chapanga. Everyone was happy to have been learning something and if this continues, malaria will no longer be an issue here at

Reference 8 - 2.89% Coverage

okay, do you think there is any connection between the house improving (HI) and the use of mosquito net?

**P3:** there is connection between house improving and the use of mosquito nets. The relationship is that mosquito can get in the house when we have opened the door and if we don’t sleep under the net, we can catch malaria despite that we have closed all open eaves, if we have slept under the net, we can be protected from malaria

**P8:** There is a very big relationship because we can do everything like closing of open eaves and inserting the gauze wires but if we have left the door open, mosquito can get inside and if there is no net, it’s easy to catch malaria

**P7:** the relationship is very big because when we closed the open eaves but nevertheless, mosquito gets in the house but the mosquito that get in the house through the door, it’s few and when it gets in the house, it will find you sleeping in the second house. When we say second house we mean under the net, so it cannot get you. these 2 tasks should go together

Reference 9 - 2.74% Coverage

community members have realized that if the open eaves are not closed, we have given the way to the mosquito to get in the house and if we don’t sleep under the net, we can catch malaria.

**P3:** People have realized that leaving the open eaves unclosed can cause malaria because they have compared with the time they were leaving open eaves unclosed, they were getting sick but after closed the open eaves, they have reduced the time they were going to the hospital

**P7:** They have seen the disadvantage of leaving the open eaves unclosed because the way how mosquito gets in the house now and the time the open eaves were not closed is different, that time lot of mosquitos could get into the house than this time because it is using the door

**P4:** It facilitates because that time less mosquito could get in the house than now and there is mosquito net, things have changed now

**Facilitator:** How do you see the house improving task?

**P3:** At first, we thought this job is difficult because

<Files\\GVH KABWATIKA FINAL.> - § 3 references coded [9.33% Coverage]

Reference 1 - 1.22% Coverage

Alright. How does house improvement prevent malaria?

Respondent: It prevents mosquitoes from entering

Reference 2 - 6.47% Coverage

How would you describe community perception of house improvement in your area chief?

Respondent: Community perceive this activity as one of the good intervention towards malaria spread.

Interviewer: What are the comments and expectations made about the intervention?

Respondent: Comments…. comments

Interviewer: What are the comments and expectations made about this intervention from your community?

Respondent: The comments are like the intervention has done well to them because of reduced malaria cases than before, as of now everything is good.

Reference 3 - 1.63% Coverage

Firstly, they are preventing over expenditures due to frequent hospital visit. The good thing is that their health status has been improved.

<Files\\GVH KAMOGA> - § 7 references coded [15.28% Coverage]

Reference 1 - 2.61% Coverage

could you please describe what you know about House Improvement as a method of preventing malaria?

Respondent: **yes, first we close the eaves and close all holes properly and cross check if there is no hole that the mosquito can use as entry point and find gauze wire and close the window eee that what we do.**

Reference 2 - 2.62% Coverage

How would you describe community perception of House Improvement activity in your community?

Respondent: **they warmly welcomed this project and they see things changing because in past time people were falling sick frequently. Once this program started people see it as a good program with positive results.**

Reference 3 - 0.95% Coverage

**aaaa most likely there are no problems when (inaudible voice) they I understood and all the things are okay. Sure**

Reference 4 - 1.86% Coverage

in your opinion, how can you improve this house improvement strategy ?

Respondent: **aaa like we are.. we are promoting like those newly build house we tell them like they should be following what we did to those old ones**

Reference 5 - 3.06% Coverage

*mumu..* in your opinion is there any relationship between improving the houses and using nets?

Respondent: **yes there is the link eee like when improving the houses we shouldn’t what, we should not stop using nets. If we may stop things cannot be alright malaria can what, malaria can start over. So the nets we should what, we should continue using nets eeee**

Reference 6 - 2.82% Coverage

**yes there are much aware, and we don’t have any person who is troubling us. Everyone in the community see this as acceptable.**

Interviewer: how does open eaves promote malaria spread

Respondent: **mosquitoes enter using those eaves sure eee they use eaves, that is why we close the eaves so that there is no any openings on the walls.**

Reference 7 - 1.35% Coverage

**aaa it is tiresome aaa because we draw water, making bricks aaa burning them. Aaa it is tiresome only that if the person needs the thing he make it very possible**

<Files\\GVH MAKANDE(1)> - § 7 references coded [20.88% Coverage]

Reference 1 - 3.60% Coverage

alright thank you. Could you explain what you know about house improvement as one way of preventing malaria?

Respondent: Yes I can explain

Interviewer: Pleases explain

Respondent: Once Hunger Project came in, the other organisations also came and start teaching people on improving the house, and then people really followed as a result malaria incidence decreased

Reference 2 - 1.95% Coverage

Alright. How does house improvement prevent malaria?

Respondent: This prevent mosquito from entering the house and also should not bleed because once we close there is no entrance for the mosquito.

Reference 3 - 1.85% Coverage

Alright thank you. How would you describe community perception of this activity in your community?

Respondent: aaaaa people welcomed this activity because a lot of houses have closed eaves.

Reference 4 - 6.08% Coverage

Alright I want to know, do you think this intervention has been accepted mmmm in your area as one way of preventing malaria?

Respondent: Yes they accepted.

Interviewer: Please explain

Respondent: [sighing] people welcomed this intervention, community selected committee members, once they were selected they started working by receiving gauze wire and walking around the village since that others were still in denial hence the committee members would have that power and close the house and making the house improvement strategy eeee. Others accepted this intervention and they are doing on their own to prevent malaria

Reference 5 - 0.97% Coverage

what are the negative issues people in your area say towards this intervention?

Respondent: None.

Reference 6 - 1.57% Coverage

How do you think can this house improvement be improved?

Respondent: This can be promoted by chiefs encouraging people so that people can be thinking forward.

Reference 7 - 4.87% Coverage

Alright. In your opinion how does house improvement interact with indoor interventions like mosquito nets?

Respondent: Yes there is a link.

Interviewer: (stammering) in your opinion do people in improved houses feel that they no longer need to use bed nets?

Respondent: Yes it is important

Interviewer Please explain

Respondent: The way we are been taught is that once you close house eaves you are also supposed to sleep in mosquito nets since that it is the protection to un born baby.

<Files\\HSA Bwalo Transcript KII> - § 10 references coded [36.24% Coverage]

Reference 1 - 8.38% Coverage

OK, in the first place when this method was introduced in these villages, there were volunteers that were selected to take the lead role and these are called health animators. These animators provide education to the community on how to do house improvement, in newly built houses and old houses on how they can close the eaves. They encourage people to close the eaves in their houses so that malaria can be prevented. These volunteers are based here in the villages, and as HSAs, we’ve met them and explained to them that sometimes it could be difficult for the community to understand you so we sometimes go around the community with them as they are delivering health talks pertaining to malaria. So it appears people has understood this house improvement concept as they have now improved most of their houses.

**Interviewer**: Thank you, so in your thinking, how does HI prevent malaria?

**Respondent**: This intervention is helping in malaria prevention because the time a person improves their house, firstly it means that there will not be room for mosquito entry, so as a result this reduces cases of malaria. I am saying this because I am one of the people who resides within the village, and I have told you in the first place that I test malaria in under-five children, and if you can look at last year’s data from January to December, maybe it’s only around 20 people that had malaria compared to previous years I could see over 100 people per month and within the hundred, 5 were found with malaria. So in my opinion I think that there is a big improvement when it comes to the malaria problem, in terms of reduction of malaria cases due to house improvement.

Reference 2 - 4.79% Coverage

**Interviewer**: So as the HSA of these 2 villages, how would you describe community perception of HI activity in your area?

[**Probe**: What are the comments and expectations generally made about the intervention?]

**Respondent**: At first it was difficult to tell people to do house improvement because they would build a house without closing the eaves and a majority complained of lacking bricks which was difficult for them to build houses. Another thing was that here in Chikhwawa it is very hot so many people took it as a style of building a house with open eaves as a way of minimizing this heat. However when this issue of HI was introduced, and after the community was told about the advantages of having HI and closing eaves, little by little people began to accept it and they saw it as a good thing. So it is been found out that apart from the heat we have here in Chikhwawa, people have accepted HI with the fact that they will prevent malaria.

Reference 3 - 3.56% Coverage

**Respondent**: OK, for the comments that were mostly made, people are very happy because the way they used to stay back then without closing eaves with now when they started closing the eaves, people expressed happiness because firstly, malaria cases are minimal, people are less sick. If they were sick they would be going to Kapichira clinic and pay for the health services but right now this has actually reduced. Very few cases are going there at the clinic. So this money that is saved by not going to the clinic is now being used for other household developments. People are indeed very happy about the introduction of this intervention in the villages because it has helped their households to develop.

Reference 4 - 2.51% Coverage

Yes, complaints indeed will always occur, for instance at first the gauze wire which we received for the windows underwent rusting so people complained that the wire was going bad and was rusting. Apart from that people that had newly built houses and those in old houses who wanted to build new houses when they told the HI committee that they were looking for wire gauze, they received a response that wire gauze is no longer in stock. So some of the complaints that were received are these ones.

Reference 5 - 2.54% Coverage

So like for us, we normally communicate with the health animators, because they are the ones who are looking after this job and are residing in the villages, of course there is also the HI committee. So we normally conduct a village meeting together with the animators and the HI committee so that they can hear the people’s grievances. Whatever is being discussed at those meetings are taken by the animators and we ask them to deliver to the people responsible from MMP in order to get a right response.

Reference 6 - 3.98% Coverage

Ah, I shouldn’t say that the concerns are demotivating the participation because lie for the many people that have damaged wire gauze, it’s only rusting that occurred but not perforations. So what we normally tell those people is that they should not remove the wire, it should remain there on the window up to the time when we receive the new wire gauze. Also for those that haven’t received any wire gauze we tell them to close the eaves on their houses and to put some damaged gauze if available by any chance as we are waiting for the new gauze. We do this so that they should not become complacent about the situation but they just have to do what they can in order to prevent malaria. The items that will come from the project will be like additional items to what we have already done.

Reference 7 - 1.16% Coverage

The other way that I see is that when people are building their own houses we encourage them to build the house having well-built windows with glasses. Apart from this what we tell them is that they should sleep in mosquito nets.

Reference 8 - 3.80% Coverage

So in your thinking, how can the strategy of implementing HI be improved?

**Respondent**: It can be improved if the materials are available such as wire gauze, sometimes we have new houses that have been built but there is no wire gauze. This demotivates the people because they always hear that they should improve their houses and yet the materials are not available. But if wire gauze is readily available, people will be eager to build houses so that they receive the wire.

**Interviewer**: In your opinion, how does house improvement interact with indoor interventions like bed nets?

**Respondent**: Yes, it is there. Because the time people are doing house improvement we also encourage them to sleep under a mosquito net every time and throughout the year.

Reference 9 - 2.33% Coverage

: Do people in HI houses feel that they no longer need to use bed nets?

**Respondent**: Yes, because of the information we give them they sleep under the mosquito net. Apart from them having an improved house, we also receive nets and we distribute them to the community. This helps them to question the significance of having the mosquito net with them. They would have that encouragement that if I have improved my house I should also be sleeping in a mosquito net.

Reference 10 - 3.18% Coverage

**Interviewer**: Do you think that your community is now more aware about the risk of open eaves for malaria transmission?

**Respondent**: A lot. We have educated them that when they are leaving open eaves they are giving chance for malaria mosquitoes to enter the time they are sleeping at night but when they close the eaves, mosquitoes do not have the chance of house entry. We tell them this because as you may know Chikwawa is very hot so this issue of closing eaves always comes up by the community as it is a hot place and they would complain. We have managed to convince them and they no longer complain about feeling hot inside.

<Files\\HSA Liwonde- Kapichira Health Center KII> - § 9 references coded [30.16% Coverage]

Reference 1 - 4.21% Coverage

As a Health Surveillance Assistant, can you explain to us what you know about House Improvement as one way of preventing malaria?

**HSA** House Improvement is a good way in which people are told to close or spaces, and windows with gauze wire, to prevent entry of mosquitoes into the house. It is one method I have noted that has helped a lot in reducing the prevalence of malaria. However, the gauze wire we were using then was getting damaged by rust. We are currently working on replacing the old damaged gauze wire with the new one. That is the work being done currently.

Reference 2 - 3.83% Coverage

Alright. In your thinking, what is the attitude of people in your village towards the House Improvement work?

**HSA** At first, people seemed to be reluctant because there were some required materials which could be sourced locally but they were waiting for the Majete Malaria Project team to be bringing them such materials as nails. When we discussed with the animators and project committee, people understood that on their own they had responsibility to make sure the work is done by sourcing such materials as nails.

Reference 3 - 2.96% Coverage

People are currently happy and interested in the work. This is the reason at the start of the work all the houses were improved. However, the wire we used at first was getting damaged with rust and has to be replaced in other houses. Additionally, the recent rains damaged houses and has affected the work because people have to start maintaining these houses and we can close the windows thereafter.

Reference 4 - 3.02% Coverage

Many people accepted this work. As I have pointed out already, we improved many houses which indicates that many people accepted the work. Additionally, people could testify that since the start of the work there was reduction in the number of mosquitoes entering the houses as well as the frequency with which people were suffering from malaria in households compared with the time before House Improvement.

Reference 5 - 1.31% Coverage

House Improvement is the main method in this area; closing spaces with mud and bricks. People in this area cannot manage using cement to close these spaces in as far as I know.

Reference 6 - 3.24% Coverage

Alright. In your opinion, how can the House Improvement methods that have been employed so far be promoted?

**HSA** Firstly, there is need to ensure that such materials as gauze wire required for House Improvement should be available. Additionally, the project committee should be encouraged to be monitoring the progress of the work. This will make them dedicate themselves to the work because will be seeing that the work is really happening

Reference 7 - 6.08% Coverage

Alright. In your opinion, is there any relationship between House Improvement and use of mosquito nets?

**HSA** Yes, there is a relationship. This is because we close the space and windows with gauze wire but we cannot close the door with gauze wire. There will be no way into and out of the house. Therefore, some mosquitoes enter the house through the open door. This means that of people in the house do not sleep under the mosquito nets, we will have failed to prevent malaria because they will be infected by the mosquitoes. However, if we close the windows with gauze wire, mosquitoes will not enter house through the windows. If mosquitoes enter through the door, the person will be protected if they sleep under the mosquito net. This means mosquitoes will get house of the house because there will be no food to feed on.

Reference 8 - 2.99% Coverage

Alright. I would also like to know your opinion on this same issue. When people have improved their houses, do they think it is still necessary to sleep under mosquito nets?

**HSA** Yes. We give out messages about this and people follow that House Improvement is not 100% perfect in preventing malaria but they also have to be sleeping under mosquito nets so that we are protected every day and every night.

Reference 9 - 2.52% Coverage

Alright. In your opinion, have the people in village realised that leaving open eaves help in the spread of malaria?

**HSA** Yes, they are now realising this because of the messages with have been giving out through meetings and door-to-door approach. As such, many people currently are closing the eaves whenever they are constructing houses.

<Files\\KII Chief Chambo FA-C> - § 10 references coded [19.64% Coverage]

Reference 1 - 0.74% Coverage

like improving using the wire, closing the eaves, looking for and closing the cracks with mud

Reference 2 - 2.39% Coverage

how house improvement protects us from getting malaria?

Respondent : Improving the house protects us much since the mosquito that spread malaria pass through the unclosed eaves and the mosquito look for the cracks to enter the rooms and find us since the mosquito can smell where people are sleeping.

Reference 3 - 1.73% Coverage

How do you think people perceived this work of improving the house?

Respondent : this work was tiresome at first because we were ignorant. Now that we know that this work is good, no one is hesitating to do this job

Reference 4 - 1.97% Coverage

Do you think people in your area received this method of improving houses as one way of reducing malaria?

Respondent : People received it, at first people were tough but little by little people started to understand, no one is tough in our village

Reference 5 - 1.96% Coverage

: There is nothing negative that people say about house improvement or searching for the cracks, but the problem is the person failing to understand, but they are not bad. There is no any negative thing or any problem that come due improving houses

Reference 6 - 3.15% Coverage

mmm… this method… it is difficult

Interviewer : All right, what do you think the strategy, which is been followed in improving the houses being improved?

Respondent : This work can been improved if the top bosses follow up, and as chiefs we are working with the animators and leaders in the villages who were selected, we are leading those people so that the work should make a good progress

Reference 7 - 1.62% Coverage

thank you very much, in your opinion, is there any interaction between house improvement and the use of mosquito nets.

Respondent : Yes, there is an interaction, since all of them are preventing malaria

Reference 8 - 2.07% Coverage

So in your opinion, are people supposed to sleep under mosquito nets after improving their houses?

Respondent : Some think it is not necessary, but it is necessary to do both of them, if there are mosquito nets in your house, you are supposed to use them

Reference 9 - 2.54% Coverage

All right, do you think that your community is now aware that leaving the eaves open increases the spread of malaria?

Respondent : Yes, according to what they have learnt in malaria project. People have learnt and know the importance of improving houses, because when we improve the houses, malaria is been reduced

Reference 10 - 1.47% Coverage

How do open eaves facilitate malaria transmission?

Respondent : The open eaves facilitate because the mosquito passes through them to enter our rooms and find where we have slept.

<Files\\KII Chief Goliati> - § 10 references coded [19.86% Coverage]

Reference 1 - 1.81% Coverage

mmm Can you explain what you know about house improvement as one way of preventing malaria

Respondent : Ahhh alright, aaah thank you very much, the method that I know is that when closing the windows, first of all the eaves should be closed and the committee is trying to tell people to close such places, helping those who are unable to use the wire gauze. The committee is committing itself in improving the houses

Reference 2 - 1.63% Coverage

Alright, I also want to learn, how house improvement helps us to prevent malaria

Respondent : house improvement helps to prevent mosquito from entering the house

Interviewer : mmm

Respondent : because mosquito searches for spaces to enter the house and bite the person

Interviewer : mmmm

Respondent : so when we close those spaces, we are done with the mosquito

Reference 3 - 3.45% Coverage

: In your opinion, how do people perceive this work of house improvement

Respondent : Alright, people are happy with this work of house improvement. No one is sad

Interviewer : What comments and expectation that people say about this house improvement strategy

Respondent : People say that when there is a meeting,

Interviewer : mhmm

Respondent : so people tell the committee which was selected

Interviewer : eeeeh

Respondent : Telling them to make sure that they visit the households to identify the house which has not been improved

Interviewer : eeeh

Respondent : And that they should ask materials for newly constructed houses so that all the houses should be the same because when some people are sleeping in unprotected places, disseises will not end in our community

Reference 4 - 2.04% Coverage

They received it very much, they received it fully

Interviewer : mmm

Respondent : even in my assessment on how they received it

Interviewer : mmm

Respondent : I have found that every household is improved, when the wire gauze has been damaged, the report is sent to the committee, when the windows are damaged, the committee count the number of houses with damaged windows

Interviewer : mmmm

Respondent : so we see that people have received it with both

Reference 5 - 2.88% Coverage

What are the negative things or the challenges which people talk about this house improvement strategy

Respondent : The challenges were there at the beginning of this program

Interviewer : mmm

Respondent : some were saying, they don’t find a meaning in closing the windows

Interviewer : mhm

Respondent : so we had to organise the meeting with the committee and alert the people

Interviewer : mhmm

Respondent : we explained to the them about the problem and ways of solving it. I told them that anyone who will reject this should move out of my village so that we should see the benefit that will come if everyone has improved his or her house.

Reference 6 - 1.81% Coverage

the concerns about the damaging of the wire gauze are encouraging by telling the committee to request for the resources. There are some newly constructed houses and others have fallen due to rainfall

Interviewer : mhmm

Respondent : these are the concerns which have been most reported to the committee to make haste

Interviewer : mhmm

Respondent : the wire gauze is becoming damaged when the houses fall

Reference 7 - 1.19% Coverage

: do you think there is any interaction between house improvement and the use of mosquito nets?

Respondent : there is an interaction, they are being done simultaneously, closing the windows with wire gauze and sleeping under mosquito nets should be done at the same time

Reference 8 - 2.15% Coverage

Alright, what is the interaction between the two

Respondent : the interaction that we agreed, is that when we have received the nets the committee assures us that they will be supervising to see if the nets are being utilised. I also encourage them to move around and to report to me those who refuse them from entering their houses so that I can call the person

Interviewer : mhmm

Respondent : to ask him if he sold the net or is misusing it or why is he rejecting the committee to see

Reference 9 - 1.25% Coverage

have improved their houses.

Respondent : yes, they find it very necessary

Interviewer : why

Respondent : because when we use the mosquito nets after we have closed the windows, we are not worried even if the mosquitoes have found its way through the door, it will find us in nets

Reference 10 - 1.65% Coverage

Alright, do you think people in your community are aware that open eaves increase the spread of malaria?

Respondent : they are very aware

Interviewer : how open eaves increases the spread of malaria

Respondent : open eaves allow the mosquito to enter, because when the mosquito wants to enter the house, it searches from the bottom up to the eaves where they enter if open

<Files\\KII Kalinjala> - § 3 references coded [8.64% Coverage]

Reference 1 - 4.78% Coverage

do you think people in improved houses feel that they no longer need to use mosquito nets?

Respondent: we always advise them that it is very important

Interviewer: uhmmm explain

Respondent: it is important that even though we have closed the windows with gauze wire but mosquitoes may enter using the door while it is open, so we need to sleep in the mosquito nets.

Reference 2 - 2.91% Coverage

do you think that your community is now aware about the risks of open eaves

Respondent: yes they know

Interviewer: how do open eaves facilitate malaria transmission.

Respondent: mosquito use the open eaves as the entrance

Reference 3 - 0.95% Coverage

it is good even though it is tiresome, however people accepted it fully.

<Files\\KII MACHEKERO(1)> - § 11 references coded [19.35% Coverage]

Reference 1 - 0.92% Coverage

thank you for everything that has happened in my village, that’s very good and when they explained to us through the committee, people understood and started using gauze wire, house improvement strategy and sleep under mosquito net

Reference 2 - 3.71% Coverage

can you explain what you know about house improvement as one of the method of preventing malaria?

Respondent: aaaaah on the issue of house improvement, as one way of preventing malaria, is the one I was explaining that, because the program of house improvement was introduced, we used to suffer different kinds of diseases because we were being bitten by the mosquito. It was possible that you wake in the morning with spots signifying that you have been bitten by mosquito, but since the program of house improvement was introduced, most of the people who understood what there were saying, even myself understanding what the committee said and believed that this is true, I notice some changes. Eeeee, there is change unlike in the past. They are changing in the village.

Interviewer: fine

Respondent: eee and it’s a wish that although this is a foreign program, I would love that it should continue so that we benefit in our village

Reference 3 - 1.20% Coverage

comments about house improvement intervention?

Interviewer: eeee and expectations

Respondent: aaaaaa when you explain to them, like the committee, they understand better. Yes ….. saying that the work that you would give to us, say do this, we will do the job because it is benefiting our own lives.

Reference 4 - 1.86% Coverage

after they received the method of using gauze wire they showed some attention to this issue and understood

Interviewer: mmmm

Respondent: meaning that after understanding malaria is caused by mosquitoes and after they heard about this they started to understand that mosquito do infect people with malaria

Interviewer: mhhh

Respondent: and we are suffering because of that and we need to change our thinking and follow what is being said now by the malaria team

Reference 5 - 3.14% Coverage

and we started explaining to them and being the chief I called for a meeting asking them, have you understood what my committee has been telling you in this village? Eeehhh. The issue is that we should change our thinking. We should leave our old ways of doing things and start afresh.

Interviewer: eeehhhh

Respondent: the first thing is that we were losing our lives frequently due to malaria, and we did not know the causes of the deaths and if there has come a way of dealing with malaria, in other countries, malaria is no longer there after following a similar intervention. So if we also follow a similar way like this, maybe we can also change things in our village. After understanding that if the chief and her committee are saying this, it could be possible that they are right thing

Reference 6 - 0.35% Coverage

aaaah I have not heard anyone in my village commenting bad things about the project. No

Reference 7 - 1.52% Coverage

eehhh. They would have said chief, we have faced such a problem and we will not manage but there is no one who complained. And as I am speaking, some of the gauze wire has worn out and now they are asking when they will be given another one

Interviewer: aahhhh

Respondent: so if they had faced some problems they would not have telling me that the wire has worn out, people are happy

Reference 8 - 1.22% Coverage

think on our own we cannot manage, we need helpers, those who help should continue so that we should be protected because we cannot who much the wire cost and other things.

Respondent: mhhh

Respondent: because we can afford some meters of the wire own your own since this is the village. Money is scarce.

Reference 9 - 0.70% Coverage

it is good that the net protects us from malaria. The use of gauze wire is the same thing. If the program continues, we may have good lives because we can prevent so many things

Reference 10 - 1.63% Coverage

aaa yes..

Interviewer: in your own opinion, if people have renovated their houses, do they still think it is important to use mosquito nets?

Respondent: yes, it is possible. What we have said that you have renovated the house, and put gauze wire and you don’t have a bag to put your clothes, or that either children opened the door and mosquitoes find their way in the house, in this case, a net could be used

Reference 11 - 3.11% Coverage

in your opinion, do people realize that leaving gaps between the wall and roof help to transmit malaria?

Respondent: realizing that leaving space between the wall and roof?

Interviewer: yes

Respondent: aahhh no. it doesn’t help

Interviewer: leaving the gaps between the wall and the roof the house, have they realized that it helps to transmit malaria?

Respondent: yes they have realized

Interviewer: how have they realized?

Respondent: they were living in ignorant not knowing the impact of leaving the gaps, and after noticing that they frequently suffering from malaria and they were found with malaria at the hospital, because of this, they know that the mosquitoes enter through the gaps between the roof and the wall of a house and bite them there by escalating malaria.

<Files\\KII MDZACHI(1)> - § 7 references coded [6.90% Coverage]

Reference 1 - 0.56% Coverage

there is need for a toilet, bathing area, aaaaa pit latrine where we throw our wastes to prevent some diseases

Reference 2 - 0.72% Coverage

how does house improvement protect us from getting malaria?

Interviewer: mmm

Respondent: the mosquitoes are the ones that transmit malaria

Reference 3 - 0.61% Coverage

the process of improving the house, how do people see it? People in your village

Respondent: they see it as being good

Reference 4 - 0.98% Coverage

Respondent: they say I don’t have bricks and wait for some time when I am ready I will close the house

Interviewer: mmhh

Respondent: so we tell them to fasten the process and cover the house

Reference 5 - 1.03% Coverage

it can be promoted, boys come with a gauze wire while the chief is there and call the owner to tell him/her that your house is not covered and you should cover it. The aim is that we want to fix the wire

Reference 6 - 1.60% Coverage

in your opinion, do people in your village realize that leaving the space between the wall of the house and the roof help to transmit malaria?

Respondent: yes they are realizing that because they fall sick. So we say the suffering is as a result of not covering the house. Its mosquitoes that caused the suffering

Reference 7 - 1.39% Coverage

Respondent: mmm, because they tell us if we tell them to closing our house, they ask “have you bought nails?”,

Interviewer: mmmmh

Respondent: if you say I have not bought, you hear them buy them. How many should I buy? They tell you depending on the size of your window.
